# Multiomics Assessment of Gene Expression in a Clinical Strain of CTX-M-15-Producing ST131 *Escherichia coli*

**DOI:** 10.3389/fmicb.2019.00831

**Published:** 2019-05-03

**Authors:** Luís Pinto, Carmen Torres, Concha Gil, Júlio D. Nunes-Miranda, Hugo M. Santos, Vítor Borges, João P. Gomes, Catarina Silva, Luís Vieira, José E. Pereira, Patrícia Poeta, Gilberto Igrejas

**Affiliations:** ^1^Department of Genetics and Biotechnology, School of Life and Environment Sciences, University of Trás-os-Montes and Alto Douro, Vila Real, Portugal; ^2^Functional Genomics and Proteomics Unit, School of Life and Environment Sciences, University of Trás-os-Montes and Alto Douro, Vila Real, Portugal; ^3^Veterinary Science Department, University of Trás-os-Montes and Alto Douro, Vila Real, Portugal; ^4^Área de Bioquímica y Biología Molecular, Universidad de La Rioja, Logroño, Spain; ^5^Departamento de Microbiologia II, Facultad de Farmacia, Universidad Complutense de Madrid, Madrid, Spain; ^6^LAQV-REQUIMTE, Faculty of Science and Technology, Nova University of Lisbon, Lisbon, Portugal; ^7^Bioinformatics Unit, Department of Infectious Diseases, National Institute of Health, Lisbon, Portugal; ^8^Technology and Innovation Unit, Department of Human Genetics, National Institute of Health, Lisbon, Portugal; ^9^CECAV, Centro de Ciência Animal e Veterinária, Universidade de Trás-os-Montes e Alto Douro, Vila Real, Portugal

**Keywords:** bacteria, antimicrobial resistance, public health, genomics, transcriptomics, proteomics

## Abstract

Extended-spectrum beta-lactamase (ESBL)-producing *Escherichia coli* strain C999 was isolated of a Spanish patient with urinary tract infection. Previous genotyping indicated that this strain presented a multidrug-resistance phenotype and carried beta-lactamase genes encoding CTX-M-15, TEM-1, and OXA-1 enzymes. The whole-cell proteome, and the membrane, cytoplasmic, periplasmic and extracellular sub-proteomes of C999 were obtained in this work by two-dimensional gel electrophoresis (2DE) followed by fingerprint sequencing through matrix-assisted laser desorption/ionization time-of-flight mass spectrometry (MALDI-TOF/MS). A total of 602 proteins were identified in the different cell fractions, several of which are related to stress response systems, cellular responses, and antibiotic and drug responses, consistent with the multidrug-resistance phenotype. In parallel, whole genome sequencing (WGS) and RNA sequencing (RNA-Seq) was done to identify and quantify the genes present and expressing. The *in silico* prediction following WGS confirmed our strain as being serotype O25:H4 and sequence type ST131. The presence of proteins related to antibiotic resistance and virulence in an O25:H4-ST131 *E. coli* clone are serious indicators of the continued threat of antibiotic resistance spread amongst healthcare institutions. On a positive note, a multiomics approach can facilitate surveillance and more detailed characterization of virulent bacterial clones from hospital environments.

## Introduction

Rates of Gram-negative healthcare-associated infections have been increasing since 1998, mostly caused by antimicrobial resistant *Enterobacteriaceae*. A strikethrough recent worldwide survey revealed the prevalence of extended-spectrum beta-lactamase (ESBL)-producing *Enterobacteriaceae* in 14% of healthy individuals, a rate predicted to increase by 5.38% year on year overall (Karanika et al., 2016; Bassetti et al., 2017). Patients hospitalized in intensive care units and in long-term care facilities, or those who are immunocompromised have a higher risk of acquiring multidrug-resistant Gram-negative infections (Kunz and Brook, 2010). ESBLs are enzymes encoded by plasmid-borne genes, typically from the TEM, SHV, CTX-M families, that mediate resistance to oxyimino-beta-lactam antibiotics, third-generation cephalosporins and aztreonam (Rice, 2009; Drawz and Bonomo, 2010). For years, *Escherichia coli* producing the CTX-M-15 variant have been frequently implicated in human infection (Ewers et al., 2010). It has also been noted that the *bla*_CTX-M-15_ gene is located 49 bp downstream of insertion sequence IS*Ecp1*, a well-known highly efficient mobile element playing a major role in the expression and spread of CTX-M beta-lactamases, the most common in Europe (Peirano and Pitout, 2010; Guiral et al., 2011). Throughout the years, the ciprofloxacin-resistant CTX-M-15-producing O25:H4-ST131 *E. coli* clonal group is known to have caused major outbreaks worldwide (Nicolas-Chanoine et al., 2008; Ewers et al., 2010; Johnson et al., 2017). Classed as a member of the virulent phylogenetic group B2 and having the multidrug-resistant profile of the sequence type (ST) 131 clonal group, the O25:H4-ST131 clone represents a major public health problem as it makes it more complicated to select an appropriate therapy to administer, with a higher risk of increased costs and use of “last resort” antibiotics (Vimont et al., 2012). ST131 is therefore seen as being at the leading edge of a deeply concerning set of increasingly challenging infection agents (Vimont et al., 2012; Johnson et al., 2017). In the present work, we studied an ESBL-producing *E. coli* strain of human clinical origin, designated C999, that was previously studied and characterized by Ruiz et al. (2012). C999 was resistant to fluoroquinolones and third generation cephalosporins because of CTX-M-15 ESBL and belonged to phylogenetic group B2 and ST131. According to the genomic profile of *E. coli* C999 we assumed that this strain is related to the hazardous intercontinental O25:H4-ST131 clone. In our research, we took a multiomics approach to more deeply characterize this significant clinical strain. Whole-genome sequencing (WGS) and RNA sequencing (RNA-Seq) analysis were conducted to confirm if the *E. coli* C999 strain belongs to the O25:H4 serotype and identify/quantify the genes expressed. In parallel, proteomic maps of C999 were produced by two-dimensional gel electrophoresis (2DE) of whole-cell and fractionated extracts followed by matrix-assisted laser desorption/ionization time-of-flight mass spectrometry (MALDI-TOF/MS) of peptides (Vlaanderen et al., 2010). This allowed us to monitor how resistance mechanisms affect the proteomes of the membrane and cytoplasmic compartments.

## Materials and Methods

### Whole-Genome Sequencing

Total DNA was extracted from *E. coli* C999 using a silica-based automatic DNA extractor EasyMag (BioMérieux Inc., Durham, United States). A sequencing library was generated using the Nextera XT DNA library preparation kit (Illumina Inc., San Diego, CA, United States) and sequenced on a MiSeq (Illumina Inc., San Diego, CA, United States) using paired-end reads (2 × 150 bp), according to the manufacturer’s instructions. FastQC^1^ and Trimmomatic^2^ software tools were used for read quality analysis and improvement, respectively (D’Antonio et al., 2015; Williams et al., 2016). Genome assembly and annotation were done using SPAdes^3^ and RAST annotation^4^, respectively. Finally, putative antimicrobial resistance genes were predicted using Comprehensive Antibiotic Resistance Database (CARD^5^) (Jia et al., 2017). WGS raw reads were submitted to the European Nucleotide Archive under the accession numbers ERR3013427.

### RNA Library Preparation and Sequencing

Total RNA was extracted using the RNeasy Mini Kit (Qiagen, Venlo, Netherlands) with RNase-free DNase treatment on the column (Qiagen), followed by bacterial rRNA depletion using a Ribo-Zero rRNA Removal Kit (Illumina Inc., San Diego, CA, United States). The 2100 Bioanalyzer (Agilent, Santa Clara, CA, United States) was used to evaluate the concentration and quality of RNA samples pre- and post-depletion. For RNA-Seq analysis, a library was prepared with rRNA-depleted samples using the TruSeq Stranded mRNA LT Sample Prep Kit (Illumina). RNA was sequenced on a MiSeq using paired-end (2 × 75 bp) reads (Illumina), according to the manufacturer’s instructions.

### Transcriptomic Data Analysis

The quality of raw RNA-Seq data was evaluated using FastQC analysis. The sequence reads were then mapped against the obtained whole-genome sequence of strain C999 using the Bowtie2 algorithm^6^ (Version 2.1.0). The expression level of each transcript was calculated using the Cufflinks software^7^ (Version 2.1.1) by normalizing data as fragments per kilobase of coding sequence per million mapped reads (FPKM).

### Whole-Cell Protein Extraction

Cells were grown in brain heart infusion agar for 24 h and afterward cultivated in brain heart infusion broth (15 ml) for 4 h (Goncalves et al., 2014). Exponentially growing cells were then harvested by centrifugation (3 min, 10,000 g, 4°C) and resuspended in 4 ml of phosphate-buffered saline at room temperature, centrifuged again, then resuspended in 0.2 ml of 10% (w/v) sodium dodecyl sulfate (SDS), 12% (w/v) Tris (Celis and Gromov, 1999). Cells were disrupted by sonication with an ultrasonic homogenizer (Vibra-Cell^TM^ VCX130, Sonics & Materials Inc., Newtown, United States) in three 10 s bursts at 40% of full power, then cell debris was removed by centrifugation (14,000 g, 30 min, 4°C). The clear supernatant was collected then mixed with an equal volume of cold 20% (w/v) trichloroacetic acid (TCA; Merck, Darmstadt, Germany) in acetone (Sigma-Aldrich, St. Louis, MO, United States) and was kept at -20°C for 1 h. The precipitate was collected by centrifugation at 13,000 g for 30 min at 4°C. The precipitated protein was washed thrice with acetone to remove traces of TCA. Residual acetone was removed by air-drying. Protein pellets were solubilized in thiourea/urea lysis buffer. The resulting solutions were stored at -80°C for further analysis.

### Extracellular Protein Extraction

Extracellular proteins were prepared as previously described with some modifications (Nandakumar et al., 2006; Xia et al., 2008; Goncalves et al., 2014). Cells were removed from brain heart infusion broth culture by centrifugation at 5500 g for 10 min at 4°C. The clear supernatant was collected, passed through a 0.22 μm filter, mixed with an equal volume of cold 20% (w/v) TCA in acetone, and kept at -20°C for 1 h. The precipitate formed after centrifugation at 13,000 g for 30 min at 4°C was washed thrice with acetone to remove traces of TCA, and residual acetone was removed by air-drying. Dried protein pellets were solubilized in thiourea/urea lysis buffer [2 M thiourea, 7 M urea, 4% (w/v) CHAPS, 1% (w/v) dithiothreitol (DTT), 2% (v/v) carrier ampholytes (pH 3–10) and 10 mM Pefabloc^®^ proteinase inhibitor], then stored at -80°C for further analysis.

### Periplasmic and Cytoplasmic Protein Extraction

To extract periplasmic and cytoplasmic protein from bacterial cultures, the Epicentre Peripreps^TM^ Periplasting kit (Epicentre, WI, United States) was used with a few modifications to the kit protocol. The bacterial cell culture was centrifuged at 5500 g for 10 min and the supernatant discarded. The pellet was resuspended by pipetting in 2 ml of PeriPreps^TM^ Periplasting buffer (200 mM Tris-HCl pH 7.5, 20% sucrose, 1 mM EDTA, and 30 U/μl Ready-Lyse lysozyme) for each gram of cell pellet. The sample was incubated for 5 min at room temperature. Osmotic shock was induced by rapidly adding 3 ml of ice-cold water for each gram of original cell pellet, mixing the sample by inverting the centrifuge tubes. The sample was kept on ice for 10 min then centrifuged at 12,000 g for 2 min to separate the pellet (spheroplasts and intact cells) from the supernatant, the periplasmic fraction. Spheroplasts in the pellet were lysed by adding a detergent lysis buffer (10 mM KCl, 1 mM EDTA, and 0.1% deoxycholate) and the pellet was resuspended in 5 ml of PeriPreps lysis buffer for each gram of original cell pellet and incubated for 5 min at room temperature. The sample was sonicated with 2 s bursts at 40% of full power for a total of 1 min. Cell debris was removed by centrifugation at 12,000 g for 15 min at 4°C. The supernatant was removed and centrifuged as before. The supernatant recovered was the cytoplasmic fraction. Equal volumes of cold 20% (w/v) TCA in acetone were mixed with both periplasmic and cytoplasmic fractions that were then kept at -20°C Tris-HCl pH 7.5, 50 for 1 h. The precipitates collected after centrifugation at 13,000 g for 30 min at 4°C were washed thrice with acetone to remove traces of TCA. Residual acetone was removed by air-drying. Protein pellets were solubilized in thiourea/urea lysis buffer and stored at -80°C for further analysis.

### Membrane Protein Extraction

Membrane proteins were isolated by a previously described method with some modifications (Taddei et al., 2011). Bacterial cells were recovered from liquid culture by centrifugation at 10,000 g for 3 min at 4°C and the pellet was resuspended in phosphate-buffered saline pH 7.4 (Gorg et al., 2004). After a second similar centrifugation step, the pellet was resuspended in 25 ml of 10 mM Tris buffer pH 8.8 with 1 mM phenylmethylsulfonylfluoride (Sigma-Aldrich). Cells were disrupted with 3 cycles of 20 s bursts of sonication at 40% of full power and the cell debris was removed by centrifugation at 12,000 g for 2 min at room temperature. The supernatant was centrifuged at 49,500 g for 60 min at 4°C (in a 3–30KS centrifuge with rotor no.12158, Sigma GmbH, Osterode am Harz, Germany) and the pellet was treated with 1.67% N-lauroylsarcosine sodium salt (Sigma-Aldrich) for 20 min at room temperature. The membrane proteins were recovered by centrifugation at 23,000 g for 90 min at 4°C and the pellet was solubilized in thiourea/urea lysis buffer. Samples were stored at -80°C for further analysis.

### Protein Quantification

Protein concentration was determined using the 2-D Quant kit (GE Healthcare, Buckinghamshire, United Kingdom) following the manufacturer’s instructions. In this procedure proteins are quantitatively precipitated leaving other substances in solution. The precipitated proteins are then resuspended in a copper-containing solution with the unbound copper being measured with a colorimetric agent. Color density (absorbance at 480 nm) is thus inversely related to the protein concentration and accurately reflects the protein concentration of the sample.

### One-Dimensional and Two-Dimensional Electrophoresis

One-dimensional electrophoresis was done with SDS-polyacrylamide (SDS-PAGE) gels (*T* = 12.52%, *C* = 0.97%) in a HoeferTM SE 600 Ruby^®^ unit (GE Healthcare, Chicago, United States) as described by Laemmli (1970) with some modifications (Igrejas, 2000). Whole-cell protein extract (15 μg) was resuspended in an equal volume of buffer containing 0.5 M Tris HCl pH 8.0, glycerol, SDS and bromophenol blue. After protein separation at 30 mA, gels were stained for 24 h in Coomassie Brilliant Blue R-250 and washed in water overnight. Gels were then fixed in 6% TCA for 4 h and in 5% glycerol for 2 h (Gorg et al., 2009). Two-dimensional electrophoresis (2DE) was performed according to the principles of O’Farrell but with Immobiline^TM^ pH Gradient (IPG) technology (O’Farrell, 1975; Gorg et al., 2009). For the first dimension of isoelectric focusing, precast 13 cm IPG strips with linear gradients of pH 3–10 (GE Healthcare) were passively rehydrated for 12–16 h in a reswelling tray with 250 μl of rehydration buffer (8M urea, 1% CHAPS, 0.4% DTT, and 0.5% carrier ampholyte IPG buffer pH 3–10) at room temperature. IPG strips were covered with Drystrip Cover Fluid (Plus One, GE Healthcare). Lysis buffer [9.5 M urea, 1% (w/v) DTT, 2% (w/v) CHAPS, 2% (v/v) carrier ampholytes (pH 3–10), and 10 mM Pefabloc^®^ proteinase inhibitor] was added to *E. coli* protein extracts to achieve a concentration of 1 μg/μl of protein. Samples containing a total of 100 μg of protein were cup-loaded onto the rehydrated 13-cm IPG strips (Gorg et al., 2009). To optimize running conditions, isoelectric focusing replicate runs were performed according to Gorg et al. (2009) and the GE Healthcare protocol for 13 cm IPG strips pH 3–10 on an Ettan^TM^IPGPhor II^TM^ (GE Healthcare). The optimized 13 h run was as follows: sample proteins were focused at 500 V for 1 h, followed by a gradient up to 1000 V for 8 h, then a gradient up to 8000 V for 3 h, finally remaining at 8000 V for 1 h. Focused IPG strips were then stored at -80°C in plastic bags. Before running the second dimension of electrophoresis, strips were equilibrated twice for 15 min in equilibration buffer [6M urea, 30% (w/v) glycerol, 2% (w/v) SDS in 0.05M Tris-HCl buffer (pH 8.8) with bromophenol blue] with 1% DTT included in the first equilibration and 4% iodoacetamide in the second one. The equilibrated IPG strips were briefly rinsed with SDS electrophoresis buffer, blotted to remove any excessive buffer, and then loaded onto 12.52% polyacrylamide gels in a Hoefer^TM^ SE 600 Ruby^®^ unit (GE Healthcare). The SDS-PAGE technique previously reported by Laemmli (1970) was modified to increase the resolution with the proper insertion of the IPG strips in the stacking gel (Laemmli, 1970; Igrejas, 2000). SDS-PAGE was run at 440 V for 3 h. Gels were fixed in 40% methanol, 10% acetic acid for 1 h, then stained overnight in Coomassie Brilliant Blue G-250 (Gorg et al., 2009). Coomassie-stained gels were scanned on a flatbed scanner (UmaxPowerLook 1100, Freemont, CA, United States) and the digitized images were analyzed using Lab Scanner Image Master 5.0 software (GE Healthcare). Protein molecular weights were estimated by comparison with an internal calibration marker.

### Protein Identification by MALDI-TOF/MS

For each extraction method, gels were analyzed and compared with each other. Spots that were expressed in all gels were manually excised from the gels and analyzed using MALDI-TOF/MS. Gel pieces were rehydrated twice in 200 μl Milli-Q water and washed twice with 25 mM ammonium bicarbonate, 50% acetonitrile (ACN), once with 50 μl ACN, then dried in a SpeedVac (Thermo Fisher Scientific, Waltham, MA, United States). To digest the proteins, 15 μl of trypsin solution [0.02 μg/μl trypsin (Promega, Madison, WI, United States), 12.5 mM ammonium bicarbonate, 2% (v/v) can] was added to the dried gel pieces, which were then kept on ice for 1 h before adding 30 μl of 12.5 mM ammonium bicarbonate and incubating them overnight at 37°C. Tryptic peptides were extracted by adding 20 μl of 5% formic acid, 50% ACN and then 25 μl of 50% ACN, 0.1% trifluoroacetic acid followed by threefold lyophilization in a SpeedVac (Thermo Fisher Scientific). Tryptic peptides were resuspended in 10 μl of 0.3% formic acid. Samples were mixed (1:2, v/v) with 1 μl of a saturated matrix solution of 5 mg/ml α-cyano-4-hydroxycinnamic acid in 0.1% (v/v) trifluoroacetic acid, 50% (v/v) ACN, 8 mM ammonium phosphate). Aliquots of samples (0.5 μl) were spotted onto the MALDI sample target plate (384-spot ground-steel plate). Peptide mass spectra were obtained from a MALDI-TOF/MS Ultraflex mass spectrometer (Bruker Daltonics, Bremen, Germany) operating in positive ion reflectron-mode. Spectra were acquired in the m/z range of 600–3500 Da at a laser frequency of 50 Hz. For each spot analyzed, a data-dependent acquisition method was created to select the six most intense peaks, excluding those from the matrix, trypsin autolysis, or acrylamide, for subsequent MS/MS data acquisition. Mass spectra were internally calibrated with self-digested trypsin peaks (MH+: 842.5, 2211.42 Da) allowing a mass accuracy of better than 25 ppm. External calibration was performed with the [M + H]+ monoisotopic peaks of bradykinin 1–7 (*m*/*z* 757.3992), angiotensin II (*m*/*z* 1046.5418), angiotensin I (*m*/*z* 1296.6848), substance P (*m*/*z* 1758.9326), ACTH clip 1–17(*m*/*z* 2093.0862), ACTH18–39 (*m*/*z* 2465.1983), and somatostatin 28 (*m*/*z* 3147.4710).

### Bioinformatics Analysis for Proteomics

Spectra were processed and analyzed using the Global Protein Server Workstation (Applied Biosystems), which uses internal MASCOT software (v 2.1.04, Matrix Science, London, United Kingdom) to search for peptide mass fingerprints within MS/MS data. The Swiss-Prot non-redundant protein sequence database (Release 10 of October 2014, 546790 entries) and NCBI Reference Sequence Database (RefSeq release 68 of November 2014, 46968574 protein entries) were used to search *E. coli* protein sequences. The database search parameters were as follows: carbamidomethylation and propionamide of cysteine (+71 Da) and oxidation of methionine (+16 Da) as variable modifications, allowance for up to two missed tryptic cleavages, peptide mass tolerance of 50 ppm, and fragment ion mass tolerance of 0.3 Da. Positive identifications were accepted above 95% of confidence level. Protein identifications were considered as reliable when the MASCOT score was >70% calculated as –10 × log P, where P is the probability that the observed match is a random event. This is the lowest score indicated by the program as being significant (*P* < 0.05) below which proteins are likely to be incorrectly identified.

## Results and Discussion

### *E. coli* C999 Strain Profile

#### Genomics and Transcriptomics

ESBL-producing *E. coli* strain C999, implicated in a urinary infection of a Spanish patient was collected in 2007 and used in this study, thus characterized in relation to the phenotype and genotype of antimicrobial resistance and to molecular typing (Ruiz et al., 2012). This strain was resistant to ampicillin, amoxicillin/clavulanic acid, cefotaxime, ceftazidime, naladixic acid, ciprofloxacin, tobramycin, kanamycin, streptomycin, tetracycline, sulfamides and trimethoprim-sulfametoxazole, and carried the *bla*_CTX-M-15_, *bla*_OXA-1_, and *bla*_TEM-1b_ β-lactamase genes. Other resistance genes observed in strain C999 were *aac(6’)-Ib-cr* (ciprofloxacin resistance), *tet(A)* (tetracycline resistance) and *sul1* (sulfametoxazole resistance). The gene cassette array *dfrA17* + *aadA5* was observed in strain C999 and mutations were also found in genes encoding GyrA (Ser83Leu + Asp87Asn) and ParC proteins (Ser80Ile + Glu84Val) (Ruiz et al., 2002). C999 was classified in the phylogenetic group B2, mostly implicated in extraintestinal infections (Clermont et al., 2000), and it belongs to sequence type ST131, as previously detected (Ruiz et al., 2012). To better understand the nature of the C999 strain we produced a comprehensive survey of its genome, transcriptome and proteome. WGS allowed comprehensive characterization of the genetic makeup of the bacterial strain, including the identification of antibiotic resistance genes, consistent with the known pathological nature of this strain previously determined by Ruiz and colleagues (Ruiz et al., 2012). In fact, *in silico* prediction was applied to the WGS assay using SerotypeFinder 2.0, thus confirming the O25:H4 serotype which can lead us to acknowledge our strain as a member of the O25:H4-ST131 *E. coli* clonal group (Joensen et al., 2015). Supplementary Table S1 display all the identified genes related to antibiotic resistance such as *aac(6’)-Ib-cr*, *tet(A)*, *sul1*, *aadA5* gene cassette and genes related to toxin-antitoxin addiction systems of plasmids *pemK*, *ccdA/ccdB*, *vagC/vagD*, and *sok*, as well as β-lactamase genes *bla*_TEM-1_, *bla*_OXA-1_, and *bla*_CTX-M-15_. It is important to also highlight the presence of several stress response and oxidoreductase genes. This perspective of the C999 transcriptome gives an overview of all its cellular mechanisms (Supplementary Table S2).

#### Proteomics

**FIGURE 1:**
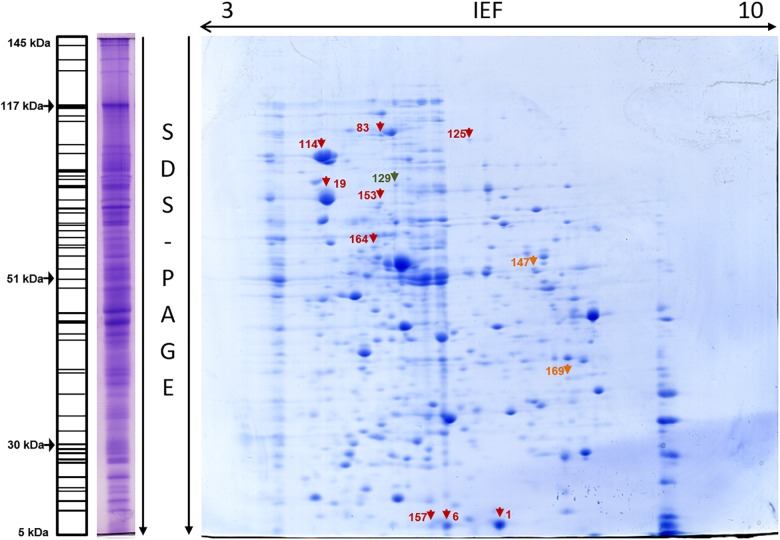
Whole-cell proteome of *E. coli* C999. One-dimensional SDS-PAGE gel profile stained with Coomassie R-250 (left) with schematic profile showing molecular weights. Two-dimensional gel stained with Coomassie G-250 with spots marked (right). Protein spot color key: dark red, stress response; orange, SOS response; dark green, antibiotic resistance.

**FIGURE 2:**
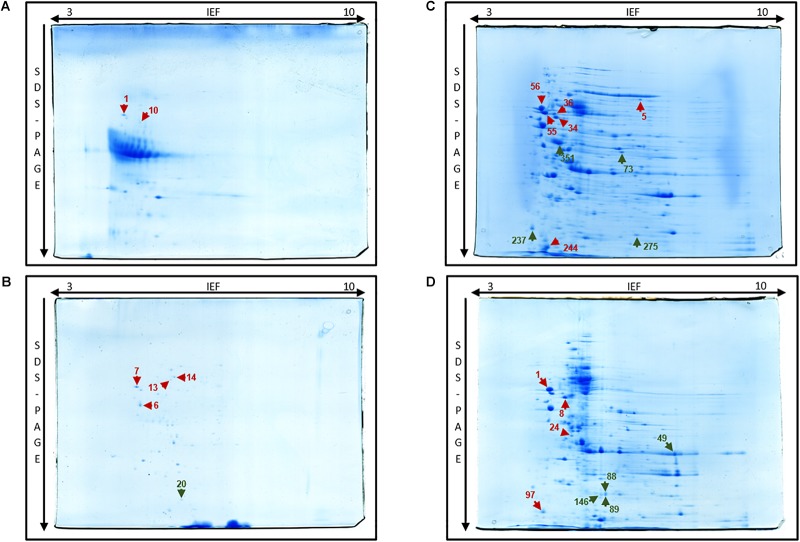
Two-dimensional gel of *E. coli* C999 of extracellular **(A)**, periplasmic **(B)**, membrane **(C),** and cytoplasmic **(D)** proteome fractions stained with Coomassie G-250 with spots marked (right). Protein spot color key: dark red, stress response; orange, SOS response; dark green, antibiotic resistance.

**FIGURE 3:**
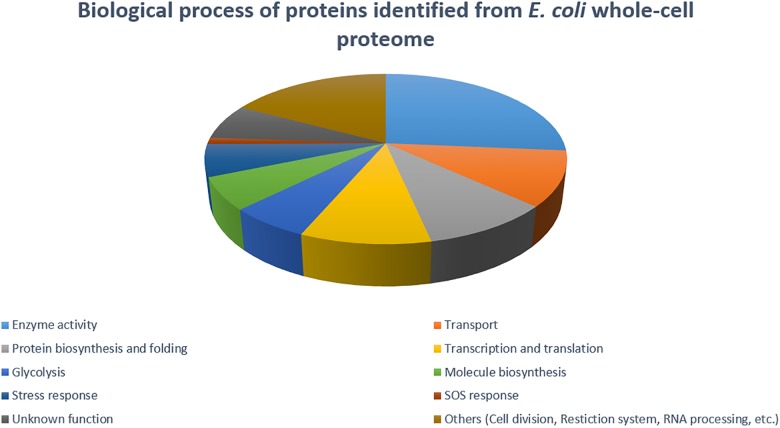
Distribution of *E. coli* strain C999 proteins according to their predicted function in biological processes.

**FIGURE 4:**
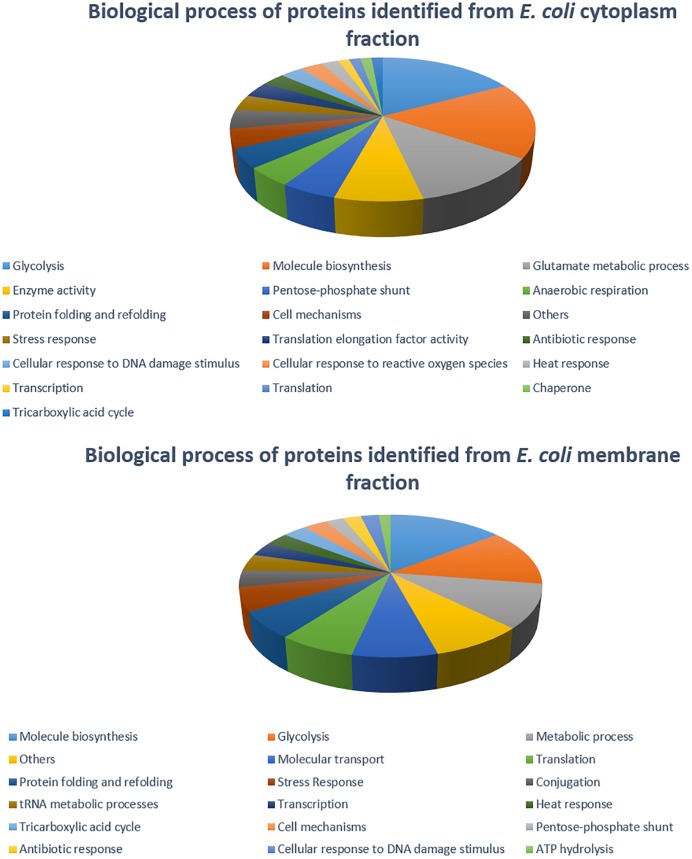
Distribution of *E. coli* strain C999 proteins from cytoplasm and membrane fractions according to their predicted function in biological processes.

**FIGURE 5:**
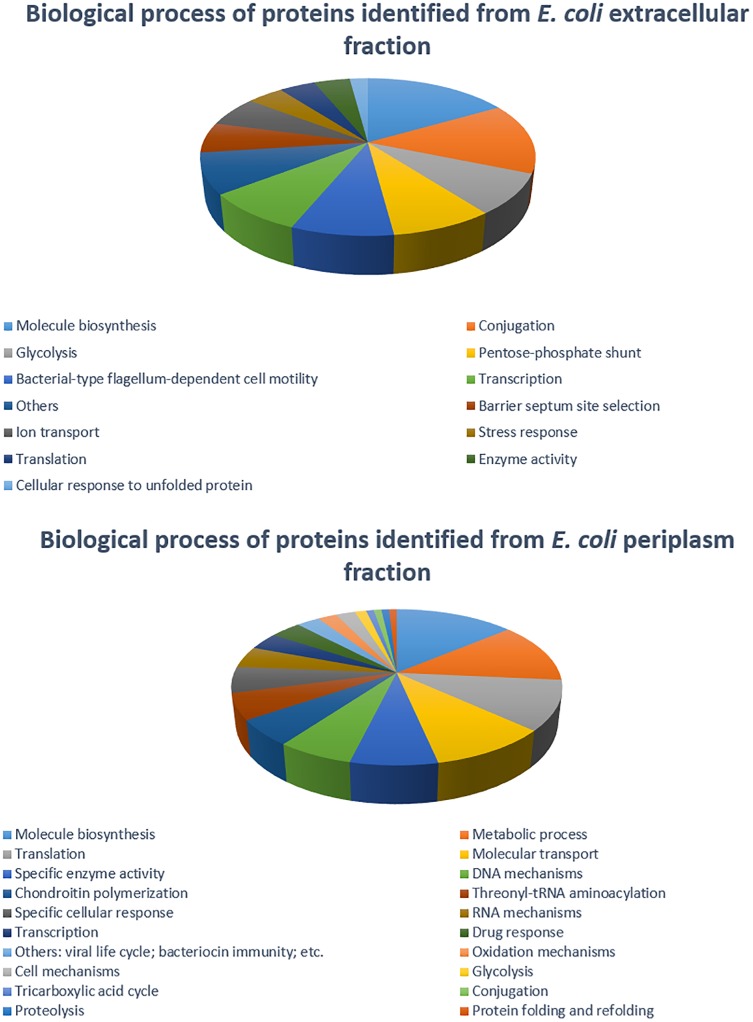
Distribution of *E. coli* strain C999 proteins from extracellular and periplasm fractions according to their predicted function in biological processes.

**FIGURE 6:**
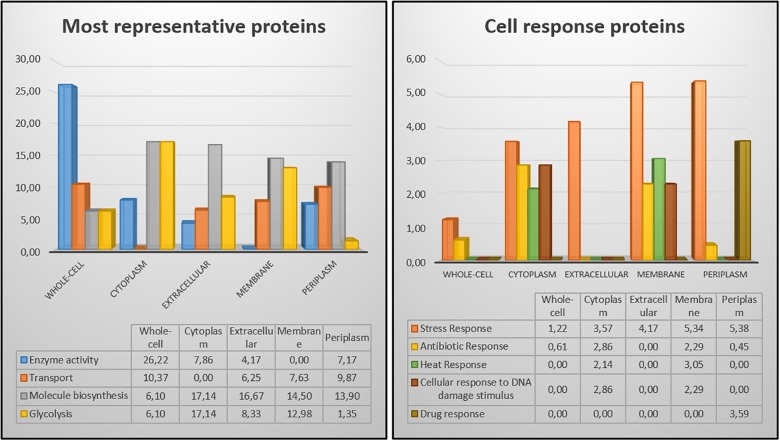
Comparison of protein distribution among the different cellular fractions and whole-cell extract.

The 2DE gels of the whole-cell proteome and four sub-proteomes of *E. coli* strain C999 were compared (Figure 1, 2). From all the gels, a total of 564 protein spots were collected for analysis using MALDI-TOF/MS and identified by correlating the output with bioinformatics databases^8^. A total of 602 different proteins were identified from 471 different spots, which corresponds to 83.51% of the total spots collected (Supplementary Tables S5–S9). The proteins identified were related to different functions within bacterial cell metabolism, the most frequent being enzyme activity, transport and molecule/protein biosynthesis, followed by the stress response, the SOS response and antibiotic resistance (Figure 3–5). Proteins related to glycolysis and molecule biosynthesis were indeed well represented in all proteomes (Figure 6). In fact, 282 different proteins were identified as involved in biological processes of regular cell functioning and 42 proteins were found to be related to stress response mechanisms, as has been previously described (Micevski and Dougan, 2013; Delmar et al., 2014).

### Comparison of RNA and Proteins Expressed in *E. coli* C999

With the use of RNA-Seq the abundance of all transcripts was quantified, thus allowing to compare the gene expression levels to the proteomic data (Supplementary Table S3; Han et al., 2015; Salipante et al., 2015). Supplementary Table S4 summarizes the relevant genes identified with their lengths and abundance in FPKMs, juxtaposed with the proteomic data obtained and corresponding protein score. Taking an overview of all the data obtained, it is interesting to see that among the top-100 most highly expressed genes only 25 corresponded to detected proteins, whereas only 80 detected proteins were among the top-500 expressed genes. In fact, gene *bla*_CTX-M-15_ was identified with an expression level of 355 FPKM being placed in the top-1000 although not being detected at the proteome level. The lack of correlation between mRNA and protein expression was already referred in previous studies, where different possibilities were advanced to explain this matter (Haider and Pal, 2013; Koussounadis et al., 2015; Liu et al., 2016). Considering the most highly expressed genes which did correlate well with the proteomic data in our survey, we can highlight the antibiotic resistance-related gene *bla*_TEM_ and also elongation factor *tufA*, as well as stress response genes *dps*, *clpB*, *dnaK*, and *groEL* (Supplementary Table S3). According to the genomic and transcript sequences, various expressed genes are related to multidrug resistance mechanisms. One example is the efflux pump AcrA-AcrB-TolC located in the intermembrane structure of Gram-negative bacteria, which ejects antibiotics and other compounds from the cell, thus playing an important part in the survival of pathogenic microorganisms (Supplementary Table S2; Tikhonova and Zgurskaya, 2004; Wang et al., 2009; Du et al., 2014). Adaptor protein (AcrA) and outer membrane channel (TolC) transcripts were both detected in RNA-Seq, and the AcrA homolog AcrE, the transcriptional repressor AcrR, and the potential AcrA-repressor AcrS were all expressed but at different levels (Supplementary Table S3). AcrE is very similar to AcrA and can substitute for AcrA function in multidrug transport, while AcrR can repress acrAB operon expression (Hirakawa et al., 2008; Hayashi et al., 2016). The *acrS* gene is upstream of *acrE*, and the protein binds to the same sequence on the AcrA promoter that is recognized by AcrR, thus potentially acting as an AcrA repressor negatively regulating kanamycin resistance (Hirakawa et al., 2008). As expected, the TolC protein was identified in the membrane sub-proteome, expressed at low levels (Supplementary Table S4). Outer membrane channel TolC is involved in various efflux and drug transportation systems like the tripartite systems EmrAB–TolC and MdtABC-TolC/MdtEF-TolC, and other resistance efflux systems that confer the capability to resist and expel a wide range of antibiotics, detergents and chemical solvents (Tanabe et al., 2009; Lennen et al., 2013; Anes et al., 2015). Genes *emrA*, *emrB*, *emrD*, *emrE*, *emrK*, and *emrR* were identified in our RNA-Seq survey at low expression levels (Supplementary Table 3, below 124 FPKMs), while genes *mdtA*, *mdtB*, *mdtC*, *mdtD*, *mdtE*, *mdtG*, *mdtI*, *mdtJ*, *mdtK*, *mdtL*, *mdtM*, *mdtN*, *mdtO*, and *mdtP* showed slightly higher expression levels (above 274 FPKMs). Except for TolC, the above efflux system components were not detected in the proteome. The lipid A-Ara4N pathway is involved in polymyxin resistance because Ara4N (4-amino-4-deoxy-L-arabinose) is added to phosphate groups of lipid A. Genes encoding lipid A-Ara4N pathway components are well represented among the RNA-Seq data with *arnC*, *arnE*, *arnF*, and *arnT* expressed between 64 and 45 FPKMs (Supplementary Tables S3, S4; Gatzeva-Topalova et al., 2005b). Even though the expression levels of the latter transcripts were similar, only bifunctional polymyxin resistance protein ArnA was expressing at low level in the membrane sub-proteome. Polymyxin resistance is also triggered by the up-regulation of operon *arnBCADTEF*, which is directly involved in the activation of the two-component system PmrA/PmrB that is represented in the RNA-Seq readout (Olaitan et al., 2014). Genes *pmrA*, *pmrB*, *pmrD*, *pmrG*, *pmrJ*, *pmrL*, and *pmrM* were expressed at under 178 FPKMs whilst the respective peptides they encode were not detected. The presence of these resistance mechanisms in clinical isolates with increased virulence raises concern for the spontaneous polymyxin resistance phenomena thus indicating why such bacteria reveal high pathogenic potential. Some other abundant transcripts did not have corresponding proteins in the proteomic data, which may be due to a number of factors like post-translational mechanisms of regulation and differential protein stability that can be influenced by a protein’s location and/or interaction with other proteins, or even due to limitations within the proteomic techniques (Yoon et al., 2003; Hack, 2004; Kumar et al., 2016). In fact, the correlation between transcript and protein levels may vary according to specific patterns (Yoon et al., 2003). Immobilized pH gradient 2DE is widely used for protein separation and identification but have shown some limitations in resolving highly charged, long chain and insoluble proteins (Hack, 2004). Such proteins may therefore remain undetected with 2DE and MS techniques or displaying levels of expression below the define threshold, even when the corresponding genes and transcripts are identified through WGS and RNA-Seq, respectively. This reinforces the need to compare proteomic and transcriptomic results in order to fully characterize a given bacterial strain.

### Proteins Related to Bacterial Resistance Mechanisms

#### Antibiotic Resistance

Following several reports of the identification and expression of antibiotic resistance genes around the world (Lavigne et al., 2007; Mitsou et al., 2010; Barguigua et al., 2011; Kim et al., 2011), the dynamics of the proteome and the mechanism(s) of bacterial antibiotic resistance need to be considered in the context of the spread of bacterial pathogens (Cash, 2011). Elongation factor Tu, encoded by *tufA*, was identified in spot 46 [molecular weight (MW) 41636, isoelectric point (pI) 5.00] of the cytoplasm fraction (Figure 2). Present in most enterobacterial genomes, TufA is responsible for binding and transporting an appropriate codon-specified aminoacyl-tRNA to the ribosome aminoacyl site, and it also influences the assembly and stability of cytoskeletal polymers and is implicated in protein folding and protection from stress (Caldas et al., 1998; Isabel et al., 2008). Levels of TufA protein and transcripts were found to be elevated in C999 which is relevant and consistent with previous reports of *tufA* upregulated expression in the presence of antibacterial peptide polymyxin B, regulated by the *pmrA/pmrB* two-component system (Supplementary Table S2; Isabel et al., 2008; Ribeiro et al., 2013). Another one of the most expressed genes is β-lactamase TEM-1, which was present in the periplasmic sub-proteome [MW 31666, pI 5.60] (see Figure 6 and Supplementary Tables S3, S9). Plasmid-encoded β-lactamases are among the most critical acquired resistance determinants emerging in members of *Enterobacteriaceae* such as *E. coli* (Hooff et al., 2012). The detection of this protein is noteworthy, even though the level of expression was low and poorly correlated with the mRNA levels determined by RNA-Seq. It is also relevant to note that not any other β-lactamase protein was found expressing, unlike the corresponding gene *bla*_CTX-M-15_ frequently found carried in ST131 *E. coli* clones accompanied by quinolone resistance gene *aac(6’)-Ib-cr* (Chong et al., 2018). Similar uncorrelated levels of RNA and protein expression were found for outer membrane protein TolC [spot 351], a component of the efflux pump system which rids the cell of antibiotics like tetracycline (to which *E. coli* C999 is resistant) and chloramphenicol (Weatherspoon-Griffin et al., 2014). The antibiotic resistance related FabI protein, an enoyl-[acyl-carrier-protein] reductase [NADH], was detected in spot 129 [MW 28074, pI 5.58] of the whole-cell proteome and in spots 88, 89, and 146 of the cytoplasmic sub-proteome. The detection of FabI in various spots, although it occurs at low levels of protein expression, suggests the existence of post-translational modification affecting protein stability (Maier et al., 2009). FabI is a homo-tetrameric enzyme responsible for the catalysis of the last reductive step of fatty acid biosynthesis, and it is a critical target for antibacterials commonly used mediating resistance to *E. coli* enterohemorrhagic serotypes (see Supplementary Table S1). In *Staphylococcus aureus*, FabI is known to be inhibited by triclosan, a broad-spectrum antibacterial additive and hexachlorophene, which results in FabI being less effective toward Gram-negative bacteria (Heath et al., 2000; Schiebel et al., 2014). The previously cited bifunctional polymyxin resistance protein ArnA [spot 275] is a pathway-specific enzyme possessing a C-terminal domain which catalyzes the NAD+-dependent oxidative decarboxylation of UDP-GlcA to UDP-β-(L-*threo*pentapyranosyl-4″-ulose) (UDP-4-keto-pentose) (Gatzeva-Topalova et al., 2005b). This pathway is implicated in the pathophysiological effects associated with Gram-negative bacterial infections (Gatzeva-Topalova et al., 2005a). Aminoglycoside 3′-phosphotransferase AphA [spot 73] is reported to be involved in resistance to kanamycin and structurally-related aminoglycosides like tobramycin (Shi et al., 2013). Knowing that kanamycin and tobramycin were tested when phenotyping C999, the detection of the AphA protein confirms that the corresponding resistance is expressed at the proteome level. It is interesting that while ArnA transcript levels were consistently low, the AphA transcripts were not detected, which suggests that some regulatory mechanisms remain to be discovered. In the periplasmic fraction was detected the presence of two hits of Ferrous iron transport protein A, a known virulent factor, but under a very low protein score so that its identification is not validated (Supplementary Table S9).

#### Stress Response, Oxidoreductase, and SOS Response

The environmental stress response is a defense mechanism found in all bacteria in which many different factors regulate gene and protein expression according to the specific stress encountered (Calabrese et al., 2012). The analysis of both the transcriptome and proteomes of C999 revealed the presence of several genes related to stress response mechanisms that increase the survival rate of bacteria, a relevant factor when considering non-commensal bacteria that will therefore endure. Stress response associated Dps (DNA protection during starvation) protein [spots 1, 6, and 157; MW 18684 and pI 5.70], another factor contributing to the bacteria’s survival, was highly expressed in both the whole-cell proteome and transcriptome (Figure 1, 2 and Supplementary Table S2). Very similar to ferritins, Dps has a compact and stable shell-like structure assembled from twelve identical subunits, with the lysine-rich N-termini of each monomer conferring flexibility. When present in stationary phase cells, Dps can bind DNA to form a highly stable DNA-Dps complex, which protects bacteria from oxidative stress or nutritional deprivation caused by harmful environmental stimuli (Stephani et al., 2003; Calhoun and Kwon, 2011). The highly stable protein conformation is also known to influence *E. coli* attachment to abiotic surfaces (Goulter-Thorsen et al., 2011). Expression of chaperone proteins ClpB [MW 95697, pI 5.37], DnaK (HSP70) [MW 69130, pI 4.83], and 60 kDa chaperonin GroEL1 [MW 57464, pI 4.85] is associated with the stress response. DnaK (HSP70) is an ATP-dependent molecular chaperone operating in thermal resistance in bacteria (Miot et al., 2011). In conjunction with ClpB, the DnaK/HSP70 chaperone system, is able to dissolve protein aggregates to protect bacterial cells from the effects of protein inactivation and aggregation caused by great heat stress (Doyle et al., 2007). ClpB is an ATP-dependent molecular chaperone from the AAA+ ATPase superfamily essential for bacterial thermotolerance that was found in the C999 proteomes (see Supplementary Tables S5, S6, S9; del Castillo et al., 2010; Miot et al., 2011). Another major *E. coli* chaperone, GroEL1, was found in whole-cell [spot 19] and also in the cytoplasm [spot 3], periplasm [spot 6] and membrane fraction [spots 18, 19, and 20] (Richter et al., 2010). GroEL belongs to the HSP60 class and plays an important role in protein folding and heat stress resistance. In fact, all three types of chaperones have similar biochemical structures and are involved in protecting cells by resisting heat stress at different stages of the bacterial chemical response (Kyratsous and Panagiotidis, 2012). In terms of oxidative stress defense, oxidoreductase function related proteins SodA [MW 23083, pI 6.44], AhpC [MW 20862, pI 5.03], and thiol peroxidase protein (Tpx) [MW 17995, pI 4.75] were identified in the C999 whole-cell proteome and AhpC was also identified in the C999 membrane fraction (see Figure 1 and Supplementary Tables S5, S8). Superoxide dismutase (SodA) removes superoxide leading to the generation of hydrogen peroxide (H_2_O_2_) which is then removed by catalases like KatE and peroxidases like AhpC, the latter being a very extensively studied bacterial peroxiredoxin system (Jung and Kim, 2003; Seib et al., 2006; Dubbs and Mongkolsuk, 2007). Tpx is involved in the formation of biofilms alongside superoxide dismutase (SodC) in Shiga toxin *E. coli* O157:H7 where these periplasmic oxidative defense proteins are more highly expressed under biofilm-inducing conditions (Kim et al., 2006). Peroxidases Tpx and AhpC were also found to be expressed in *Salmonella enterica* where a *tpx* mutant is more susceptible to exogenous H_2_O_2_ and is less able to degrade it than the wild type. Tpx therefore contributes to the defense system of this pathogen enabling it to survive oxidative stress (Horst et al., 2010). Another bacterial stress response mechanism involves RNA polymerase sigma factor RpoH, previously described as the main regulator of the heat stress response. RpoH is induced by protein unfolding and cytoplasmic stress in response to heat, DNA damage or antibiotic exposure (see Supplementary Table S8; Narberhaus and Balsiger, 2003; Foster, 2007). In our survey, RpoH mRNA was expressed at a high level, but protein expression was low, which might be expected as most heat induced mechanisms are post-trancriptional (Narberhaus and Balsiger, 2003). Also related to the general stress response is the two-component system connector protein sensor-associating-factor A (SafA), a 65-amino-acid membrane protein in whole cells [spots 125 and 153] and in the periplasm [spot 13] that is involved in the acid response network of two-component signal transduction systems. In *E. coli* there are 14 gene products and at least 15 regulators implicated in acid response (AR) biochemistry, where GadE is the main activator protein of resistance genes like gadA and gadE. Regulation of GadE in turn involves several regulators like EvgA and PhoP (Masuda and Church, 2003). EvgS/EvgA is the major system for acid resistance in exponentially-proliferating cells, inducing SafA and thus interacting and activating another connected regulating system, the PhoQ/PhoP system (Eguchi et al., 2011). Also relevant are the chaperone-related curved DNA-binding protein and the Mdh oxidoreductase identified in whole-cell [spot 76], cytoplasm [spot 73], and membrane [spot 305] fractions, and ATP-dependent protease ATP-ase subunit HslU, characteristic of *E. coli* O139:H28 (enterohemorrhagic strain E24377A), found in the whole-cell [spot 164] and cytoplasm [spot 24] (Marzan and Shimizu, 2011). SOS response components figured among our results. The LexA repressor [spot 147; MW 22344, pI 9.64], one of the main proteins regulating the SOS response, was expressed at a low level even though its mRNA levels were high (Figure 3 and Supplementary Tables S2, S5). LexA represses the transcription of several genes involved in DNA damage repair to a basal level when a bacterial cell is exposed to UV or to widely used antibiotics, like β-lactams, fluoroquinolones and trimethoprim (Guerin et al., 2011; Yaguchi et al., 2011). Genes *lexA*-regulated have been shown to exhibit phenotypic heterogeneity with different levels of expression detected in different cell subpopulations. The heterogeneous expression is related to differential binding affinity of LexA to SOS boxes when DNA is damaged by external factors invoking the SOS response (Kamensek et al., 2010). On the subject of DNA UV damage, DNA replication and repair protein RecF [spot 169; MW 40717, pI 6.78] was also found. The functional *recF* gene is implicated in several forms of replication such as stable DNA replication and linear plasmid multimer replication, as well as in the recovery of replication in UV-irradiated *E. coli* cells. The RecF protein binds preferentially to single-stranded or linear DNA that arises during DNA metabolism such as replication and normal SOS induction, and repairs DNA breaks and gaps resulting from UV or other stresses. Cells lacking RecF pathway are thus hypersensitive to UV-induced damage (Handa et al., 2009; Ona et al., 2009).

## Conclusion

In order to find a solution to the concern multidrug-resistance in humans it is vital that researchers possess precise knowledge of the gene and protein expression of the clinical bacterial strains and whether they are related to pandemic strains such as O25:H4-ST131, allowing to understand the dynamic framework surrounding the expansion and endurance of such organisms. In our study, we followed a previous genomic profile of clinical strain *E. coli* C999 revealing characteristics of the extraintestinal pathogenic CTX-M-15 producing *E. coli* clonal group O25:H4-ST131 and exhibiting fluoroquinolone resistance as other plasmid-mediated resistances. Through transcriptomics tools we were able to confirm our strain to be O25:H4-ST131 and also identify several genes related to antibiotic resistance and survival-related processes like stress and SOS response. Proteomics allowed the identification and quantification of several proteins regarding also antibiotic resistance and stress response, within some degree of correlation to the RNA expression. While the proteomics data is very valuable, transcriptomics using RNA-Seq provide precise transcript quantification so mRNA and protein levels can be compared. However, the lack of correlation between mRNA and protein expression (or the difficulty in detecting it) indicates there is much to discover about cellular mechanisms of gene regulation that could advance our understanding of antibiotic resistance. It will be necessary to investigate such relationships, particularly in terms of specific stimuli, by increasing sampling frequency in a metaomics approach, for example. In summary, omics-based studies of the metabolic pathways of antibiotic resistance should continue to be done if answers and sustainable solutions are to be found.

## Author Contributions

LP carried out laboratory work, data analysis, and drafted the manuscript. CT, PP, CG, JN-M, and GI implemented data analyses and helped to draft the manuscript. GI, PP, and CG conceived the study and revised the manuscript. VB, JG, CS, and GI helped interpret compiled data. HS, JP, LV, and GI provided facilities and helped implementing the laboratory work. CS and LV performed WGS and RNAseq wet-lab procedures. VB and JG performed nucleic acids extraction/depletion for WGS and RNAseq, and associated bioinformatics analyses. All the authors reviewed and contributed to the manuscript, approving its submission.

## Conflict of Interest Statement

The authors declare that the research was conducted in the absence of any commercial or financial relationships that could be construed as a potential conflict of interest.

## Footnotes

1 http://www.bioinformatics.babraham.ac.uk/projects/fastqc/

2 http://www.usadellab.org/cms/?page=trimmomatic

3 http://bioinf.spbau.ru/spades

4 http://rast.nmpdr.org/

5 https://card.mcmaster.ca/

6 http://bowtie-bio.sourceforge.net/bowtie2/index.shtml

7 http://cufflinks.cbcb.umd.edu/

8 http://www.ncbi.nlm.nih.gov/

## Supplementary Material

TABLE S1 Antibiotic resistance genes of ESBL-producing *E. coli* isolate C999 identified by whole-genome sequencing and prediction using CARD software.

TABLE S2 Summary of the metabolic pathways activated according to the total RNA sequencing of *E. coli* isolate C999.

TABLE S3 Full integration of proteomic and transcriptomic data.

TABLE S4 Integration of proteomic data of interest with corresponding transcriptomic data.

TABLE S5 Identification of protein spots from 2DE gels of whole-cell extracts of ESBL-producing *E. coli* isolate C999 based on MALDI-TOF/MS sequencing results.

TABLE S6 Identification of protein spots from 2DE gels of cytoplasmic extracts of ESBL-producing *E. coli* isolate C999 based on MALDI-TOF/MS sequencing results.

TABLE S7 Identification of protein spots from 2DE gels of extracellular extracts of ESBL-producing *E. coli* isolate C999 based on MALDI-TOF/MS sequencing results.

TABLE S8 Identification of protein spots from 2DE gels of membrane extracts of ESBL-producing *E. coli* isolate C999 based on MALDI-TOF/MS sequencing results.

TABLE S9 Identification of protein spots from 2DE gels of periplasm extracts of ESBL-producing *E. coli* isolate C999 based on MALDI-TOF/MS sequencing results.

## Abbreviations

2DE: two-dimensional gel electrophoresis
ACN: acetonitrile
DTT: dithiothreitol
ESBL: extended-spectrum β-lactamase producing
FPKM: fragments per kilo base per million mapped reads
IPG: ImmobilineTM pH Gradient
MALDI-TOF/MS: matrix-assisted laser desorption/ionization time-of-flight mass spectrometry
PAGE: polyacrylamide gel
RNA-Seq: RNA sequencing
SDS: sodium dodecyl sulfate
TCA: trichloroacetic acid
WGS: whole genome sequencing
