# Supplementary material for: Multiomics Assessment of Gene Expression in a Clinical Strain of CTX-M-15-Producing ST131 Escherichia coli
Source: Front Microbiol. 2019 May 3;10:831. doi: 10.3389/fmicb.2019.00831 (PMC6509150; doi:10.3389/fmicb.2019.00831)
Supplement: TABLE S2 — Summary of the metabolic pathways activated according to the total RNA sequencing of E. coli isolate C999. [file Table_2.DOCX]

**Supplementary Table S2.** Summary of the metabolic pathways activated according to the total RNA sequencing of E. coli isolate C999.

| **Category** | **Subsystem** | | | |
| --- | --- | --- | --- | --- |
| Cofactors, Vitamins, Prosthetic Groups, Pigments | Biotin synthesis cluster | Molybdenum cofactor biosynthesis | Ubiquinone Biosynthesis | Coenzyme A Biosynthesis cluster |
|  | Biotin biosynthesis | Folate Biosynthesis | Cobalamin synthesis | Coenzyme A Biosynthesis |
|  | Thiamin biosynthesis | 5-FCL-like protein | Heme and Siroheme Biosynthesis | Riboflavin, FMN and FAD metabolism |
|  | Menaquinone and Phylloquinone Biosynthesis | Lipoic acid metabolism | Coenzyme B12 biosynthesis | Pyridoxin (Vitamin B6) Biosynthesis |
|  | Riboflavin, FMN and FAD metabolism in plants | NAD and NADP cofactor biosynthesis global | riboflavin to FAD | Folate biosynthesis cluster |
|  | Flavodoxin |  |  |  |
| Cell Wall and Capsule | dTDP-rhamnose synthesis | Rhamnose containing glycans | Capsular Polysaccharide (CPS) of Campylobacter | Lipid A modifications |
|  | Capsular heptose biosynthesis | Sialic Acid Metabolism | Inner membrane protein YhjD and conserved cluster involved in LPS biosynthesis | Peptidoglycan lipid II flippase |
|  | YjbEFGH Locus Involved in Exopolysaccharide Production | Sialic Acid Metabolism | KDO2-Lipid A biosynthesis | Lipopolysaccharide assembly |
|  | Capsular Polysaccharides Biosynthesis and Assembly | Colanic acid biosynthesis | LOS core oligosaccharide biosynthesis | Lipid A-Ara4N pathway ( Polymyxin resistance ) |
|  | Lipoprotein sorting system | Recycling of Peptidoglycan Amino Acids | UDP-N-acetylmuramate from Fructose-6-phosphate Biosynthesis | Peptidoglycan Crosslinking of Peptide Stems |
|  | Murein Hydrolases | Peptidoglycan Biosynthesis | YjeE |  |
| Virulence, Disease and Defense | Accessory colonization factor | Colicin V and Bacteriocin Production Cluster | Copper homeostasis: copper tolerance | Mycobacterium virulence operon involved in DNA transcription |
|  | Mediator of hyperadherence YidE in Enterobacteria and its conserved region | The mdtABCD multidrug resistance cluster | Beta-lactamase | Mycobacterium virulence operon possibly involved in quinolinate biosynthesis |
|  | Tolerance to colicin E2 | Lysozyme inhibitors | Resistance to chromium compounds | Mycobacterium virulence operon involved in protein synthesis (LSU ribosomal proteins) |
|  | Cobalt-zinc-cadmium resistance | Multiple Antibiotic Resistance MAR locus | Multidrug Resistance Efflux Pumps | Mycobacterium virulence operon involved in protein synthesis (SSU ribosomal proteins) |
|  | Adaptation to d-cysteine | Copper homeostasis | Zinc resistance | Aminoglycoside adenylyltransferases |
|  | Multidrug Resistance, Tripartite Systems Found in Gram Negative Bacteria | Mercury resistance operon | Mercuric reductase | Resistance to fluoroquinolones |
| Potassium metabolism | Potassium homeostasis | Glutathione-regulated potassium-efflux system and associated functions | Hyperosmotic potassium uptake |  |
| Miscellaneous | YrdC-YciO-Sua5 protein family | Aromatic dioxygenase mess | Single-Rhodanese-domain proteins | Broadly distributed proteins not in subsystems |
|  | Scaffold proteins for [4Fe-4S] cluster assembly (MRP family) | Dioxygenases (EC 1.13.11.-) | Phosphoglycerate mutase protein family | Muconate lactonizing enzyme family |
|  | DedA family of inner membrane proteins |  |  |  |
| Phages, Prophages, Transposable elements, Plasmids | Phage replication | Phage tail proteins 2 | Phage lysis modules | Integrons |
|  | Phage packaging machinery | Phage capsid proteins | Phage DNA synthesis | Phage tail proteins |
|  | Phage neck proteins | Phage tail fiber proteins | IbrA and IbrB: co-activators of prophage gene expression |  |
| Membrane Transport | General Secretion Pathway | ABC transporter oligopeptide (TC 3.A.1.5.1) | ABC transporter alkylphosphonate (TC 3.A.1.9.1) | Type 1 pili (mannose-sensitive fimbriae, gamma-fimbriae) |
|  | CBSS-562.2.peg.633 | ABC transporter branched-chain amino acid (TC 3.A.1.4.1) | ATP-dependent efflux pump transporter Ybh | The usher protein HtrE fimbrial cluster |
|  | Peptide ABC transport system Sap | ABC transporter dipeptide (TC 3.A.1.5.2) | Colonization factor antigen I fimbriae | The fimbrial Stf cluster |
|  | Bacterial signal recognition particle (SRP) | Copper Transport System | Ton and Tol transport systems | 2,3-diketo-L-gulonate TRAP transporter |
|  | Twin-arginine translocation system | Proton-dependent Peptide Transporters | TRAP Transporter unknown substrate 8 | Type VI secretion systems |
|  | Magnesium transport | NhaA, NhaD and Sodium-dependent phosphate transporters | Choline Transport | IncF Conjugal Transfer System |
|  | Transport of Nickel and Cobalt | Curli production | ECF class transporters | Conjugative transfer |
|  | Tricarboxylate transport system | Type IV pilus | TRAP Transporter collection |  |
| Iron acquisition and metabolism | Siderophore Enterobactin | Heme, hemin uptake and utilization systems in GramNegatives | Siderophore Aerobactin | Encapsulating protein for DyP-type peroxidase and ferritin-like protein oligomers |
|  | Siderophore assembly kit | Hemin transport system | Heme, hemin uptake and utilization systems in GramPositives | Iron transport system including ABC transporter |
|  | Ferrous iron transporter EfeUOB, low-pH-induced |  |  |  |
| RNA Metabolism | RNA pseudouridine syntheses | mnm5U34 biosynthesis bacteria | RNA methylation | tRNAmodification position 34 |
|  | Possible RNA modification and stress response cluster | Ribonuclease H | 16S rRNA modification within P site of ribosome | tRNAmodification position 34 |
|  | Methylthiotransferases | Polyadenylation bacterial | tRNA modification Bacteria | RNA 3'-terminal phosphate cyclase |
|  | RNA processing and degradation, bacterial | tRNA nucleotidyltransferase | tRNA modification Archaea | ATP-dependent RNA helicases, bacterial |
|  | tRNA processing | Rrf2 family transcriptional regulators | Queuosine-Archaeosine Biosynthesis | Transcription factors bacterial |
|  | Transcription initiation, bacterial sigma factors | Group II intron-associated genes | RNA polymerase bacterial |  |
| Nucleosides and Nucleotides | pyrimidine conversions | GMP synthase | Ribonucleotide reduction | Adenosyl nucleosidases |
|  | Pyrimidine utilization | Purine conversions | Pseudouridine catabolism | Adenosyl nucleosidases |
|  | De Novo Pyrimidine Synthesis | De Novo Purine Biosynthesis | Hydantoin metabolism | Nucleoside triphosphate pyrophosphohydrolase MazG |
|  | Xanthine Metabolism in Bacteria | Xanthine dehydrogenase subunits | Nudix proteins (nucleoside triphosphate hydrolases) | Housecleaning nucleoside triphosphate pyrophosphatases |
|  | Purine nucleotide synthesis regulator | Xanthosine utilization (xap region) | Purine Utilization | dNTP triphosphohydrolase protein family |
| Protein Metabolism | GroEL GroES | Peptidyl-prolyl cis-trans isomerase | Ribosome SSU bacterial | tRNA aminoacylation, Cys |
|  | Protein chaperones | Selenoprotein O | tRNA aminoacylation, Gly | Translation termination factors bacterial |
|  | Periplasmic disulfide interchange | Selenocysteine metabolism | tRNA aminoacylation, His | Translation elongation factor G family |
|  | tRNA aminoacylation, Val | tRNA aminoacylation, Ala | Universal GTPases | tRNA aminoacylation, Glu and Gln |
|  | tRNA aminoacylation, Met | Ribosome activity modulation | tRNA aminoacylation, Asp and Asn | tRNA aminoacylation, Glu and Gln |
|  | tRNA aminoacylation, Met | tRNA aminoacylation, Trp | Translation elongation factors bacterial | tRNA aminoacylation, Ser |
|  | tRNA aminoacylation, Ile | Ribosome biogenesis bacterial | tRNAs | tRNA aminoacylation, Tyr |
|  | tRNA aminoacylation, Arg | Ribosome LSU bacterial | tRNA aminoacylation, Lys | tRNA aminoacylation, Leu |
|  | Translation initiation factors bacterial | Programmed frameshift | tRNA aminoacylation, Thr | Glycyl-tRNA synthetase |
|  | tRNA aminoacylation, Pro | tRNA aminoacylation, Phe | Protein degradation | Proteasome bacterial |
|  | tRNA aminoacylation, Pro | Translation elongation factor P lysylation | Aminopeptidases (EC 3.4.11.-) | Omega peptidases (EC 3.4.19.-) |
|  | Lipoprotein Biosynthesis | Protein-L-isoaspartate O-methyltransferase | Metallocarboxypeptidases (EC 3.4.17.-) | Proteolysis in bacteria, ATP-dependent |
|  | Signal peptidase | Ribosomal protein S12p Asp methylthiotransferase | Putative TldE-TldD proteolytic complex | Peptide methionine sulfoxide reductase |
|  | Protein deglycation | Ribosomal protein S5p acylation | Dipeptidases (EC 3.4.13.-) |  |
| Cell Division and Cell Cycle | MukBEF Chromosome Condensation | Bacterial Cytoskeleton | YgjD and YeaZ | Intracellular septation in Enterobacteria |
|  | Macromolecular synthesis operon |  |  |  |
| Motility and Chemotaxis | Bacterial Chemotaxis | Flagellum | Flagellum in Campylobacter | Flagellar motility |
| Regulation and Cell signaling | Rcs phosphorelay signal transduction pathway | CytR regulation | Stringent Response, (p)ppGpp metabolism | BarA-UvrY(SirA) two-component regulatory system |
|  | cAMP signaling in bacteria | Orphan regulatory proteins | Zinc regulated enzymes | A conserved operon linked to TyrR and possibly involved in virulence |
|  | LysR-family proteins in Salmonella enterica Typhimurium | LysR-family proteins in *Escherichia coli* | Biofilm Adhesin Biosynthesis | MazEF toxin-antitoxing (programmed cell death) system |
|  | Murein hydrolase regulation and cell death | Toxin-antitoxin replicon stabilization systems |  |  |
| Secondary Metabolism | Cinnamic Acid Degradation | Auxin biosynthesis | Alkaloid biosynthesis from L-lysine |  |
| DNA Metabolism | Uracil-DNA glycosylase | RecA and RecX | DNA repair, bacterial DinG and relatives | DNA repair, bacterial RecFOR pathway |
|  | DNA repair, bacterial MutL-MutS system | DNA repair system including RecA, MutS and a hypothetical protein | 2-phosphoglycolate salvage | Restriction-Modification System |
|  | DNA repair, UvrABC system | DNA repair, bacterial | DNA repair, bacterial UvrD and related helicases | YcfH |
|  | DNA repair, bacterial photolyase | DNA repair, bacterial UmuCD system | DNA Repair Base Excision | DNA structural proteins, bacterial |
|  | Type I Restriction-Modification | DNA topoisomerases, Type I, ATP-independent | RuvABC plus a hypothetical | DNA topoisomerases, Type II, ATP-dependent |
|  | DNA processing cluster |  |  |  |
| Fatty Acids, Lipids, and Isoprenoids | Cardiolipin synthesis | Isoprenoinds for Quinones | Fatty Acid Biosynthesis FASII | Nonmevalonate Branch of Isoprenoid Biosynthesis |
|  | Glycerolipid and Glycerophospholipid Metabolism in Bacteria | Isoprenoid Biosynthesis | Carnitine Metabolism in Microorganisms | Isoprenoid Biosynthesis: Interconversions |
|  | Triacylglycerol metabolism | Polyprenyl Diphosphate Biosynthesis | Fatty acid metabolism cluster | Polyhydroxybutyrate metabolism |
|  | Acyl-CoA thioesterase II |  |  |  |
| Nitrogen Metabolism | Allantoin Utilization | Ammonia assimilation | Nitrosative stress | Denitrifying reductase gene clusters |
|  | Nitrate and nitrite ammonification |  |  |  |
| Dormancy and Sporulation | Persister Cells | Sporulation-associated proteins with broader functions | Terminal cytochrome d ubiquinol oxidases | Terminal cytochrome oxidases |
| Respiration | F0F1-type ATP synthase | Fumarate respiration cluster | Terminal cytochrome O ubiquinol oxidase | Respiratory Complex I |
|  | trimethylamine N-oxide (TMAO) reductase | Anaerobic respiratory reductases | NiFe hydrogenase maturation | Na(+)-translocating NADH-quinone oxidoreductase and rnf-like group of electron transport complexes |
|  | Succinate dehydrogenase | Respiratory dehydrogenases 1 | Hydrogenases | Formate hydrogenase |
|  | Formate dehydrogenase | Biogenesis of cytochrome c oxidases | NADH ubiquinone oxidoreductase | Biogenesis of c-type cytochromes |
|  | Quinone oxidoreductase family | Soluble cytochromes and functionally related electron carriers |  |  |
| Stress Response | Osmotic stress cluster | Choline and Betaine Uptake and Betaine Biosynthesis | Glutathione: Biosynthesis and gamma-glutamyl cycle | Glutathione: Redox cycle |
|  | Osmoregulation | NADPH:quinone oxidoreductase 2 | Oxidative stress | Glutaredoxins |
|  | Osmoprotectant ABC transporter YehZYXW of Enterobacteriales | Glutathione: Non-redox reactions | Protection from Reactive Oxygen Species | Tellurite resistance: Chromosomal determinants |
|  | Synthesis of osmoregulated periplasmic glucans | Redox-dependent regulation of nucleus processes | Glutathionylspermidine and Trypanothione | Glutathione-dependent pathway of formaldehyde detoxification |
|  | Heat shock dnaK gene cluster extended | Universal stress protein family | Cold shock, CspA family of proteins | Flavohaemoglobin |
|  | Uptake of selenate and selenite | Phage shock protein (psp) operon | Bacterial hemoglobins | Hfl operon |
|  | Carbon Starvation | Sugar-phosphate stress regulation | Commensurate regulon activation | Periplasmic Stress Response |
|  | Periplasmic Acid Stress Response in Enterobacteria |  |  |  |
| Metabolism of Aromatic Compounds | Quinate degradation | p-Hydroxybenzoate degradation | Benzoate degradation |  |
| Amino Acids and Derivatives | Glutamine, Glutamate, Aspartate and Asparagine Biosynthesis | Histidine Biosynthesis | Threonine degradation | Urea carboxylase and Allophanate hydrolase cluster |
|  | Glutamate dehydrogenases | Arginine Deiminase Pathway | Lysine Biosynthesis DAP Pathway | Lysine degradation |
|  | Glutamine synthetases | Polyamine Metabolism | Lysine Biosynthesis DAP Pathway, GJO scratch | Creatine and Creatinine Degradation |
|  | Glutamate and Aspartate uptake in Bacteria | Arginine and Ornithine Degradation | Methionine Degradation | Branched-Chain Amino Acid Biosynthesis |
|  | Methionine Biosynthesis | Arginine Biosynthesis extended | Threonine and Homoserine Biosynthesis | Leucine Biosynthesis |
|  | Cysteine Biosynthesis | Common Pathway For Synthesis of Aromatic Compounds (DAHP synthase to chorismate) | Chorismate: Intermediate for synthesis of Tryptophan, PAPA antibiotics, PABA, 3-hydroxyanthranilate and more. | Glycine Biosynthesis |
|  | Cysteine Biosynthesis | A Hypothetical Protein Related to Proline Metabolism | Phenylalanine and Tyrosine Branches from Chorismate | Alanine biosynthesis |
|  | Chorismate Synthesis | Proline, 4-hydroxyproline uptake and utilization | Tryptophan synthesis | Serine Biosynthesis |
|  | Proline Synthesis | Glycine cleavage system | Glycine and Serine Utilization |  |
| Sulfur Metabolism | Sulfite reduction-associated complex DsrMKJOP and co-clustering genes | L-Cystine Uptake and Metabolism | Thioredoxin-disulfide reductase | Alkanesulfonate assimilation |
|  | Inorganic Sulfur Assimilation | Taurine Utilization | Galactosylceramide and Sulfatide metabolism | Alkanesulfonates Utilization |
| Phosphorus Metabolism | High affinity phosphate transporter and control of PHO regulon | Phosphate metabolism | Polyphosphate | Alkylphosphonate utilization |
| Carbohydrates | Methylglyoxal Metabolism | Glyoxylate bypass | TCA Cycle | N-Acetyl-Galactosamine and Galactosamine Utilization |
|  | Pyruvate:ferredoxin oxidoreductase | Dihydroxyacetone kinases | TCA Cycle | Trehalose Biosynthesis |
|  | Pyruvate metabolism II: acetyl-CoA, acetogenesis from pyruvate | Glycolysis and Gluconeogenesis | Pyruvate metabolism I: anaplerotic reactions, PEP | Sucrose utilization |
|  | Pyruvate Alanine Serine Interconversions | Entner-Doudoroff Pathway | Pentose phosphate pathway | Lactose utilization |
|  | Trehalose Uptake and Utilization | Dehydrogenase complexes | Glycolate, glyoxylate interconversions | Maltose and Maltodextrin Utilization |
|  | Lactose and Galactose Uptake and Utilization | One-carbon metabolism by tetrahydropterines | Chitin and N-acetylglucosamine utilization | Melibiose Utilization |
|  | Formaldehyde assimilation: Ribulose monophosphate pathway | CitAB | Methylcitrate cycle | Beta-Glucoside Metabolism |
|  | Serine-glyoxylate cycle | Butanol Biosynthesis | Mannitol Utilization | Unknown sugar utilization (cluster yphABCDEFG) |
|  | Serine-glyoxylate cycle | Fermentations: Mixed acid | Ethanolamine utilization | beta-glucuronide utilization |
|  | Propionate-CoA to Succinate Module | Acetolactate synthase subunits | Uncharacterized sugar kinase cluster (ygc) | Unknown carbohydrate utilization containing Fructose-bisphosphate aldolase |
|  | Glycerate metabolism | Fermentations: Lactate | Carbon storage regulator | Glycogen metabolism |
|  | Lactate utilization | Acetyl-CoA fermentation to Butyrate | Unknown carbohydrate utilization ( cluster Ydj ) | Alpha-Amylase locus in *Streptocococcus* |
|  | Citrate Metabolism, Transport, and Regulation | Glycerol and Glycerol-3-phosphate Uptake and Utilization | Conserved cluster around inner membrane protein gene yghQ, probably involved in polysaccharide biosynthesis | D-allose utilization |
|  | Mannose Metabolism | D-galactarate, D-glucarate and D-glycerate catabolism - gjo | Xylose utilization | L-rhamnose utilization |
|  | Hexose Phosphate Uptake System | D-Galacturonate and D-Glucuronate Utilization | D-Tagatose and Galactitol Utilization | D-ribose utilization |
|  | D-gluconate and ketogluconates metabolism | L-fucose utilization temp | L-fucose utilization | D-Sorbitol(D-Glucitol) and L-Sorbose Utilization |
|  | Fructose utilization | D-galactonate catabolism | Deoxyribose and Deoxynucleoside Catabolism | D-galactarate, D-glucarate and D-glycerate catabolism |
|  | L-ascorbate utilization (and related gene clusters) | L-Arabinose utilization |  |  |
