# Supplementary material for: Multiomics Assessment of Gene Expression in a Clinical Strain of CTX-M-15-Producing ST131 Escherichia coli
Source: Front Microbiol. 2019 May 3;10:831. doi: 10.3389/fmicb.2019.00831 (PMC6509150; doi:10.3389/fmicb.2019.00831)
Supplement: TABLE S4 — Integration of proteomic data of interest with corresponding transcriptomic data. [file Table_4.DOCX]

**Supplementary Table S4.** Integration of proteomic data of interest with corresponding transcriptomic data.

| **Protein** | **Function** | **Proteome/Subproteome** | **Protein expression**  **(highest Mascot score)** | **RNA expression**  **(highest FPKM)** | **RNA Expression Ranking** |
| --- | --- | --- | --- | --- | --- |
| DNA protection during starvation Dps | Stress response | Whole cell | 196 | 97487,2 | 3 |
| Chaperone ClpB | Stress response | Cytoplasm | 376 | 12628,4 | 26 |
| Alkyl hydroperoxide reductase AhpC | Oxidoreductase | Membrane | 67 | 11575,1 | 29 |
| Chaperone DnaK | Stress response | Cytoplasm | 363 | 10020,4 | 34 |
| Elongation factor TufA | Antibiotic response | Cytoplasm | 256 | 5897,62 | 54 |
| Chaperone 60 kDa GroEL1 | Stress response | Cytoplasm | 264 | 4146,73 | 72 |
| Curved DNA-binding protein | Stress response | Cytoplasm | 84 | 3861,93 | 77 |
| Thiol peroxidase protein Tpx | Oxidoreductase | Whole cell | 71 | 1624,14 | 188 |
| β-lactamase TEM* | Antibiotic response | Periplasm | 36 | 1200,21 | 252 |
| RNA polymerase sigma factor RpoH* | Heat response | Membrane | 27 | 1187,82 | 256 |
| ATP-dependent protease ATPase subunit HslU | Stress response | Cytoplasm | 234 | 657,518 | 447 |
| SOS-response repressor and protease LexA | SOS response | Whole cell | 59 | 633,244 | 463 |
| Superoxide dismutase [Mn] SodA | Oxidoreductase | Whole cell | 120 | 531,198 | 539 |
| Malate dehydrogenase Mdh | Oxidoreductase | Cytoplasm | 186 | 324,797 | 767 |
| Two-component system connector protein SafA | Stress response | Whole cell | 71 | 293,969 | 832 |
| Outer membrane TolC* | Antibiotic response | Membrane | 35 | 235,374 | 971 |
| Enoyl-[acyl-carrier-protein] reductase NADH FabI | Antibiotic response | Ctyoplasm | 168 | 197,67 | 1107 |
| Bifunctional polymyxin resistance protein ArnA* | Antibiotic response | Membrane | 12 | 178,674 | 1188 |
| DNA replication and repair protein RecF | SOS response | Whole cell | 58 | 29,6381 | 3080 |

*Protein Mascot score below the defined minimum value to be considered a significant MS quantification.
