# Supplementary material for: Multiomics Assessment of Gene Expression in a Clinical Strain of CTX-M-15-Producing ST131 Escherichia coli
Source: Front Microbiol. 2019 May 3;10:831. doi: 10.3389/fmicb.2019.00831 (PMC6509150; doi:10.3389/fmicb.2019.00831)
Supplement: TABLE S5 — Identification of protein spots from 2DE gels of whole-cell extracts of ESBL-producing E. coli isolate C999 based on MALDI-TOF/MS sequencing results. [file Table_5.DOCX]

**Supplementary Table S5.** Identification of protein spots from whole-cell extraction of ESBL-producing *E. coli* isolate C999 using 2-DE gels and MALDI-TOF sequencing results.

| **Spot** | **Accession Number** | **Protein description** | **Species** | **Gene name** | **Protein MW** | **Protein pI** | **Mascot Score** | **Biological Process** | **References** |
| --- | --- | --- | --- | --- | --- | --- | --- | --- | --- |
| 1 | A7ZJM7 | DNA protection during starvation protein | *Escherichia coli* | *dps* | 18684 | 5.70 | 143 | Stress response | [[1](#_ENREF_1)] |
| 2 | P0AFH9 | Osmotically-inducible protein Y | *Escherichia coli* | *osm*Y | 21061 | 5.42 | 80 | - | [[2](#_ENREF_2)] |
| 3 | P50456 | Protein Mlc | *Escherichia coli* | *mlc* | 44516 | 9.64 | 71 | Transcription | [[3](#_ENREF_3)] |
| 4 | P00448 | Superoxide dismutase [Mn] | *Escherichia coli* | *sod*A | 23083 | 6.44 | 120 | Oxidoreductase | [[3](#_ENREF_3)] |
| 5 | A7ZHR2 | Ribosome-recycling factor | *Escherichia coli* | *frr* | 20683 | 6.43 | 64 | Protein biosynthesis | [[1](#_ENREF_1)] |
| 6 | A7ZJM7 | DNA protection during starvation protein | *Escherichia coli* | *dps* | 18684 | 5.70 | 196 | Stress response | [[1](#_ENREF_1)] |
| 7 | B7ULM8 | 2,3-bisphosphoglycerate-dependent phosphoglycerate mutase | *Escherichia coli* | *gpm*A | 28539 | 5.85 | 163 | Glycolysis | [[4](#_ENREF_4)] |
| 8 | P0ABK6 | Cysteine synthase A | *Escherichia coli* | *cys*K | 34525 | 5.83 | 243 | Transferase | [[5](#_ENREF_5)] |
| 9 | A7ZHR0 | Elongation factor Ts | *Escherichia coli* | *tsf* | 30518 | 5.22 | 227 | Protein biosynthesis | [[1](#_ENREF_1)] |
| 10 | A7ZSD0 | Malate dehydrogenase | *Escherichia coli* | *mdh* | 32488 | 5.61 | 81 | Tricarboxylic acid cycle;  Oxidoreductase | [[1](#_ENREF_1)] |
| 11 | A7ZPZ4 | Serine hydroxymethyltransferase | *Escherichia coli* | *gly*A | 45459 | 6.03 | 139 | Transferase | [[1](#_ENREF_1)] |
| 12 | P0A911 | Outer membrane protein A | *Escherichia coli* | *omp*A | 37292 | 5.60 | 102 | Transport | [[5](#_ENREF_5)] |
| 13 | P69784 | Glucose-specific phosphotransferase enzyme IIA component | *Escherichia coli* | *crr* | 18240 | 4.73 | 70 | Transport | [[2](#_ENREF_2)] |
| 14 | P0AE10 | Alkyl hydroperoxide reductase subunit C | *Escherichia coli* | *ahp*C | 20862 | 5.03 | 88 | Oxidoreductase | [[5](#_ENREF_5)] |
| 15 | A7ZII0 | UPF0234 protein YajQ | *Escherichia coli* | *yaj*Q | 18333 | 5.94 | 131 | - | [[1](#_ENREF_1)] |
| 16 | P0AAI7 | 3-oxoacyl-[acyl-carrier-protein] synthase 2 | *Escherichia coli* | *fab*F | 43247 | 5.71 | 88 | Fatty acid biosynthesis | [[5](#_ENREF_5)] |
| 17 | A7ZL79 | Tryptophan synthase beta chain | *Escherichia coli* | *trp*B | 43241 | 5.71 | 68 | Amino-acid biosynthesis; Lyase | [[1](#_ENREF_1)] |
| 18 | Q0TKK5 | Trigger factor | *Escherichia coli* | *tig* | 48221 | 4.81 | 334 | Cell division; Chaperone | [[6](#_ENREF_6)] |
| 19 | A1AJ51 | 60 kDa chaperonin 1 | *Escherichia coli* | *gro*L1 | 57464 | 4.85 | 294 | Protein refolding;  Chaperone;  Stress response | [[7](#_ENREF_7)] |
| 20 | A7ZTU4 | ATP synthase subunit beta | *Escherichia coli* | *atp*D | 50351 | 4.90 | 242 | Transport | [[1](#_ENREF_1)] |
| 21 | A7ZKF2 | Glucans biosynthesis protein G | *Escherichia coli* | *mdo*G | 57846 | 6.26 | 101 | Carbohydrate biosynthetic process | [[1](#_ENREF_1)] |
| 22 | P0ADG8 | Inosine-5'-monophosphate dehydrogenase | *Escherichia coli* | *gua*B | 52275 | 9.54 | 104 | GMP biosynthesis | [[5](#_ENREF_5)] |
| 23 | P0A719 | Ribose-phosphate pyrophosphokinase | *Escherichia coli* | *prs* | 34425 | 6.32 | 122 | Transferase | [[5](#_ENREF_5)] |
| 24 | A7ZTU6 | ATP synthase subunit alpha | *Escherichia coli* | *atp*A | 55416 | 5.80 | 129 | Transport | [[1](#_ENREF_1)] |
| 25 | P23843 | Periplasmic oligopeptide-binding protein | *Escherichia coli* | *opp*A | 60975 | 5.85 | 125 | Transport | [[3](#_ENREF_3)] |
| 26 | P0ABZ8 | Chaperone SurA | *Escherichia coli* | *sur*A | 47254 | 6.12 | 120 | Chaperone | [[5](#_ENREF_5)] |
| 27 | P76116 | Uncharacterized protein YncE | *Escherichia coli* | *ync*E | 38589 | 8.80 | 82 | - | [[3](#_ENREF_3)] |
| 28 | A7ZQP2 | Protein Xni | *Escherichia coli* | *xni* | 28433 | 6.20 | 61 | DNA binding | [[1](#_ENREF_1)] |
| 32 | A7ZPZ4 | Serine hydroxymethyltransferase | *Escherichia coli* | *gly*A | 45459 | 6.03 | 72 | Transferase | [[1](#_ENREF_1)] |
| 33 | A7ZPZ4 | Serine hydroxymethyltransferase | *Escherichia coli* | *gly*A | 45459 | 6.03 | 155 | Transferase | [[1](#_ENREF_1)] |
| 34 | P0A9B4 | Glyceraldehyde-3-phosphate dehydrogenase A | *Escherichia coli* | *gap*A | 35681 | 6.58 | 80 | Glycolysis | [[5](#_ENREF_5)] |
| 34 | P0AGJ6 | Uncharacterized tRNA/rRNA methyltransferase YfiF | *Escherichia coli* | *gfi*F | 37989 | 8.94 | 63 | Transferase | [[5](#_ENREF_5)] |
| 35 | P0AGF1 | Succinyl-CoA ligase [ADP-forming] subunit alpha | *Escherichia coli* | *suc*D | 30044 | 6.31 | 138 | Tricarboxylic acid cycle | [[5](#_ENREF_5)] |
| 36 | A7ZSR5 | Shikimate kinase 1 | *Escherichia coli* | *aro*K | 19526 | 5.26 | 129 | Transferase | [[1](#_ENREF_1)] |
| 37 | A7ZQC0 | S-ribosylhomocysteine lyase | *Escherichia coli* | *lux*S | 19575 | 8.05 | 60 | Autoinducer synthesis | [[1](#_ENREF_1)] |
| 38 | P0A864 | Thiol peroxidase | *Escherichia coli* | *tpx* | 17995 | 4.75 | 71 | Oxidoreductase | [[5](#_ENREF_5)] |
| 39 | P0A9B4 | Glyceraldehyde-3-phosphate dehydrogenase A | *Escherichia coli* | *gap*A | 35681 | 6.58 | 144 | Glycolysis | [[5](#_ENREF_5)] |
| 40 | P10018 | Uncharacterized 14.7 kDa protein | *Escherichia coli* |  | 14907 | 7.83 | 88 | - | [[8](#_ENREF_8)] |
| 41 | A7ZSL4 | Elongation factor Tu 1 | *Escherichia coli* | *tuf*1 | 43427 | 5.30 | 56 | Protein biosynthesis | [[1](#_ENREF_1)] |
| 41 | A7ZUJ2 | Elongation factor Tu 2 | *Escherichia coli* | *tuf*2 | 43456 | 5.36 | 57 | Protein biosynthesis | [[1](#_ENREF_1)] |
| 41 | P0A869 | Transaldolase A | *Escherichia coli* | *tal*A | 35865 | 5.89 | 178 | Pentose shunt; Transferase | [[5](#_ENREF_5)] |
| 42 | A7ZIN4 | Adenylate kinase | *Escherichia coli* | *adk* | 23628 | 5.47 | 126 | Transferase | [[1](#_ENREF_1)] |
| 43 | P0AEN0 | Cystine-binding periplasmic protein | *Escherichia coli* | *fli*Y | 29021 | 5.29 | 114 | Transport | [[2](#_ENREF_2)] |
| 44 | A7ZPU1 | Uracil phosphoribosyltransferase | *Escherichia coli* | *upp* | 22576 | 5.32 | 112 | Transferase | [[1](#_ENREF_1)] |
| 45 | P00642 | Type-2 restriction enzyme EcoRI | *Escherichia coli* | *eco*RIR | 31097 | 7.77 | 109 | Restriction system; Hydrolase | [[9](#_ENREF_9)] |
| 48 | P0A7B0 | Inorganic pyrophosphatase | *Escherichia coli* | *ppa* | 19805 | 5.03 | 80 | Hydrolase | [[5](#_ENREF_5)] |
| 49 | P0A957 | KHG/KDPG aldolase | *Escherichia coli* | *eda* | 22441 | 5.57 | 88 | Lyase | [[5](#_ENREF_5)] |
| 50 | P02925 | D-ribose-binding periplasmic protein | *Escherichia coli* | *rbs*B | 30931 | 5.99 | 63 | Transport | [[3](#_ENREF_3)] |
| 50 | Q46857 | 2,5-diketo-D-gluconic acid reductase A | *Escherichia coli* | *dkg*A | 31147 | 6.00 | 97 | Ascorbate biosynthesis; Oxidoreductase | [[3](#_ENREF_3)] |
| 51 | A7ZHS5 | Acetyl-coenzyme A carboxylase carboxyl transferase subunit alpha | *Escherichia coli* | *acc*A | 35333 | 5.76 | 170 | Fatty acid biosynthesi; Ligase | [[1](#_ENREF_1)] |
| 53 | P0AE20 | Methionine aminopeptidase | *Escherichia coli* | *map* | 29711 | 5.63 | 104 | Hydrolase | [[5](#_ENREF_5)] |
| 54 | P63884 | N-acetylmuramoyl-L-alanine amidase AmiC | *Escherichia coli* | *ami*C | 45606 | 9.41 | 71 | Lyase | [[2](#_ENREF_2)] |
| 55 | P0ADF7 | Phosphogluconate dehydratase | *Escherichia coli* | *edd* | 65054 | 5.93 | 98 | Lyase | [[5](#_ENREF_5)] |
| 56 | Q8XCG6 | 3'(2'),5'-bisphosphate nucleotidase CysQ | *Escherichia coli* | *cys*Q | 27314 | 5.59 | 88 | Hydrolase | [[5](#_ENREF_5)] |
| 57 | Q8X505 | NADP-dependent L-serine/L-allo-threonine dehydrogenase YdfG | *Escherichia coli* | *ydf*G | 27360 | 5.65 | 99 | Oxidoreductase | [[5](#_ENREF_5)] |
| 58 | A7ZVS4 | Deoxyribose-phosphate aldolase | *Escherichia coli* | *deo*C | 27958 | 5.50 | 118 | Lyase | [[1](#_ENREF_1)] |
| 59 | A7ZR12 | Glycine dehydrogenase [decarboxylating] | *Escherichia coli* | *gcv*P | 105066 | 5.62 | 152 | Oxidoreductase | [[1](#_ENREF_1)] |
| 60 | P09551 | Lysine-arginine-ornithine-binding periplasmic protein | *Escherichia coli* | *arg*T | 28088 | 5.19 | 117 | Transport | [[3](#_ENREF_3)] |
| 62 | P0A9P5 | Thioredoxin reductase | *Escherichia coli* | *trx*B | 34829 | 5.30 | 85 | Oxidoreductase | [[5](#_ENREF_5)] |
| 63 | P0ADS7 | Uncharacterized protein YggE | *Escherichia coli* | *ygg*E | 26619 | 6.10 | 97 | - | [[5](#_ENREF_5)] |
| 64 | P0AEY0 | Maltose-binding periplasmic protein | *Escherichia coli* | *mal*E | 43360 | 5.22 | 107 | Transport | [[5](#_ENREF_5)] |
| 67 | P33362 | Putative osmoprotectant uptake system substrate-binding protein OsmF | *Escherichia coli* | *osm*F | 32589 | 5.56 | 95 | Transport | [[3](#_ENREF_3)] |
| 70 | B7MH66 | Porphobilinogen deaminase | *Escherichia coli* | *hem*C | 34029 | 5.54 | 57 | Transferase | [[10](#_ENREF_10)] |
| 71 | P0A911 | Outer membrane protein A | *Escherichia coli* | *omp*A | 37292 | 5.60 | 99 | Transport | [[5](#_ENREF_5)] |
| 73 | P0AFG1 | Transcription antitermination protein NusG | *Escherichia coli* | *nus*G | 20518 | 6.33 | 116 | Transcription | [[5](#_ENREF_5)] |
| 74 | B7UFQ9 | L-rhamnonate dehydratase | *Escherichia coli* | *rhm*D | 45291 | 5.64 | 72 | Lyase | [[4](#_ENREF_4)] |
| 75 | P0AFR5 | Uncharacterized protein YciO | *Escherichia coli* | *yci*O | 23368 | 5.97 | 72 | - | [[2](#_ENREF_2)] |
| 76 | B7UNY3 | Curved DNA-binding protein | *Escherichia coli* | *cbp*A | 34464 | 6.33 | 87 | Protein folding;  Chaperone;  Stress response | [[4](#_ENREF_4)] |
| 75 | P0AFR5 | Uncharacterized protein YciO | *Escherichia coli* | *yci*O | 23368 | 5.97 | 72 | - | [[2](#_ENREF_2)] |
| 76 | B7UNY3 | Curved DNA-binding protein | *Escherichia coli* | *cbp*A | 34464 | 6.33 | 87 | Protein folding; Chaperone; Stress response | [[4](#_ENREF_4)] |
| 77 | A7ZVS6 | Phosphopentomutase | *Escherichia coli* | *deo*B | 44684 | 5.11 | 204 | Isomerase | [[1](#_ENREF_1)] |
| 78 | A7ZJE2 | 6-phosphogluconolactonase | *Escherichia coli* | *pgl* | 36570 | 5.06 | 91 | Carbohydrate metabolism; Hydrolase | [[1](#_ENREF_1)] |
| 79 | P0A9S6 | Glycerol dehydrogenase | *Escherichia coli* | *gld*A | 39087 | 4.79 | 94 | Glycerol metabolism; Oxidoreductase | [[2](#_ENREF_2)] |
| 80 | P06999 | 6-phosphofructokinase isozyme 2 | *Escherichia coli* | *pfk*B | 32664 | 5.48 | 115 | Glycolysis | [[3](#_ENREF_3)] |
| 82 | A7ZS61 | Polyribonucleotide nucleotidyltransferase | *Escherichia coli* | *pnp* | 77111 | 5.08 | 121 | Transferase | [[1](#_ENREF_1)] |
| 83 | P63285 | Chaperone protein ClpB | *Escherichia coli* | *clp*B | 95697 | 5.37 | 106 | Stress response | [[5](#_ENREF_5)] |
| 83 | P63285 | Chaperone protein ClpB | *Escherichia coli* | *clp*B | 95697 | 5.37 | 106 | Stress response | [[5](#_ENREF_5)] |
| 85 | P36683 | Aconitate hydratase 2 | *Escherichia coli* | *acn*B | 94009 | 5.24 | 116 | Tricarboxylic acid cycle; Lyase | [[3](#_ENREF_3)] |
| 87 | A7ZUD3 | Triosephosphate isomerase | *Escherichia coli* | *tpi*A | 27126 | 5.64 | 98 | Glycolysis | [[1](#_ENREF_1)] |
| 88 | Q05433 | Chaperone protein ClpE | *Escherichia coli* | *clp*E | 29080 | 9.11 | 57 | Chaperone | [[11](#_ENREF_11)] |
| 90 | P0A6A5 | Acetate kinase | *Escherichia coli* | *ack*A | 43605 | 5.85 | 162 | Transferase | [[5](#_ENREF_5)] |
| 92 | P0A9P2 | Dihydrolipoyl dehydrogenase | *Escherichia coli* | *lpd*A | 50942 | 5.79 | 172 | Glycolysis | [[5](#_ENREF_5)] |
| 93 | P0AD62 | Pyruvate kinase I | *Escherichia coli* | *pyk*F | 51039 | 5.23 | 182 | Glycolysis | [[5](#_ENREF_5)] |
| 94 | P63884 | N-acetylmuramoyl-L-alanine amidase AmiC | *Escherichia coli* | *ami*C | 45606 | 9.41 | 73 | Lyase | [[2](#_ENREF_2)] |
| 95 | P0ADG8 | Inosine-5'-monophosphate dehydrogenase | *Escherichia coli* | *gua*B | 52275 | 9.25 | 81 | GMP biosynthesis | [[5](#_ENREF_5)] |
| 96 | P0AC43 | Succinate dehydrogenase flavoprotein subunit | *Escherichia coli* | *sdh*A | 65008 | 5.85 | 93 | Transport | [[5](#_ENREF_5)] |
| 97 | P39451 | Alcohol dehydrogenase, propanol-preferring | *Escherichia coli* | *adh*P | 35870 | 5.94 | 67 | Oxidoreductase | [[3](#_ENREF_3)] |
| 100 | Q0TIF5 | 2-dehydro-3-deoxyphosphooctonate aldolase | *Escherichia coli* | *kds*A | 31081 | 6.32 | 72 | Transferase | [[6](#_ENREF_6)] |
| 102 | A1AJ51 | 60 kDa chaperonin 1 | *Escherichia coli* | *gro*L1 | 57464 | 4.85 | 201 | Protein refolding; Chaperone; Stress response | [[7](#_ENREF_7)] |
| 102 | P19779 | Insertion element IS2 uncharacterized 16.4 kDa protein (ORF4) | *Escherichia coli* |  | 16665 | 9.55 | 64 | - | [[12](#_ENREF_12)] |
| 102 | P64634 | Putative DNA utilization protein HofN | *Escherichia coli* | *hof*N | 20783 | 10.93 | 44 | Cell membrane | [[3](#_ENREF_3)] |
| 103 | A7ZTU4 | ATP synthase subunit beta | *Escherichia coli* | *atp*D | 50351 | 4.90 | 89 | Transport | [[1](#_ENREF_1)] |
| 104 | A7ZTU6 | ATP synthase subunit alpha | *Escherichia coli* | *atp*A | 55416 | 5.80 | 161 | Transport | [[1](#_ENREF_1)] |
| 105 | P0AGF1 | Succinyl-CoA ligase [ADP-forming] subunit alpha | *Escherichia coli* | *suc*D | 30044 | 6.32 | 113 | Tricarboxylic acid cycle | [[5](#_ENREF_5)] |
| 107 | A7ZSJ7 | 50S ribosomal protein L5 | *Escherichia coli* | *rpl*E | 20346 | 9.49 | 146 | Translation | [[1](#_ENREF_1)] |
| 108 | A7ZSJ4 | 50S ribosomal protein L6 | *Escherichia coli* | *rpl*F | 18949 | 9.71 | 148 | Translation | [[1](#_ENREF_1)] |
| 108 | B7USA1 | Translation initiation factor IF-3 | *Escherichia coli* | *inf*C | 20608 | 9.54 | 60 | Protein biosynthesis | [[4](#_ENREF_4)] |
| 109 | A7ZJW2 | Seryl-tRNA synthetase | *Escherichia coli* | *ser*S | 48669 | 5.34 | 138 | Protein biosynthesis | [[1](#_ENREF_1)] |
| 110 | P0AFK1 | Protein PmbA | *Escherichia coli* | *pmb*A | 48625 | 5.40 | 142 | - | [[5](#_ENREF_5)] |
| 111 | P0ACA5 | Stringent starvation protein A | *Escherichia coli* | *ssp*A | 24346 | 5.22 | 74 | - | [[5](#_ENREF_5)] |
| 112 | P0A9Q3 | Aerobic respiration control protein ArcA | *Escherichia coli* | *arc*A | 27389 | 5.20 | 138 | Transcription | [[5](#_ENREF_5)] |
| 113 | P0A9G7 | Isocitrate lyase | *Escherichia coli* | *ace*A | 47777 | 5.16 | 109 | Glyoxylate bypass; Lyase | [[2](#_ENREF_2)] |
| 114 | A7ZHA4 | Chaperone protein DnaK (HSP70) | *Escherichia coli* | *dna*K | 69130 | 4.83 | 185 | Stress response | [[1](#_ENREF_1)] |
| 115 | P0AFW8 | Protein Rof | *Escherichia coli* | *rof* | 9588 | 4.63 | 21 | Regulation of transcription termination | [[3](#_ENREF_3)] |
| 115 | P0AG69 | 30S ribosomal protein S1 | *Escherichia coli* | *rps*A | 61235 | 4.88 | 91 | Translation | [[5](#_ENREF_5)] |
| 116 | A7ZQM2 | Enolase | *Escherichia coli* | *eno* | 45683 | 5.32 | 56 | Glycolysis | [[1](#_ENREF_1)] |
| 117 | A7ZSL4 | Elongation factor Tu 1 | *Escherichia coli* | *tuf*1 | 43427 | 5.30 | 70 | Protein biosynthesis | [[1](#_ENREF_1)] |
| 117 | P0AB72 | Fructose-bisphosphate aldolase class 2 | *Escherichia coli* | *fba*A | 39351 | 5.52 | 94 | Glycolysis | [[5](#_ENREF_5)] |
| 118 | A7ZSL4 | Elongation factor Tu 1 | *Escherichia coli* | *tuf*1 | 43427 | 5.30 | 257 | Protein biosynthesis | [[1](#_ENREF_1)] |
| 119 | A7ZSL4 | Elongation factor Tu 1 | *Escherichia coli* | *tuf*1 | 43427 | 5.30 | 88 | Protein biosynthesis | [[1](#_ENREF_1)] |
| 120 | A7ZSL4 | Elongation factor Tu 1 | *Escherichia coli* | *tuf*1 | 43427 | 5.30 | 273 | Protein biosynthesis | [[1](#_ENREF_1)] |
| 121 | P08200 | Isocitrate dehydrogenase [NADP] | *Escherichia coli* | *icd* | 46070 | 5.15 | 98 | Tricarboxylic acid cycle; Oxidoreductase | [[3](#_ENREF_3)] |
| 122 | A7ZV47 | Adenylosuccinate synthetase | *Escherichia coli* | *pur*A | 47543 | 5.32 | 104 | Purine biosynthesis; Ligase | [[1](#_ENREF_1)] |
| 124 | A7ZMK7 | NH(3)-dependent NAD(+) synthetase | *Escherichia coli* | *nad*E | 30790 | 5.30 | 98 | Ligase | [[1](#_ENREF_1)] |
| 125 | B1LFB6 | Two-component-system connector protein SafA | *Escherichia coli* | *saf*A | 7386 | 6.70 | 71 | Stress response | [[13](#_ENREF_13)] |
| 126 | B7NM00 | LPS-assembly lipoprotein IptE | *Escherichia coli* | *lpt*E | 21332 | 5.91 | 100 | Cell membrane | [[10](#_ENREF_10)] |
| 128 | A7ZMA4 | Transcriptional regulator SlyA | *Escherichia coli* | *sly*A | 16400 | 6.60 | 60 | Transcription | [[1](#_ENREF_1)] |
| 129 | P0AEK5 | Enoyl-[acyl-carrier-protein] reductase [NADH] | *Escherichia coli* | *fab*I | 28074 | 5.58 | 79 | Antibiotic resistance | [[5](#_ENREF_5)] |
| 130 | A7ZMA4 | Transcriptional regulator SlyA | *Escherichia coli* | *sly*A | 16400 | 6.60 | 81 | Transcription | [[1](#_ENREF_1)] |
| 131 | P65765 | FKBP-type peptidyl-prolyl cis-trans isomerase FkpA | *Escherichia coli* | *fkp*A | 28894 | 6.73 | 69 | Isomerase; Protein Folding | [[5](#_ENREF_5)] |
| 132 | B7UMJ8 | ATP synthase gamma chain | *Escherichia coli* | *atp*G | 31671 | 8.84 | 69 | Transport | [[4](#_ENREF_4)] |
| 133 | P63884 | N-acetylmuramoyl-L-alanine amidase AmiC | *Escherichia coli* | *ami*C | 45606 | 9.41 | 96 | Lyase | [[2](#_ENREF_2)] |
| 134 | P0AG32 | Transcription termination factor Rho | *Escherichia coli* | *rho* | 47032 | 6.75 | 82 | Transcription | [[5](#_ENREF_5)] |
| 135 | A7ZSI5 | 30S ribosomal protein S4 | *Escherichia coli* | *rps*D | 23512 | 10.05 | 64 | Translation | [[1](#_ENREF_1)] |
| 136 | A7ZSK9 | 50S ribosomal protein L3 | *Escherichia coli* | *rpl*C | 22230 | 9.90 | 105 | Translation | [[1](#_ENREF_1)] |
| 137 | B7L4K9 | 50S ribosomal protein L3 | *Escherichia coli* | *rpl*C | 22230 | 9.90 | 85 | Translation | [[10](#_ENREF_10)] |
| 138 | A7ZUJ7 | 50S ribosomal protein L1 | *Escherichia coli* | *rpl*A | 24714 | 5.77 | 72 | Translation regulation | [[1](#_ENREF_1)] |
| 139 | A7ZUJ7 | 50S ribosomal protein L1 | *Escherichia coli* | *rpl*A | 24714 | 9.64 | 96 | Translation regulation | [[1](#_ENREF_1)] |
| 140 | A7ZVR0 | Ribosomal RNA small subunit methyltransferase C | *Escherichia coli* | *rsm*C | 37918 | 6.00 | 89 | rRNA processing; Transferase | [[1](#_ENREF_1)] |
| 141 | P09147 | UDP-glucose 4-epimerase | *Escherichia coli* | *gal*E | 37413 | 5.88 | 109 | Carbohydrate metabolism; Isomerase | [[3](#_ENREF_3)] |
| 142 | Q05129 | Protein EaeB | *Escherichia coli* | *eae*B | 33121 | 5.24 | 59 | Transferase | [[4](#_ENREF_4)] |
| 143 | P75691 | Uncharacterized zinc-type alcohol dehydrogenase-like protein YahK | *Escherichia coli* | *yah*K | 38524 | 5.80 | 74 | Oxidoreductase | [[3](#_ENREF_3)] |
| 144 | P0A6A5 | Acetate kinase | *Escherichia coli* | *ack*A | 43605 | 5.85 | 86 | Transferase | [[5](#_ENREF_5)] |
| 146 | A7ZI52 | HTH-type transcriptional regulator BetI | *Escherichia coli* | *bet*I | 21776 | 10.16 | 61 | Transcription | [[1](#_ENREF_1)] |
| 147 | A7ZUR5 | LexA repressor | *Escherichia coli* | *lex*A | 22344 | 9.64 | 59 | SOS response;  Transcription | [[1](#_ENREF_1)] |
| 150 | Q47133 | F1845 adhesin operon regulatory protein | *Escherichia coli* | *daa*A | 9843 | 7.94 | 72 | Transcription | [[14](#_ENREF_14)] |
| 151 | P26646 | Putative quinone oxidoreductase YhdH | *Escherichia coli* | *yhd*H | 34873 | 5.63 | 90 | Oxidoreductase | [[3](#_ENREF_3)] |
| 152 | P0A9C3 | Aldose 1-epimerase | *Escherichia coli* | *gal*M | 38395 | 4.82 | 102 | Carbohydrate metabolism; Isomerase | [[3](#_ENREF_3)] |
| 153 | B1LFB6 | Two-component-system connector protein SafA | *Escherichia coli* | *saf*A | 7386 | 6.70 | 58 | Stress response | [[13](#_ENREF_13)] |
| 154 | P0A9C7 | Glutamine synthetase | *Escherichia coli* | *gln*A | 52099 | 5.26 | 89 | Ligase | [[5](#_ENREF_5)] |
| 157 | B7UM08 | DNA protection during starvation protein | *Escherichia coli* | *dps* | 18684 | 5.70 | 156 | Stress response | [[4](#_ENREF_4)] |
| 158 | P0A6W7 | Transcription elongation factor greA | *Escherichia coli* | *gre*A | 17687 | 4.71 | 175 | Transcription | [[5](#_ENREF_5)] |
| 161 | P0AEZ5 | Septum site-determining protein MinD | *Escherichia coli* | *min*D | 29710 | 5.25 | 147 | Cell cycle | [[5](#_ENREF_5)] |
| 164 | A7ZUE6 | ATP-dependent protease ATPase subunit HslU | *Escherichia coli* | *hsl*U | 49676 | 5.24 | 81 | Stress response | [[1](#_ENREF_1)] |
| 165 | A7ZRT3 | Bifunctional protein HldE | *Escherichia coli* | *hld*E | 51247 | 5.29 | 71 | Transferase | [[1](#_ENREF_1)] |
| 166 | P0AF00 | Molybdenum cofactor biosynthesis protein B | *Escherichia coli* | *moa*B | 18768 | 5.73 | 60 | Molybdenum cofactor biosynthesis | [[5](#_ENREF_5)] |
| 167 | A7ZJ31 | Leucyl-tRNA synthetase | *Escherichia coli* | *lleu*S | 97768 | 5.16 | 82 | Protein biosynthesis | [[1](#_ENREF_1)] |
| 167 | B7UNZ5 | Pyrimidine monooxygenase RutA | *Escherichia coli* | *rut*A | 40146 | 5.06 | 73 | rRNA processing; Transferase | [[4](#_ENREF_4)] |
| 169 | A7ZTQ6 | DNA replication and repair protein | *Escherichia coli* | *rec*F | 40717 | 6.78 | 58 | SOS response | [[1](#_ENREF_1)] |
| 170 | P0AC61 | Glutaredoxin-2 | *Escherichia coli* | *grx*B | 24449 | 7.72 | 68 | Transport | [[5](#_ENREF_5)] |
| 171 | B7MTC8 | Thymidine phosphorylase | *Escherichia coli* | *deo*A | 47349 | 5.27 | 69 | Transferase | [[10](#_ENREF_10)] |
| 177 | Q8FE70 | tRNA-modifying protein YgfZ | *Escherichia coli* | *ygf*Z | 36350 | 5.26 | 67 | tRNA processing | [[2](#_ENREF_2)] |
| 178 | P63884 | N-acetylmuramoyl-L-alanine amidase AmiC | *Escherichia coli* | *ami*C | 45606 | 9.41 | 57 | Lyase | [[2](#_ENREF_2)] |
| 179 | P0A9A8 | Cell division protein FtsZ | *Escherichia coli* | *fts*Z | 40299 | 4.63 | 111 | Cell cycle | [[5](#_ENREF_5)] |
| 181 | Q8XAS6 | UPF0409 protein YcdO | *Escherichia coli* | *ycd*O | 41212 | 4.86 | 61 | - | [[5](#_ENREF_5)] |
| 182 | P13477 | Colicin-E6 immunity protein | *Escherichia coli* | *imm* | 10124 | 6.02 | 95 | Bacteriocin immunity | [[15](#_ENREF_15)] |
| 182 | P76270 | Protein YebR | *Escherichia coli* | *yeb*R | 18281 | 4.66 | 56 | - | [[3](#_ENREF_3)] |
| 183 | P77499 | Probable ATP-dependent transporter SufC | *Escherichia coli* | *suf*C | 27622 | 4.84 | 96 | Transport | [[3](#_ENREF_3)] |
| 184 | P63884 | N-acetylmuramoyl-L-alanine amidase AmiC | *Escherichia coli* | *ami*C | 45606 | 9.41 | 65 | Lyase | [[2](#_ENREF_2)] |
| 185 | Q46861 | UPF0313 protein YgiQ | *Escherichia coli* | *ygi*Q | 84223 | 9.24 | 86 | 6 iron, 4 sulfur cluster binding | [[3](#_ENREF_3)] |
| 186 | B7UNZ5 | Pyrimidine monooxygenase RutA | *Escherichia coli* | *rut*A | 40146 | 5.06 | 108 | rRNA processing; Transferase | [[4](#_ENREF_4)] |
| 187 | A7ZSL4 | Elongation factor Tu 1 | *Escherichia coli* | *tuf*1 | 43427 | 5.30 | 174 | Protein biosynthesis | [[1](#_ENREF_1)] |
| 188 | A7ZHR0 | Elongation factor Ts | *Escherichia coli* | *tsf* | 30518 | 5.22 | 88 | Protein biosynthesis | [[1](#_ENREF_1)] |
| 190 | P0ABU3 | GTP-dependent nucleic acid-binding protein EngD | *Escherichia coli* | *eng*D | 39984 | 4.87 | 106 | GTP binding | [[5](#_ENREF_5)] |
| 192 | P0A797 | 6-phosphofructokinase isozyme 1 | *Escherichia coli* | *pfk*A | 35162 | 5.47 | 79 | Glycolysis | [[5](#_ENREF_5)] |
| 193 | A7ZMA4 | Transcriptional regulator SlyA | *Escherichia coli* | *sly*A | 16400 | 6.60 | 60 | Transcription | [[1](#_ENREF_1)] |

[1] Rasko DA, Rosovitz MJ, Myers GS, Mongodin EF, Fricke WF, Gajer P, et al. The pangenome structure of *Escherichia coli*: comparative genomic analysis of *E. coli* commensal and pathogenic isolates. Journal of bacteriology. 2008;190:6881-93.

[2] Welch RA, Burland V, Plunkett G, 3rd, Redford P, Roesch P, Rasko D, et al. Extensive mosaic structure revealed by the complete genome sequence of uropathogenic *Escherichia coli*. Proceedings of the National Academy of Sciences of the United States of America. 2002;99:17020-4.

[3] Blattner FR, Plunkett G, 3rd, Bloch CA, Perna NT, Burland V, Riley M, et al. The complete genome sequence of *Escherichia coli* K-12. Science. 1997;277:1453-62.

[4] Iguchi A, Thomson NR, Ogura Y, Saunders D, Ooka T, Henderson IR, et al. Complete genome sequence and comparative genome analysis of enteropathogenic *Escherichia coli* O127:H6 strain E2348/69. Journal of bacteriology. 2009;191:347-54.

[5] Perna NT, Plunkett G, 3rd, Burland V, Mau B, Glasner JD, Rose DJ, et al. Genome sequence of enterohaemorrhagic *Escherichia coli* O157:H7. Nature. 2001;409:529-33.

[6] Hochhut B, Wilde C, Balling G, Middendorf B, Dobrindt U, Brzuszkiewicz E, et al. Role of pathogenicity island-associated integrases in the genome plasticity of uropathogenic *Escherichia coli* strain 536. Molecular microbiology. 2006;61:584-95.

[7] Johnson TJ, Kariyawasam S, Wannemuehler Y, Mangiamele P, Johnson SJ, Doetkott C, et al. The genome sequence of avian pathogenic *Escherichia coli* strain O1:K1:H7 shares strong similarities with human extraintestinal pathogenic *E. coli* genomes. Journal of bacteriology. 2007;189:3228-36.

[8] Hall RM, Vockler C. The region of the IncN plasmid R46 coding for resistance to beta-lactam antibiotics, streptomycin/spectinomycin and sulphonamides is closely related to antibiotic resistance segments found in IncW plasmids and in Tn21-like transposons. Nucleic acids research. 1987;15:7491-501.

[9] Greene PJ, Gupta M, Boyer HW, Brown WE, Rosenberg JM. Sequence analysis of the DNA encoding the Eco RI endonuclease and methylase. The Journal of biological chemistry. 1981;256:2143-53.

[10] Touchon M, Hoede C, Tenaillon O, Barbe V, Baeriswyl S, Bidet P, et al. Organised genome dynamics in the *Escherichia coli* species results in highly diverse adaptive paths. PLoS genetics. 2009;5:e1000344.

[11] Bertin Y, Girardeau JP, Der Vartanian M, Martin C. The ClpE protein involved in biogenesis of the CS31A capsule-like antigen is a member of a periplasmic chaperone family in gram-negative bacteria. FEMS microbiology letters. 1993;108:59-67.

[12] Ronecker HJ, Rak B. Genetic organization of insertion element IS2 based on a revised nucleotide sequence. Gene. 1987;59:291-6.

[13] Fricke WF, Wright MS, Lindell AH, Harkins DM, Baker-Austin C, Ravel J, et al. Insights into the environmental resistance gene pool from the genome sequence of the multidrug-resistant environmental isolate *Escherichia coli* SMS-3-5. Journal of bacteriology. 2008;190:6779-94.

[14] Bilge SS, Apostol JM, Jr., Fullner KJ, Moseley SL. Transcriptional organization of the F1845 fimbrial adhesin determinant of *Escherichia coli*. Molecular microbiology. 1993;7:993-1006.

[15] Lau PC, Condie JA. Nucleotide sequences from the colicin E5, E6 and E9 operons: presence of a degenerate transposon-like structure in the ColE9-J plasmid. Molecular & general genetics : MGG. 1989;217:269-77.
