# Supplementary material for: Multiomics Assessment of Gene Expression in a Clinical Strain of CTX-M-15-Producing ST131 Escherichia coli
Source: Front Microbiol. 2019 May 3;10:831. doi: 10.3389/fmicb.2019.00831 (PMC6509150; doi:10.3389/fmicb.2019.00831)
Supplement: TABLE S6 — Identification of protein spots from 2DE gels of cytoplasmic extracts of ESBL-producing E. coli isolate C999 based on MALDI-TOF/MS sequencing results. [file Table_6.DOCX]

**Supplementary Table S6.** Identification of protein spots from cytoplasm extraction of ESBL-producing *E. coli* isolate C999 using 2-DE gels and MALDI-TOF sequencing results.

| **Spot** | **Accession Number** | **Protein name** | **Species** | **Gene name** | **Protein MW** | **Protein pI** | **Mascot Score** | **Biological Process** | **Reference** |
| --- | --- | --- | --- | --- | --- | --- | --- | --- | --- |
| 1 | E8J0Z2_ECO57 | Molecular chaperone DnaK | *Escherichia coli* | *dna*K | 69130 | 4.68 | 363 | Stress response | [[1](#_ENREF_1)] |
| 2 | E8JCJ0_ECO57 | 30S ribosomal protein S1 | *Escherichia coli* | *rps*A | 61235 | 4.74 | 202 | Translation | [[1](#_ENREF_1)] |
| 3 | E8J9G4_ECO57 | Chaperonin GroEL | *Escherichia coli* | *gro*EL | 57464 | 4.69 | 264 | Protein refolding | [[1](#_ENREF_1)] |
| 4 | T2G1X2_ECOLX | MULTISPECIES: peptidase M54 | *Escherichia coli* | *nus*A | 55008.00 | 4.40 | 67 | Transcription | [[2](#_ENREF_2)] |
| 5 | S1LQL1_ECOLX | Phosphoenolpyruvate-protein phosphotransferase | *Escherichia coli* | *A31E_02353* | 63704.00 | 4.60 | 94 | Kinase activity | [[3](#_ENREF_3)] |
| 6 | S1LQL1_ECOLX | Phosphoenolpyruvate-protein phosphotransferase | *Escherichia coli* | *A31E_02353* | 63704.00 | 4.60 | 105 | Kinase activity | [[3](#_ENREF_3)] |
| 7 | A0A084Z417_ECOLX | Cell division trigger factor | *Escherichia coli* | *tig* | 47836.00 | 4.70 | 133 | Cell division | [[4](#_ENREF_4)] |
| 7 | E8JAW0_ECO57 | Trigger factor | *Escherichia coli* | *tig* | 48163 | 4.68 | 132 | Protein folding | [[1](#_ENREF_1)] |
| 8 | E8JAZ7_ECO57 | Heat shock protein 90 | *Escherichia coli* | *htp*G | 71378 | 4.95 | 226 | Stress response | [[1](#_ENREF_1)] |
| 9 | S1MCR1_ECOLX | Prolyl-tRNA synthetase | *Escherichia coli* | *pro*S | 63640 | 4.94 | 134 | Aminoacyl-tRNA editing activity | [[1](#_ENREF_1)] |
| 12 | V6PVB7_ECOLX | MULTISPECIES: Polynucleotide phosphorylase/polyadenylase | *Escherichia coli* | *ECC1470_05514* | 74698.00 | 4.90 | 150 | RNA processing | [[5](#_ENREF_5)] |
| 14 | ACKA_ECOLI | MULTISPECIES: Acetate kinase | *Escherichia coli* | *ack*A | 43601.00 | 5.80 | 214 | Acetate biosynthesis | [[6](#_ENREF_6)] |
| 14 | E8J3T3_ECO57 | Acetate kinase A/propionate kinase 2 | *Escherichia coli* | *ack*A | 43605 | 5.83 | 214 | Acetate biosynthesis | [[1](#_ENREF_1)] |
| 15 | FABF_ECOLI | MULTISPECIES: 3-oxoacyl-ACP synthase | *Escherichia coli* | *fab*F | 41963.00 | 5.30 | 83 | Fatty acid biosynthesis | [[6](#_ENREF_6)] |
| 15 | FABF_ECOLI | 3-oxoacyl-[acyl-carrier-protein] synthase 2 | *Escherichia coli* | *fab*F | 41963 | 5.31 | 83 | Fatty acid biosynthesis | [[6](#_ENREF_6)] |
| 15 | E8JD43_ECO57 | 3-oxoacyl-(acyl carrier protein) synthase II | *Escherichia coli* | *fab*F | 43247 | 5.68 | 83 | Fatty acid biosynthesis | [[1](#_ENREF_1)] |
| 15 | FABF_ECOLI | MULTISPECIES: 3-oxoacyl-ACP synthase | *Escherichia coli* | *fab*F | 41963.00 | 5.30 | 83 | Fatty acid biosynthesis | [[6](#_ENREF_6)] |
| 16 | PYRG_ECOLI | MULTISPECIES: CTP synthetase | *Escherichia coli* | *pyr*G | 59954.00 | 5.60 | 125 | de novo' CTP biosynthesis | [[6](#_ENREF_6)] |
| 16 | E8IQN2_ECOLX | CTP synthetase | *Escherichia coli* | *pyr*G | 60796 | 5.49 | 125 | de novo' CTP biosynthesis | [[1](#_ENREF_1)] |
| 17 | Q8VP34_ECOLX | MULTISPECIES: Dihydrolipoamide dehydrogenase | *Escherichia coli* | *lpd*A | 50942.00 | 5.80 | 166 | Oxidoreductase activity | [[7](#_ENREF_7)] |
| 17 | E8I8E8_ECOLX | Dihydrolipoamide dehydrogenase | *Escherichia coli* | *lpd*A | 50942 | 5.75 | 166 | Cell redox homeostasis | [[1](#_ENREF_1)] |
| 17 | A0A084Z371_ECOLX | Dihydrolipoamide dehydrogenase/dihydrolipoamide dehydrogenase of pyruvate dehydrogenase complex | *Escherichia coli* | *lpd*A | 50942 | 5.75 | 166 |  | [[4](#_ENREF_4)] |
| 19 | FUMB_ECOLI | MULTISPECIES: Fumarate hydratase | *Escherichia coli* | *fum*B | 60551 | 5.86 | 108 | Cellular response to DNA damage stimulus | [[6](#_ENREF_6)] |
| 19 | A0A084Z146_ECOLX | Fumarate hydratase class I, anaerobic | *Escherichia coli* | *fum*B | 60592 | 5.77 | 108 |  | [[4](#_ENREF_4)] |
| 19 | FUMB_ECOLI | Fumarase B | *Escherichia coli* | *fum*B | 60581 | 5.86 | 108 | Cellular response to DNA damage stimulus | [[6](#_ENREF_6)] |
| 20 | AHPF_ECOLI | MULTISPECIES: Alkyl hydroperoxide reductase subunit F | *Escherichia coli* | *ahp*F | 54594.00 | 5.30 | 197 | Cellular response to reactive oxygen species | [[6](#_ENREF_6)] |
| 21 | AHPF_ECOLI | MULTISPECIES: Alkyl hydroperoxide reductase subunit F | *Escherichia coli* | *ahp*F | 55910 | 5.46 | 182 | Cellular response to reactive oxygen species | [[6](#_ENREF_6)] |
| 22 | DCEA_ECOLI | MULTISPECIES: Glutamate decarboxylase | *Escherichia coli* | *gad*A | 53221.00 | 5.10 | 141 | Glutamate metabolic process | [[6](#_ENREF_6)] |
| 22 | DCEA_ECOLI | Glutamate decarboxylase A subunit | *Escherichia coli* | *gad*A | 53221 | 5.10 | 141 | Glutamate metabolic process | [[6](#_ENREF_6)] |
| 22 | AHPF_ECOLI | MULTISPECIES: Alkyl hydroperoxide reductase subunit F | *Escherichia coli* | *ahp*F | 56483 | 5.46 | 181 | Cellular response to reactive oxygen species | [[6](#_ENREF_6)] |
| 22 | E8JEG4_ECO57 | Glutamate decarboxylase beta | *Escherichia coli* | *gad*A | 53208.00 | 5.10 | 136 |  | [[1](#_ENREF_1)] |
| 23 | DCEA_ECOLI | MULTISPECIES: Glutamate decarboxylase | *Escherichia coli* | *gad*A | 53204 | 5.17 | 83 | Glutamate metabolic process | [[6](#_ENREF_6)] |
| 23 | E8JEG4_ECO57 | Glutamate decarboxylase beta | *Escherichia coli* | *gad*A | 53208 | 5.10 | 83 | Glutamate metabolic process | [[1](#_ENREF_1)] |
| 23 | DCEA_ECOLI | Glutamate decarboxylase, partial | *Escherichia coli* | *gad*A | 47995.00 | 5.00 | 109 | Glutamate metabolic process | [[6](#_ENREF_6)] |
| 23 | DCEA_ECOLI | Glutamate decarboxylase A subunit | *Escherichia coli* | *gad*A | 53221 | 5.10 | 106 | Glutamate metabolic process | [[6](#_ENREF_6)] |
| 23 | AHPF_ECOLI | MULTISPECIES: Alkyl hydroperoxide reductase subunit F | *Escherichia coli* | *ahp*F | 56484 | 5.37 | 181 | Cellular response to reactive oxygen species | [[6](#_ENREF_6)] |
| 23 | DCEA_ECOLI | Glutamate decarboxylase, partial | *Escherichia coli* | *gad*A | 52067.00 | 5.00 | 70 | Glutamate metabolic process | [[6](#_ENREF_6)] |
| 24 | CLPX_ECOLI | MULTISPECIES: ATP-dependent protease | *Escherichia coli* | *clp*X | 49677.00 | 5.10 | 234 | Stress response | [[6](#_ENREF_6)] |
| 24 | E8J8T5_ECO57 | ATP-dependent protease ATP-binding subunit HslU | *Escherichia coli* | *hsl*U | 49677 | 5.11 | 234 | Stress response | [[1](#_ENREF_1)] |
| 25 | ALF_ECOLI | MULTISPECIES: Fructose-bisphosphate aldolase | *Escherichia coli* | *fba*A | 39351.00 | 5.50 | 100 | Glycolysis | [[6](#_ENREF_6)] |
| 25 | E8J5L7_ECO57 | Fructose-bisphosphate aldolase | *Escherichia coli* | *fba*A | 39351 | 5.47 | 100 | Glycolysis | [[1](#_ENREF_1)] |
| 27 | E8J111_ECO57 | Carbamoyl phosphate synthase small subunit | *Escherichia coli* | *car*A | 41633 | 5.91 | 84 | de novo' UMP biosynthesis | [[1](#_ENREF_1)] |
| 28 | S0WUT5_ECOLX | 6-phosphogluconate dehydrogenase, decarboxylating | *Escherichia coli* | *gnd* | 51547.00 | 5.00 | 73 | Pentose-phosphate shunt | [[8](#_ENREF_8)] |
| 28 | S1MDD6_ECOLX | 6-phosphogluconate dehydrogenase, decarboxylating | *Escherichia coli* | *gnd* | 51606.00 | 5.00 | 196 | Pentose-phosphate shunt | [[9](#_ENREF_9)] |
| 28 | 6PGD_ECOLI | 6-phosphogluconate dehydrogenase (decarboxylating) | *Escherichia coli* | *gnd* | 51563 | 4.90 | 164 | Pentose-phosphate shunt | [[6](#_ENREF_6)] |
| 30 | DCEA_ECOLI | Glutamate decarboxylase, partial | *Escherichia coli* | *gad*A | 52067.00 | 5.00 | 160 | Glutamate metabolic process | [[6](#_ENREF_6)] |
| 30 | DCEA_ECOLI | MULTISPECIES: Glutamate decarboxylase | *Escherichia coli* | *gad*A | 53221 | 5.10 | 159 | Glutamate metabolic process | [[6](#_ENREF_6)] |
| 30 | DCEA_ECOLI | Glutamate decarboxylase A subunit | *Escherichia coli* | *gad*A | 53221 | 5.10 | 159 | Glutamate metabolic process | [[6](#_ENREF_6)] |
| 31 | GLNA_ECOLI | MULTISPECIES: Glutamine synthetase | *Escherichia coli* | *gln*A | 52099.00 | 5.20 | 123 | Glutamine biosynthesis | [[6](#_ENREF_6)] |
| 31 | E8J8L6_ECO57 | Glutamine synthetase | *Escherichia coli* | *gln*A | 52099 | 5.16 | 123 | Glutamine biosynthesis | [[1](#_ENREF_1)] |
| 31 | GLNA_ECOLI | MULTISPECIES: Glutamine synthetase | *Escherichia coli* | *gln*A | 52099.00 | 5.20 | 123 | Glutamine biosynthesis | [[6](#_ENREF_6)] |
| 31 | E8J8L6_ECO57 | Glutamine synthetase | *Escherichia coli* | *gln*A | 52099 | 5.16 | 123 | Glutamine biosynthesis | [[1](#_ENREF_1)] |
| 34 | ASPA_ECOLI | MULTISPECIES: Aspartate ammonia-lyase | *Escherichia coli* | *asp*A | 29699.00 | 6.10 | 75 | Tricarboxylic acid cycle | [[6](#_ENREF_6)] |
| 37 | DCEA_ECOLI | MULTISPECIES: Glutamate decarboxylase | *Escherichia coli* | *gad*A | 53221.00 | 5.10 | 88 | Glutamate metabolic process | [[6](#_ENREF_6)] |
| 37 | DCEA_ECOLI | Glutamate decarboxylase A subunit | *Escherichia coli* | *gad*A | 53221 | 5.10 | 88 | Glutamate metabolic process | [[6](#_ENREF_6)] |
| 37 | DCEA_ECOLI | Glutamate decarboxylase subunit alpha, partial | *Escherichia coli* | *gad*A | 43530.00 | 5.30 | 72 | Glutamate metabolic process | [[6](#_ENREF_6)] |
| 38 | IMDH_ECOLI | MULTISPECIES: Inosine 5'-monophosphate dehydrogenase | *Escherichia coli* | *gua*B | 54016.00 | 6.00 | 189 | GMP biosynthesis | [[6](#_ENREF_6)] |
| 38 | A0A084ZCR5_ECOLX | Inosine-5'-monophosphate dehydrogenase | *Escherichia coli* | *gua*B | 52248 | 6.00 | 188 |  | [[4](#_ENREF_4)] |
| 39 | KPYK1_ECOLI | MULTISPECIES: Pyruvate kinase | *Escherichia coli* | *pyk*F | 51039.00 | 5.70 | 278 | Glycolysis | [[10](#_ENREF_10)] |
| 39 | E8J005_ECOLX | Pyruvate kinase | *Escherichia coli* | *pyk*F | 51039 | 5.71 | 278 | Glycolysis | [[1](#_ENREF_1)] |
| 40 | SYS_ECOLI | MULTISPECIES: Seryl-tRNA synthetase | *Escherichia coli* | *ser*S | 48669.00 | 5.20 | 122 | Selenocysteine biosynthesis | [[6](#_ENREF_6)] |
| 40 | E8JCH4_ECO57 | Seryl-tRNA synthetase | *Escherichia coli* | *ser*S | 48669 | 5.23 | 122 | Selenocysteine biosynthesis | [[1](#_ENREF_1)] |
| 41 | KPYK1_ECOLI | MULTISPECIES: Pyruvate kinase | *Escherichia coli* | *pyk*F | 51039.00 | 5.70 | 291 | Glycolysis | [[10](#_ENREF_10)] |
| 41 | E8J1S5_ECO57 | Pyruvate kinase | *Escherichia coli* | *pyk*F | 51039 | 5.71 | 291 | Glycolysis | [[1](#_ENREF_1)] |
| 42 | Q8VP34_ECOLX | MULTISPECIES: Dihydrolipoamide dehydrogenase | *Escherichia coli* | *ldp*A | 50942.00 | 5.80 | 100 | Oxidoreductase activity | [[7](#_ENREF_7)] |
| 42 | E8J192_ECO57 | Dihydrolipoamide dehydrogenase | *Escherichia coli* | *lpd*A | 50942 | 5.75 | 100 | Cell redox homeostasis | [[1](#_ENREF_1)] |
| 42 | A0A084Z371_ECOLX | Dihydrolipoamide dehydrogenase/dihydrolipoamide dehydrogenase of pyruvate dehydrogenase complex | *Escherichia coli* | *ldp*A | 50942 | 5.75 | 100 |  | [[4](#_ENREF_4)] |
| 44 | A0A066R858_ECOLX | Hypothetical protein | *Escherichia coli* |  | 13353.00 | 4.60 | 65 |  | [[11](#_ENREF_11)] |
| 45 | E8J134_ECO57 | Peptidyl-prolyl cis-trans isomerase SurA | *Escherichia coli* | *sur*A | 47235.00 | 6.40 | 93 | Chaperone | [[1](#_ENREF_1)] |
| 45 | PPIA_ECOLI | Peptidylprolyl isomerase | *Escherichia coli* | *ppi*A | 47195 | 6.30 | 92 | Protein folding | [[10](#_ENREF_10)] |
| 45 | PPIA_ECOLI | MULTISPECIES: Peptidyl-prolyl cis-trans isomerase | *Escherichia coli* | *ppi*A | 47254 | 6.53 | 90 | Protein folding | [[10](#_ENREF_10)] |
| 48 | GLMU_ECOLI | MULTISPECIES: Bifunctional N-acetylglucosamine-1-phosphate uridyltransferase/glucosamine-1-phosphate acetyltransferase | *Escherichia coli* | *glm*U | 48844.00 | 6.10 | 74 | Cell morphogenesis | [[6](#_ENREF_6)] |
| 48 | GLMU_ECOLI | MULTISPECIES: Bifunctional N-acetylglucosamine-1-phosphate uridyltransferase/glucosamine-1-phosphate acetyltransferase | *Escherichia coli* | *glm*U | 48844.00 | 6.10 | 71 | Cell morphogenesis | [[6](#_ENREF_6)] |
| 49 | EFTU1_ECOLI | MULTISPECIES: Elongation factor Tu, partial | *Escherichia coli* | *tuf*A | 41636.00 | 5.00 | 256 | Antibiotic response | [[6](#_ENREF_6)] |
| 53 | DCEA_ECOL6 | Glutamate decarboxylase alpha | *Escherichia coli* | *gad*A | 53221.00 | 5.10 | 102 | Glutamate metabolic process | [[12](#_ENREF_12)] |
| 53 | DCEB_ECO57 | Glutamate decarboxylase beta | *Escherichia coli* | *gad*A | 53204 | 5.17 | 101 | Glutamate metabolic process | [[13](#_ENREF_13)] |
| 53 | DCEB_ECO57 | Glutamate decarboxylase beta | *Escherichia coli* | *gad*A | 53204.00 | 5.20 | 159 | Glutamate metabolic process | [[13](#_ENREF_13)] |
| 53 | DCEA_ECOL6 | Glutamate decarboxylase alpha | *Escherichia coli* | *gad*A | 53221 | 5.10 | 146 | Glutamate metabolic process | [[12](#_ENREF_12)] |
| 54 | SYS_ECO24 | Serine--tRNA ligase | *Escherichia coli* | *ser*S | 48669.00 | 5.20 | 107 | Selenocysteine biosynthesis | [[14](#_ENREF_14)] |
| 55 | RPOB_ECO24 | DNA-directed RNA polymerase subunit beta | *Escherichia coli* | *rpo*B | 150937.00 | 5.00 | 277 | Transcription | [[14](#_ENREF_14)] |
| 56 | SYL_ECO55 | Leucine--tRNA ligase | *Escherichia coli* | *leu*S | 97801.00 | 5.00 | 232 | Leucine-tRNA ligase activity | [[15](#_ENREF_15)] |
| 57 | SYK2_ECO57 | Lysine--tRNA ligase, heat inducible | *Escherichia coli* | *lys*U | 57847.00 | 5.00 | 248 | Lysine-tRNA editing activity | [[13](#_ENREF_13)] |
| 57 | SYK1_ECO57 | Lysine--tRNA ligase | *Escherichia coli* | *lys*S | 57652 | 4.97 | 64 | Lysine-tRNA editing activity | [[13](#_ENREF_13)] |
| 58 | EFG_ECO24 | Elongation factor G | *Escherichia coli* | *fus*A | 77704.00 | 5.10 | 195 | Translation elongation factor activity | [[14](#_ENREF_14)] |
| 59 | EFG_ECO24 | Elongation factor G | *Escherichia coli* | *fus*A | 77704.00 | 5.10 | 281 | Translation elongation factor activity | [[14](#_ENREF_14)] |
| 60 | ODP1_ECO57 | Pyruvate dehydrogenase E1 component | *Escherichia coli* | *ace*E | 99948.00 | 5.40 | 266 | Glycolysis | [[13](#_ENREF_13)] |
| 63 | PFLB_ECOLI | Formate acetyltransferase 1 | *Escherichia coli* | *pfl*B | 85588.00 | 5.60 | 304 | Anaerobic respiration | [[6](#_ENREF_6)] |
| 63 | PFLB_ECOLI | Formate acetyltransferase 1 | *Escherichia coli* | *pfl*B | 85588.00 | 5.60 | 342 | Anaerobic respiration | [[6](#_ENREF_6)] |
| 64 | CLPB_ECO57 | Chaperone protein ClpB | *Escherichia coli* | *clp*B | 95697.00 | 5.30 | 376 | Heat response | [[13](#_ENREF_13)] |
| 64 | PFLB_ECOLI | Formate acetyltransferase 1 | *Escherichia coli* | *pfl*B | 85588 | 5.62 | 154 | Anaerobic respiration | [[6](#_ENREF_6)] |
| 65 | CLPB_ECO57 | Chaperone protein ClpB | *Escherichia coli* | *clp*B | 95697.00 | 5.30 | 331 | Heat response | [[13](#_ENREF_13)] |
| 65 | TKT1_ECOLI | Transketolase 1 | *Escherichia coli* | *tkt*A | 72451 | 5.34 | 63 | Pentose-phosphate shunt | [[6](#_ENREF_6)] |
| 66 | LDCI_ECO57 | Lysine decarboxylase, inducible | *Escherichia coli* | *cad*A | 81607.00 | 5.90 | 150 | Cellular amino acid metabolic process | [[13](#_ENREF_13)] |
| 66 | DCOS_ECOLI | Ornithine decarboxylase, inducible | *Escherichia coli* | *spe*F | 83390 | 5.52 | 81 | Spermidine biosynthesis | [[6](#_ENREF_6)] |
| 67 | PFLB_ECOLI | Formate acetyltransferase 1 | *Escherichia coli* | *pfl*B | 85588.00 | 5.60 | 330 | Anaerobic respiration | [[6](#_ENREF_6)] |
| 68 | PFLB_ECOLI | Formate acetyltransferase 1 | *Escherichia coli* | *pfl*B | 85588.00 | 5.60 | 328 | Anaerobic respiration | [[6](#_ENREF_6)] |
| 68 | CLPB_ECO57 | Chaperone protein ClpB | *Escherichia coli* | *clp*B | 95697 | 5.25 | 64 | Heat response | [[13](#_ENREF_13)] |
| 69 | PFLB_ECOLI | Formate acetyltransferase 1 | *Escherichia coli* | *pfl*B | 85588.00 | 5.60 | 352 | Anaerobic respiration | [[6](#_ENREF_6)] |
| 70 | G3P1_ECO57 | Glyceraldehyde-3-phosphate dehydrogenase A | *Escherichia coli* | *gap*A | 35681.00 | 6.70 | 362 | Glycolysis | [[13](#_ENREF_13)] |
| 71 | G3P1_ECO57 | Glyceraldehyde-3-phosphate dehydrogenase A | *Escherichia coli* | *gap*A | 35681.00 | 6.70 | 272 | Glycolysis | [[13](#_ENREF_13)] |
| 72 | G3P1_ECO57 | Glyceraldehyde-3-phosphate dehydrogenase A | *Escherichia coli* | *gap*A | 35681.00 | 6.70 | 241 | Glycolysis | [[13](#_ENREF_13)] |
| 73 | CBPA_ECO24 | Curved DNA-binding protein | *Escherichia coli* | *cbp*A | 34404.00 | 6.40 | 84 | Protein folding | [[14](#_ENREF_14)] |
| 74 | RS2_ECO27 | 30S ribosomal protein S2 | *Escherichia coli* | *rps*B | 26784.00 | 6.70 | 175 | Translation | [[16](#_ENREF_16)] |
| 75 | TPIS_ECO24 | Triosephosphate isomerase | *Escherichia coli* | *tpi*A | 27126.00 | 5.60 | 170 | Glycolysis | [[14](#_ENREF_14)] |
| 76 | KAD_ECO24 | Adenylate kinase | *Escherichia coli* | *adk* | 23628.00 | 5.40 | 157 | Adenylate kinase activity | [[14](#_ENREF_14)] |
| 77 | DEOC_ECO24 | Deoxyribose-phosphate aldolase | *Escherichia coli* | *deo*C | 27958.00 | 5.40 | 199 | Carbohydrate catabolic process | [[14](#_ENREF_14)] |
| 78 | PGK_ECO24 | Phosphoglycerate kinase | *Escherichia coli* | *pgk* | 41264.00 | 4.90 | 320 | Glycolysis | [[14](#_ENREF_14)] |
| 79 | EFTS_ECO24 | Elongation factor Ts | *Escherichia coli* | *tsf* | 30518.00 | 5.10 | 151 | Translation elongation factor activity | [[14](#_ENREF_14)] |
| 80 | KPRS_ECO57 | Ribose-phosphate pyrophosphokinase | *Escherichia coli* | *prs* | 34425.00 | 5.10 | 168 | Kinase activity | [[13](#_ENREF_13)] |
| 81 | YGGE_ECO57 | Uncharacterized protein YggE | *Escherichia coli* | *ygg*E | 26619.00 | 6.10 | 124 |  | [[13](#_ENREF_13)] |
| 82 | TALB_ECOL6 | Transaldolase B | *Escherichia coli* | *tal*B | 35354.00 | 5.00 | 218 | Pentose-phosphate shunt | [[12](#_ENREF_12)] |
| 83 | GSHB_ECO57 | Glutathione synthetase | *Escherichia coli* | *gsh*B | 35638.00 | 5.00 | 103 | Glutathione synthase activity | [[13](#_ENREF_13)] |
| 83 | TALB_ECO57 | Transaldolase B | *Escherichia coli* | *tal*B | 35368 | 4.96 | 65 | Pentose-phosphate shunt | [[13](#_ENREF_13)] |
| 85 | YEAD_ECOLI | Putative glucose-6-phosphate 1-epimerase | *Escherichia coli* | *yea*D | 32874.00 | 5.90 | 72 | Cellular response to DNA damage stimulus | [[10](#_ENREF_10)] |
| 86 | MDH_ECO24 | Malate dehydrogenase | *Escherichia coli* | *mdh* | 32488.00 | 5.50 | 186 | Tricarboxylic acid cycle | [[14](#_ENREF_14)] |
| 87 | PFKA_ECO57 | 6-phosphofructokinase isozyme 1 | *Escherichia coli* | *pfk*A | 35162.00 | 5.40 | 215 | Glycolysis | [[13](#_ENREF_13)] |
| 87 | PFKA_ECO24 | 6-phosphofructokinase | *Escherichia coli* | *pfk*A | 35162 | 5.38 | 215 | Glycolysis | [[14](#_ENREF_14)] |
| 88 | FABI_ECO57 | Enoyl-[acyl-carrier-protein] reductase [NADH] FabI | *Escherichia coli* | *fab*I | 28074.00 | 5.50 | 78 | Antibiotic response | [[13](#_ENREF_13)] |
| 89 | FABI_ECO57 | Enoyl-[acyl-carrier-protein] reductase [NADH] FabI | *Escherichia coli* | *fab*I | 28074.00 | 5.50 | 168 | Antibiotic response | [[13](#_ENREF_13)] |
| 90 | GPMA_ECO27 | 2,3-bisphosphoglycerate-dependent phosphoglycerate mutase | *Escherichia coli* | *gpm*A | 28539.00 | 5.80 | 123 | Glycolysis | [[16](#_ENREF_16)] |
| 91 | UDP_ECOLI | Uridine phosphorylase | *Escherichia coli* | *udp* | 27313.00 | 5.80 | 168 | Cellular response to DNA damage stimulus | [[10](#_ENREF_10)] |
| 92 | DKGA_ECOLI | 2,5-diketo-D-gluconic acid reductase A | *Escherichia coli* | *dkg*A | 31147.00 | 6.00 | 107 | L-ascorbic acid biosynthesis | [[6](#_ENREF_6)] |
| 93 | EFTU1_ECO24 | Elongation factor Tu 1 | *Escherichia coli* | *tuf*1 | 43427.00 | 5.20 | 128 | Translation elongation factor activity | [[14](#_ENREF_14)] |
| 94 | KDSA_ECO24 | 2-dehydro-3-deoxyphosphooctonate aldolase | *Escherichia coli* | *kds*A | 31041.00 | 6.40 | 170 | keto-3-deoxy-D-manno-octulosonic acid biosynthesis | [[14](#_ENREF_14)] |
| 95 | DAPA_ECO24 | 4-hydroxy-tetrahydrodipicolinate synthase | *Escherichia coli* | *dap*A | 31549.00 | 6.00 | 101 | Amine-lyase activity | [[14](#_ENREF_14)] |
| 97 | GRPE_ECO24 | Protein GrpE | *Escherichia coli* | *grp*E | 21727.00 | 4.50 | 153 | Stress response | [[14](#_ENREF_14)] |
| 98 | G3P1_ECO57 | Glyceraldehyde-3-phosphate dehydrogenase A | *Escherichia coli* | *gap*A | 35681.00 | 6.70 | 237 | Glycolysis | [[13](#_ENREF_13)] |
| 100 | G3P3_ECO57 | Glyceraldehyde-3-phosphate dehydrogenase C | *Escherichia coli* | *gap*C | 35912.00 | 6.00 | 94 | Glycolysis | [[13](#_ENREF_13)] |
| 102 | ENO_ECO24 | Enolase | *Escherichia coli* | *eno* | 45683.00 | 5.20 | 283 | Glycolysis | [[13](#_ENREF_13)] |
| 106 | YBBN_ECOLI | Uncharacterized protein YbbN | *Escherichia coli* | *ybb*N | 31885.00 | 4.30 | 152 | Chaperone | [[6](#_ENREF_6)] |
| 109 | PFKA_ECO57 | 6-phosphofructokinase isozyme 1 | *Escherichia coli* | *pfk*A | 35162.00 | 5.40 | 118 | Glycolysis | [[13](#_ENREF_13)] |
| 109 | PFKA_ECO24 | 6-phosphofructokinase | *Escherichia coli* | *pfk*A | 35162 | 5.38 | 118 | Glycolysis | [[14](#_ENREF_14)] |
| 111 | CYSK_ECO57 | Cysteine synthase A | *Escherichia coli* | *cys*K | 34525.00 | 5.80 | 183 | Cysteine biosynthesis | [[13](#_ENREF_13)] |
| 112 | TALA_ECO57 | Transaldolase A | *Escherichia coli* | *tal*A | 35865.00 | 5.90 | 127 | Pentose-phosphate shunt | [[13](#_ENREF_13)] |
| 113 | PDXJ_ECOL6 | Pyridoxine 5'-phosphate synthase | *Escherichia coli* | *pdx*J | 26630.00 | 5.40 | 113 | Pyridoxine biosynthesis | [[12](#_ENREF_12)] |
| 117 | PFKB_ECOLI | 6-phosphofructokinase isozyme 2 | *Escherichia coli* | *pfk*B | 32664.00 | 5.10 | 96 | Glycolysis | [[6](#_ENREF_6)] |
| 117 | SYFA_ECO24 | Phenylalanine--tRNA ligase alpha subunit | *Escherichia coli* | *phe*S | 36866 | 5.77 | 78 | Phenylalanyl-tRNA aminoacylation | [[14](#_ENREF_14)] |
| 120 | KCY_ECO24 | Cytidylate kinase | *Escherichia coli* | *cmk* | 24788.00 | 5.50 | 111 | Pyrimidine nucleotide metabolic process | [[14](#_ENREF_14)] |
| 121 | YBIS_ECO57 | Probable L,D-transpeptidase YbiS | *Escherichia coli* | *ybi*S | 33418.00 | 6.00 | 114 | Cell wall organization | [[13](#_ENREF_13)] |
| 128 | EFTU1_ECO24 | Elongation factor Tu 1 | *Escherichia coli* | *tuf*1 | 43427.00 | 5.20 | 237 | Translation elongation factor activity | [[14](#_ENREF_14)] |
| 130 | NFNB_ECOLI | Oxygen-insensitive NAD(P)H nitroreductase | *Escherichia coli* | *nfn*B | 23947.00 | 5.80 | 91 | NAD(P)H nitroreductase activity | [[6](#_ENREF_6)] |
| 138 | FKBA_ECO57 | FKBP-type peptidyl-prolyl cis-trans isomerase FkpA | *Escherichia coli* | *pkp*A | 28894.00 | 9.00 | 131 | Protein folding | [[13](#_ENREF_13)] |
| 139 | PFKA_ECO57 | 6-phosphofructokinase isozyme 1 | *Escherichia coli* | *pfk*A | 35162.00 | 5.40 | 95 | Glycolysis | [[13](#_ENREF_13)] |
| 139 | PFKA_ECOL6 | 6-phosphofructokinase | *Escherichia coli* | *pfk*A | 35176 | 5.39 | 95 | Glycolysis | [[12](#_ENREF_12)] |
| 145 | TPIS_ECO24 | Triosephosphate isomerase | *Escherichia coli* | *tpi*A | 27126.00 | 5.60 | 68 | Glycolysis | [[14](#_ENREF_14)] |
| 146 | FABI_ECO57 | Enoyl-[acyl-carrier-protein] reductase [NADH] FabI | *Escherichia coli* | *fab*I | 28074.00 | 5.50 | 155 | Antibiotic response | [[13](#_ENREF_13)] |
| 147 | DAPD_ECO27 | 2,3,4,5-tetrahydropyridine-2,6-dicarboxylate N-succinyltransferase | *Escherichia coli* | *dap*D | 30044.00 | 5.70 | 130 | Diaminopimelate biosynthesis | [[16](#_ENREF_16)] |
| 159 | CARB_ECOL6 | Carbamoyl-phosphate synthase large chain | *Escherichia coli* | *car*B | 118615.00 | 5.10 | 234 | de novo' UMP biosynthesis | [[12](#_ENREF_12)] |

Bibliography:

[1] Rump LV, Strain EA, Cao G, Allard MW, Fischer M, Brown EW, et al. Draft genome sequences of six *Escherichia coli* isolates from the stepwise model of emergence of *Escherichia coli* O157:H7. Journal of bacteriology. 2011;193:2058-9.

[2] Geddes RD, Wang X, Yomano LP, Miller EN, Zheng H, Shanmugam KT, et al. Polyamine transporters and polyamines increase furfural tolerance during xylose fermentation with ethanologenic *Escherichia coli* strain LY180. Applied and environmental microbiology. 2014;80:5955-64.

[3] Feldgarden M, Nielsen KL, Frimodt-Moller N, Andersen PS, Walker B, Young SK, et al. The Genome Sequence of *Escherichia coli* KTE159. Submitted (JAN-2013) to the EMBL/GenBank/DDBJ databases Cited for: NUCLEOTIDE SEQUENCE Strain: KTE159.

[4] Plunkett GI, Neeno-Eckwall EC, Glasner JD, Perna NT. ATOL: Assembling a taxonomically balanced genome-scale reconstruction of the evolutionary history of the *Enterobacteriaceae*. Submitted (MAY-2014) to the EMBL/GenBank/DDBJ databases Cited for: NUCLEOTIDE SEQUENCE Strain: ATCC 11775.

[5] Suzuki H, Richards V, Lefebure T, Pavinski Bitar P, Lang P, Stanhope M. Submitted (APR-2010) to the EMBL/GenBank/DDBJ databases Cited for: NUCLEOTIDE SEQUENCE Strain: ECC-1470.

[6] Blattner FR, Plunkett G, 3rd, Bloch CA, Perna NT, Burland V, Riley M, et al. The complete genome sequence of *Escherichia coli* K-12. Science. 1997;277:1453-62.

[7] Huang SH, Chen YH, Kong G, Chen SH, Besemer J, Borodovsky M, et al. A novel genetic island of meningitic *Escherichia coli* K1 containing the ibeA invasion gene (GimA): functional annotation and carbon-source-regulated invasion of human brain microvascular endothelial cells. Functional & integrative genomics. 2001;1:312-22.

[8] Feldgarden M, Nielsen KL, Frimodt-Moller N, Andersen PS, Walker B, Young SK, et al. The Genome Sequence of *Escherichia coli* KTE31. Submitted (JAN-2013) to the EMBL/GenBank/DDBJ databases Cited for: NUCLEOTIDE SEQUENCE Strain: KTE31.

[9] Feldgarden M, Nielsen KL, Frimodt-Moller N, Andersen PS, Walker B, Young SK, et al. The Genome Sequence of *Escherichia coli* KTE172. Submitted (JAN-2013) to the EMBL/GenBank/DDBJ databases Cited for: NUCLEOTIDE SEQUENCE Strain: KTE172.

[10] Hayashi K, Morooka N, Yamamoto Y, Fujita K, Isono K, Choi S, et al. Highly accurate genome sequences of *Escherichia coli* K-12 strains MG1655 and W3110. Molecular systems biology. 2006;2:2006 0007.

[11] Leonard SR, Lacher DW. *Escherichia coli* non-O157 genome sequencing. Submitted (MAR-2014) to the EMBL/GenBank/DDBJ databases Cited for: NUCLEOTIDE SEQUENCE Strain: PMK-5.

[12] Welch RA, Burland V, Plunkett G, 3rd, Redford P, Roesch P, Rasko D, et al. Extensive mosaic structure revealed by the complete genome sequence of uropathogenic *Escherichia coli*. Proceedings of the National Academy of Sciences of the United States of America. 2002;99:17020-4.

[13] Perna NT, Plunkett G, 3rd, Burland V, Mau B, Glasner JD, Rose DJ, et al. Genome sequence of enterohaemorrhagic *Escherichia coli* O157:H7. Nature. 2001;409:529-33.

[14] Rasko DA, Rosovitz MJ, Myers GS, Mongodin EF, Fricke WF, Gajer P, et al. The pangenome structure of *Escherichia coli*: comparative genomic analysis of *E. coli* commensal and pathogenic isolates. Journal of bacteriology. 2008;190:6881-93.

[15] Touchon M, Hoede C, Tenaillon O, Barbe V, Baeriswyl S, Bidet P, et al. Organised genome dynamics in the *Escherichia coli* species results in highly diverse adaptive paths. PLoS genetics. 2009;5:e1000344.

[16] Iguchi A, Thomson NR, Ogura Y, Saunders D, Ooka T, Henderson IR, et al. Complete genome sequence and comparative genome analysis of enteropathogenic *Escherichia coli* O127:H6 strain E2348/69. Journal of bacteriology. 2009;191:347-54.
