# Supplementary material for: Multiomics Assessment of Gene Expression in a Clinical Strain of CTX-M-15-Producing ST131 Escherichia coli
Source: Front Microbiol. 2019 May 3;10:831. doi: 10.3389/fmicb.2019.00831 (PMC6509150; doi:10.3389/fmicb.2019.00831)
Supplement: TABLE S7 — Identification of protein spots from 2DE gels of extracellular extracts of ESBL-producing E. coli isolate C999 based on MALDI-TOF/MS sequencing results. [file Table_7.DOCX]

**Supplementary Table S7.** Identification of protein spots from extracellular extraction of ESBL-producing *E. coli* isolate C999 using 2-DE gels and MALDI-TOF sequencing results.

| **Spot** | **Accession Number** | **Protein** | **Serotype** | **Gene name** | **Protein MW** | **Protein pI** | **Mascot Score** | **Biological Process** | **Reference** |
| --- | --- | --- | --- | --- | --- | --- | --- | --- | --- |
| 1 | DNAK_ECO24 | Chaperone protein DnaK | *Escherichia coli* | *dna*K | 69130.00 | 4.70 | 306 | Stress response | [[1](#_ENREF_1)] |
| 1 | DNAK_ECOLI | MULTISPECIES: molecular chaperone DnaK | *Escherichia coli* | *dna*K | 69146.00 | 4.70 | 143 | Cellular response to unfolded protein | [[2](#_ENREF_2)] |
| 4 | PNP_ECO24 | Polyribonucleotide nucleotidyltransferase | *Escherichia coli* | *pnp* | 77111.00 | 4.90 | 81 | mRNA catabolic process | [[1](#_ENREF_1)] |
| 5 | EFG_ECO24 | Elongation factor G | *Escherichia coli* | *fus*A | 77704.00 | 5.10 | 86 | Translation | [[1](#_ENREF_1)] |
| 7 | OMPC_ECO57 | Outer membrane protein C | *Escherichia coli* | *omp*C | 40483.00 | 4.40 | 82 | Ion transport | [[3](#_ENREF_3)] |
| 7 | S0WHV4_ECOLX | outer membrane protein C | *Escherichia coli* | *omp*C | 41150.00 | 4.50 | 117 | Ion transport | [[4](#_ENREF_4)] |
| 7 | A1ED45_ECOLX | membrane protein | *Escherichia coli* | *omp*C | 41209 | 4.47 | 102 | Ion transport | [[5](#_ENREF_5)] |
| 9 | PT1_ECOLI | Phosphoenolpyruvate-protein phosphotransferase | *Escherichia coli* | *pts*I | 63750.00 | 4.60 | 85 | Kinase activity | [[6](#_ENREF_6)] |
| 10 | HTPG_ECO24 | Chaperone protein HtpG | *Escherichia coli* | *htp*G | 71404.00 | 4.90 | 100 | Stress response | [[1](#_ENREF_1)] |
| 12 | OMPA_ECO57 | Outer membrane protein A | *Escherichia coli* | *omp*A | 37292.00 | 6.00 | 144 | Conjugation | [[3](#_ENREF_3)] |
| 12 | S1LQC0_ECOLX | outer membrane protein A | *Escherichia coli* | *omp*A | 37611.00 | 5.70 | 169 | Conjugation | [[7](#_ENREF_7)] |
| 13 | FLIC_ECOLI | Flagellin | *Escherichia coli* | *fli*C | 51265.00 | 4.30 | 79 | Bacterial-type flagellum-dependent cell motility | [[6](#_ENREF_6)] |
| 13 | FLIC_ECOLI | MULTISPECIES: flagellin | *Escherichia coli* | *fli*C | 36240.00 | 4.80 | 98 | Bacterial-type flagellum-dependent cell motility | [[6](#_ENREF_6)] |
| 13 | FLIC_ECOLI | MULTISPECIES: flagellin | *Escherichia coli* | *fli*C | 36240.00 | 4.80 | 181 | Bacterial-type flagellum-dependent cell motility | [[6](#_ENREF_6)] |
| 14 | PGK_ECO24 | Phosphoglycerate kinase | *Escherichia coli* | *pgk* | 41264.00 | 4.90 | 138 | Glycolysis | [[1](#_ENREF_1)] |
| 14 | PGK_ECOLI | MULTISPECIES: phosphoglycerate kinase | *Escherichia coli* | *pgk* | 40705.00 | 4.90 | 97 | Glycolysis | [[2](#_ENREF_2)] |
| 19 | FADL_ECOL6 | Long-chain fatty acid transport protein | *Escherichia coli* | *fad*L | 48650.00 | 4.70 | 54 | Lipid transport | [[8](#_ENREF_8)] |
| 22 | OMPA_ECO57 | Outer membrane protein A | *Escherichia coli* | *omp*A | 37292.00 | 6.00 | 99 | Conjugation | [[3](#_ENREF_3)] |
| 22 | S1LQC0_ECOLX | outer membrane protein A | *Escherichia coli* | *omp*A | 37611.00 | 5.70 | 95 |  | [[7](#_ENREF_7)] |
| 26 | FLGL_ECOLI | Flagellar hook-associated protein 3 | *Escherichia coli* | *flg*L | 34260.00 | 4.50 | 73 | Bacterial-type flagellum-dependent cell motility | [[6](#_ENREF_6)] |
| 27 | OMPA_ECO57 | Outer membrane protein A | *Escherichia coli* | *omp*A | 37292.00 | 6.00 | 144 | Conjugation | [[3](#_ENREF_3)] |
| 27 | S1LQC0_ECOLX | outer membrane protein A | *Escherichia coli* | *omp*A | 37611.00 | 5.70 | 127 | Conjugation | [[7](#_ENREF_7)] |
| 29 | CYSB_ECOLI | MULTISPECIES: CysB family transcriptional regulator | *Escherichia coli* | *cys*B | 36026.00 | 7.00 | 65 | Cysteine biosynthesis | [[6](#_ENREF_6)] |
| 32 | TPIS_ECO24 | Triosephosphate isomerase | *Escherichia coli* | *tpi*A | 27126.00 | 5.60 | 73 | Glycolysis | [[1](#_ENREF_1)] |
| 44 | DLDH_ECO57 | Dihydrolipoyl dehydrogenase | *Escherichia coli* | *lpd*A | 50942.00 | 5.80 | 103 | Glycolysis | [[3](#_ENREF_3)] |
| 46 | ACKA_ECOLI | MULTISPECIES: acetate kinase | *Escherichia coli* | *ack*A | 43601.00 | 5.80 | 73 | Acetate biosynthesis | [[2](#_ENREF_2)] |
| 46 | ACKA_ECOLI | MULTISPECIES: acetate kinase | *Escherichia coli* | *ack*A | 43601.00 | 5.80 | 96 | Acetate biosynthesis | [[2](#_ENREF_2)] |
| 47 | C6EC71_ECOBD | MULTISPECIES: transposase IS600, partial | *Escherichia coli* | *yis*1 | 20490.00 | 9.60 | 71 | Transposition | [[9](#_ENREF_9)] |
| 52 | HNS_ECOLI | MULTISPECIES: DNA-binding protein | *Escherichia coli* | *hns* | 7624.00 | 10.60 | 60 | Transcription | [[2](#_ENREF_2)] |
| 60 | OMPA_ECO57 | Outer membrane protein A | *Escherichia coli* | *omp*A | 37292.00 | 6.00 | 88 | Conjugation | [[3](#_ENREF_3)] |
| 62 | OMPA_ECO57 | Outer membrane protein A | *Escherichia coli* | *omp*A | 37292.00 | 6.00 | 92 | Conjugation | [[3](#_ENREF_3)] |
| 66 | CARB_ECOL6 | Carbamoyl-phosphate synthase large chain | *Escherichia coli* | *car*B | 118615.00 | 5.10 | 55 | de novo' UMP biosynthesis | [[8](#_ENREF_8)] |
| 69 | 6PGD_ECOLI | MULTISPECIES: 6-phosphogluconate dehydrogenase | *Escherichia coli* | *gnd* | 51547.00 | 4.90 | 69 | Pentose-phosphate shunt | [[2](#_ENREF_2)] |
| 69 | 6PGD_ECOLI | 6-phosphogluconate dehydrogenase, decarboxylating | *Escherichia coli* | *gnd* | 51563.00 | 4.90 | 70 | Pentose-phosphate shunt | [[2](#_ENREF_2)] |
| 72 | Q47328_ECOLX | MULTISPECIES: hypothetical protein | *Escherichia coli* |  | 11465.00 | 10.40 | 71 |  | [[10](#_ENREF_10)] |
| 73 | S1LKZ2_ECOLX | transposase | *Escherichia coli* |  | 36951.00 | 5.90 | 69 |  | [[7](#_ENREF_7)] |
| 73 | FADB_ECOSM | Fatty acid oxidation complex subunit alpha | *Escherichia coli* | *fad*B | 79829.00 | 5.50 | 19 | Fatty acid beta-oxidation | [[11](#_ENREF_11)] |
| 77 | MIND_ECO57 | Septum site-determining protein MinD | *Escherichia coli* | *min*D | 29710.00 | 5.10 | 28 | Barrier septum site selection | [[3](#_ENREF_3)] |
| 78 | KDSA_ECO24 | 2-dehydro-3-deoxyphosphooctonate aldolase | *Escherichia coli* | *kds*A | 31041.00 | 6.40 | 25 | Keto-3-deoxy-D-manno-octulosonic acid biosynthesis | [[1](#_ENREF_1)] |
| 78 | CARB_ECOLI | Carbamoyl-phosphate synthase large chain | *Escherichia coli* | *car*B | 118566.00 | 5.10 | 20 | de novo' UMP biosynthesis | [[6](#_ENREF_6)] |
| 79 | S1LFS2_ECOLX | deoxyribose-phosphate aldolase | *Escherichia coli* | *deo*C | 31953.00 | 5.30 | 28 | Carbon-carbon lyase activity | [[7](#_ENREF_7)] |
| 79 | MIND_ECO57 | Septum site-determining protein MinD | *Escherichia coli* | *min*D | 29710.00 | 5.10 | 28 | Barrier septum site selection | [[3](#_ENREF_3)] |
| 80 | MIND_ECO57 | Septum site-determining protein MinD | *Escherichia coli* | *min*D | 29710.00 | 5.10 | 24 | Barrier septum site selection | [[3](#_ENREF_3)] |
| 85 | HNS_ECOLI | MULTISPECIES: DNA-binding protein | *Escherichia coli* | *hns* | 7624.00 | 10.60 | 60 | Transcription | [[2](#_ENREF_2)] |
| 88 | CPXP_ECOL6 | Periplasmic protein CpxP | *Escherichia coli* | *cpx*P | 18953.00 | 6.40 | 20 |  | [[8](#_ENREF_8)] |
| 92 | EFTS_ECO24 | Elongation factor Ts | *Escherichia coli* | *tsf* | 30518.00 | 5.10 | 18 | Translation | [[1](#_ENREF_1)] |
| 93 | HNS_ECOLI | MULTISPECIES: DNA-binding protein | *Escherichia coli* | *hns* | 7624.00 | 10.60 | 60 | Transcription | [[2](#_ENREF_2)] |
| 99 | ACKA_ECOLI | MULTISPECIES: adenylate kinase | *Escherichia coli* | *ack*A | 23676.00 | 5.40 | 34 | Acetate biosynthesis | [[2](#_ENREF_2)] |
| 101 | ACKA_ECOLI | MULTISPECIES: adenylate kinase | *Escherichia coli* | *ack*A | 23676.00 | 5.40 | 34 | Acetate biosynthesis | [[2](#_ENREF_2)] |
| 102 | TALB_ECO57 | Transaldolase B | *Escherichia coli* | *tal*B | 35368.00 | 5.00 | 24 | Pentose-phosphate shunt | [[3](#_ENREF_3)] |
| 103 | TALB_ECO57 | Transaldolase B | *Escherichia coli* | *tal*B | 35368.00 | 5.00 | 23 | Pentose-phosphate shunt | [[3](#_ENREF_3)] |
| 104 | HNS_ECOLI | MULTISPECIES: DNA-binding protein | *Escherichia coli* | *hns* | 7624.00 | 10.60 | 60 | Transcription | [[2](#_ENREF_2)] |

Bibliography:

[1] Rasko DA, Rosovitz MJ, Myers GS, Mongodin EF, Fricke WF, Gajer P, et al. The pangenome structure of *Escherichia coli*: comparative genomic analysis of *E. coli* commensal and pathogenic isolates. Journal of bacteriology. 2008;190:6881-93.

[2] Blattner FR, Plunkett G, 3rd, Bloch CA, Perna NT, Burland V, Riley M, et al. The complete genome sequence of *Escherichia coli* K-12. Science. 1997;277:1453-62.

[3] Perna NT, Plunkett G, 3rd, Burland V, Mau B, Glasner JD, Rose DJ, et al. Genome sequence of enterohaemorrhagic *Escherichia coli* O157:H7. Nature. 2001;409:529-33.

[4] Feldgarden M, Nielsen KL, Frimodt-Moller N, Andersen PS, Walker B, Young SK, et al. The Genome Sequence of *Escherichia coli* KTE31. Submitted (JAN-2013) to the EMBL/GenBank/DDBJ databases Cited for: NUCLEOTIDE SEQUENCE Strain: KTE31.

[5] Garrett ME, Parker J, Stephens C. Draft Genome Sequences of 6 Multi-Drug Resistant Human Commensal *Escherichia coli* Isolates. Submitted (JUN-2014) to the EMBL/GenBank/DDBJ databases Cited for: NUCLEOTIDE SEQUENCE Strain: CS01.

[6] Hayashi K, Morooka N, Yamamoto Y, Fujita K, Isono K, Choi S, et al. Highly accurate genome sequences of *Escherichia coli* K-12 strains MG1655 and W3110. Molecular systems biology. 2006;2:2006 0007.

[7] Feldgarden M, Nielsen KL, Frimodt-Moller N, Andersen PS, Walker B, Young SK, et al. The Genome Sequence of *Escherichia coli* KTE159. Submitted (JAN-2013) to the EMBL/GenBank/DDBJ databases Cited for: NUCLEOTIDE SEQUENCE Strain: KTE159.

[8] Welch RA, Burland V, Plunkett G, 3rd, Redford P, Roesch P, Rasko D, et al. Extensive mosaic structure revealed by the complete genome sequence of uropathogenic *Escherichia coli*. Proceedings of the National Academy of Sciences of the United States of America. 2002;99:17020-4.

[9] Krempl PM, Mairhofer J, Eisenkolb M, Specht T, Leparc GG, Kreil DP, et al. Sequencing and gene expression analysis of *Escherichia coli* BL21(DE3). Submitted (MAR-2008) to the EMBL/GenBank/DDBJ databases Cited for: NUCLEOTIDE SEQUENCE Strain: BL21.

[10] Petit C, Rigg GP, Pazzani C, Smith A, Sieberth V, Stevens M, et al. Region 2 of the *Escherichia coli* K5 capsule gene cluster encoding proteins for the biosynthesis of the K5 polysaccharide. Molecular microbiology. 1995;17:611-20.

[11] Fricke WF, Wright MS, Lindell AH, Harkins DM, Baker-Austin C, Ravel J, et al. Insights into the environmental resistance gene pool from the genome sequence of the multidrug-resistant environmental isolate *Escherichia coli* SMS-3-5. Journal of bacteriology. 2008;190:6779-94.
