# Supplementary material for: Multiomics Assessment of Gene Expression in a Clinical Strain of CTX-M-15-Producing ST131 Escherichia coli
Source: Front Microbiol. 2019 May 3;10:831. doi: 10.3389/fmicb.2019.00831 (PMC6509150; doi:10.3389/fmicb.2019.00831)
Supplement: TABLE S8 — Identification of protein spots from 2DE gels of membrane extracts of ESBL-producing E. coli isolate C999 based on MALDI-TOF/MS sequencing results. [file Table_8.DOCX]

**Supplementary Table S8.** Identification of protein spots from membrane extraction of ESBL-producing *E. coli* isolate C999 using 2-DE gels and MALDI-TOF sequencing results.

| **Spot** | **Accession** | **Title** | **Species / Serotype** | **Gene name** | **Protein MW** | **Protein pI** | **Mascot Score** | **Biological Process** | **Reference** |
| --- | --- | --- | --- | --- | --- | --- | --- | --- | --- |
| 1 | RPOB_ECO24 | DNA-directed RNA polymerase subunit beta | *Escherichia coli* | *rpo*B | 150937.00 | 5.00 | 542 | Transcription | [[1](#_ENREF_1)] |
| 3 | RPOC_ECOK1 | DNA-directed RNA polymerase subunit beta' | *Escherichia coli* | *rpo*C | 155932.00 | 6.70 | 544 | Transcription | [[2](#_ENREF_2)] |
| 5 | LON_ECOL6 | Lon protease | *Escherichia coli* | *lon* | 87725.00 | 6.00 | 378 | Stress response | [[3](#_ENREF_3)] |
| 5 | LIVG_ECO57 | High-affinity branched-chain amino acid transport ATP-binding protein LivG | *Escherichia coli* | *liv*G | 28523 | 6.81 | 26 | Amino acid transport | [[4](#_ENREF_4)] |
| 5 | AAT_ECOLI | Aspartate aminotransferase | *Escherichia coli* | *asp*C | 43831 | 5.45 | 36 | L-phenylalanine biosynthesis | [[5](#_ENREF_5)] |
| 13 | TIG_ECOL5 | Trigger factor | *Escherichia coli* | *tig* | 48221.00 | 4.70 | 456 | Protein folding | [[6](#_ENREF_6)] |
| 14 | ATPB_ECO24 | ATP synthase subunit beta | *Escherichia coli* | *atp*D | 50351.00 | 4.80 | 344 | ATP hydrolysis | [[1](#_ENREF_1)] |
| 16 | NUSA_ECO57 | Transcription termination/antitermination protein NusA | *Escherichia coli* | *nus*A | 55008.00 | 4.40 | 200 | Transcription | [[4](#_ENREF_4)] |
| 16 | YCAQ_ECOLI | Uncharacterized protein YcaQ | *Escherichia coli* | *yca*Q | 47911 | 9.45 | 45 |  | [[7](#_ENREF_7)] |
| 16 | OPGG_ECO24 | Glucans biosynthesis protein G | *Escherichia coli* | *mdo*G | 57846 | 6.83 | 30 | Glucan biosynthesis | [[1](#_ENREF_1)] |
| 18 | PT1_ECOLI | Phosphoenolpyruvate-protein phosphotransferase | *Escherichia coli* | *pts*I | 63750.00 | 4.60 | 261 | Phosphoenolpyruvate-dependent sugar phosphotransferase system | [[7](#_ENREF_7)] |
| 18 | CH601_ECOK1 | 60 kDa chaperonin 1 | *Escherichia coli* | *gro*L1 | 57464 | 4.69 | 83 | protein refolding | [[2](#_ENREF_2)] |
| 18 | CH60_ECO24 | 60 kDa chaperonin | *Escherichia coli* | *gro*L | 57464 | 4.69 | 83 | protein refolding | [[1](#_ENREF_1)] |
| 19 | CH601_ECOK1 | 60 kDa chaperonin 1 | *Escherichia coli* | *gro*L1 | 57464.00 | 4.70 | 306 | protein refolding | [[2](#_ENREF_2)] |
| 19 | CH60_ECO24 | 60 kDa chaperonin | *Escherichia coli* | *gro*L | 57464 | 4.69 | 306 | protein refolding | [[1](#_ENREF_1)] |
| 20 | CH601_ECOK1 | 60 kDa chaperonin 1 | *Escherichia coli* | *gro*L1 | 57464.00 | 4.70 | 289 | protein refolding | [[2](#_ENREF_2)] |
| 20 | CH60_ECO24 | 60 kDa chaperonin | *Escherichia coli* | *gro*L | 57464 | 4.69 | 289 | protein refolding | [[1](#_ENREF_1)] |
| 26 | SECA_ECO27 | Protein translocase subunit SecA | *Escherichia coli* | *sec*A | 102111.00 | 5.40 | 272 | Intracellular protein transmembrane transport | [[8](#_ENREF_8)] |
| 27 | EFG_ECO24 | Elongation factor G | *Escherichia coli* | *fus*A | 77704.00 | 5.10 | 414 | GTP-dependent ribosomal translocation step | [[1](#_ENREF_1)] |
| 33 | CLPB_ECO57 | Chaperone protein ClpB | *Escherichia coli* | *clp*B | 95697.00 | 5.30 | 613 | Heat response | [[4](#_ENREF_4)] |
| 33 | SYL_ECO7I | Leucine--tRNA ligase | *Escherichia coli* | *leu*S | 97813 | 5.06 | 32 | Leucyl-tRNA aminoacylation | [[9](#_ENREF_9)] |
| 33 | UBIC_ECO27 | Chorismate--pyruvate lyase | *Escherichia coli* | *ubi*C | 18853 | 7.56 | 36 | Ubiquinone biosynthesis | [[8](#_ENREF_8)] |
| 34 | HTPG_ECO57 | Chaperone protein HtpG | *Escherichia coli* | *htp*G | 71378.00 | 5.00 | 354 | Stress response | [[4](#_ENREF_4)] |
| 36 | HTPG_ECO57 | Chaperone protein HtpG | *Escherichia coli* | *htp*G | 71378.00 | 5.00 | 325 | Stress response | [[4](#_ENREF_4)] |
| 37 | TKT1_ECOLI | Transketolase 1 | *Escherichia coli* | *tkt*A | 72451.00 | 5.30 | 189 | Pentose-phosphate shunt | [[5](#_ENREF_5)] |
| 37 | SECD_ECO57 | Protein translocase subunit SecD | *Escherichia coli* | *sec*D | 66648 | 9.13 | 32 | Intracellular protein transmembrane transport | [[4](#_ENREF_4)] |
| 39 | CLPB_ECO57 | Chaperone protein ClpB | *Escherichia coli* | *clp*B | 95697.00 | 5.30 | 477 | Heat response | [[4](#_ENREF_4)] |
| 39 | MENC_ECO45 | o-succinylbenzoate synthase | *Escherichia coli* | *men*C | 35713 | 4.78 | 53 | Menaquinone biosynthesis | [[9](#_ENREF_9)] |
| 41 | ODP1_ECO57 | Pyruvate dehydrogenase E1 component | *Escherichia coli* | *ace*E | 99948.00 | 5.40 | 368 | Glycolysis | [[4](#_ENREF_4)] |
| 41 | ELBH_ECOLX | Heat-labile enterotoxin B chain | *Escherichia coli* | *elt*B | 14189 | 9.35 | 34 | Pathogenesis | [[10](#_ENREF_10)] |
| 41 | TRAI2_ECOLX | Multifunctional conjugation protein TraI | *Escherichia coli* | *tra*I | 191564 | 5.59 | 42 | Conjugation | [[11](#_ENREF_11)] |
| 46 | CLPB_ECO57 | Chaperone protein ClpB | *Escherichia coli* | *clp*B | 95697.00 | 5.30 | 389 | Heat response | [[4](#_ENREF_4)] |
| 46 | MENC_ECO45 | o-succinylbenzoate synthase | *Escherichia coli* | *men*C | 35713 | 4.78 | 52 |  | [[9](#_ENREF_9)] |
| 48 | SYK2_ECO57 | Lysine--tRNA ligase, heat inducible | *Escherichia coli* | *lys*U | 57847.00 | 5.00 | 312 | Lysyl-tRNA aminoacylation | [[4](#_ENREF_4)] |
| 48 | SYK1_ECO57 | Lysine--tRNA ligase | *Escherichia coli* | *lys*A | 57652 | 4.97 | 79 | Lysyl-tRNA aminoacylation | [[4](#_ENREF_4)] |
| 51 | PTA_ECOLI | Phosphate acetyltransferase | *Escherichia coli* | *pta* | 77466.00 | 5.20 | 311 | Acetate biosynthesis | [[5](#_ENREF_5)] |
| 51 | ZAPE_ECO57 | Cell division protein ZapE | *Escherichia coli* | *zap*E | 43264 | 6.40 | 33 | Cell division | [[4](#_ENREF_4)] |
| 55 | RS1_ECO57 | 30S ribosomal protein S1 | *Escherichia coli* | *rps*A | 61235.00 | 4.70 | 293 | Translation | [[4](#_ENREF_4)] |
| 55 | DNAK_ECO24 | Chaperone protein DnaK | *Escherichia coli* | *dna*K | 69130 | 4.68 | 37 | Stress response | [[1](#_ENREF_1)] |
| 56 | DNAK_ECO24 | Chaperone protein DnaK | *Escherichia coli* | *dna*K | 69130.00 | 4.70 | 398 | Stress response | [[1](#_ENREF_1)] |
| 61 | PYRG_ECO24 | CTP synthase | *Escherichia coli* | *pyr*G | 60792.00 | 5.60 | 300 | de novo' CTP biosynthesis | [[1](#_ENREF_1)] |
| 64 | ETTA_ECO57 | Energy-dependent translational throttle protein EttA | *Escherichia coli* | *ett*A | 62518.00 | 5.30 | 240 | Translation | [[4](#_ENREF_4)] |
| 64 | HYCE_ECOLI | Formate hydrogenlyase subunit 5 | *Escherichia coli* | *hyc*E | 65395 | 6.15 | 131 | NADH dehydrogenase activity | [[7](#_ENREF_7)] |
| 64 | MIAA_ECO57 | tRNA dimethylallyltransferase | *Escherichia coli* | *mia*A | 35099 | 5.77 | 43 | tRNA processing | [[4](#_ENREF_4)] |
| 64 | SLT_ECO57 | Soluble lytic murein transglycosylase | *Escherichia coli* | *slt* | 73478 | 9.29 | 36 | Peptidoglycan metabolic process | [[4](#_ENREF_4)] |
| 65 | OPGG_ECO24 | Glucans biosynthesis protein G | *Escherichia coli* | *mdo*G | 57846.00 | 6.80 | 291 | Glucan biosynthesis | [[1](#_ENREF_1)] |
| 66 | YDJH_ECOLI | Uncharacterized sugar kinase YdjH | *Escherichia coli* | *ydj*H | 34556.00 | 4.70 | 34 | D-ribose metabolic process | [[7](#_ENREF_7)] |
| 66 | YBCJ_ECOLI | Uncharacterized protein YbcJ | *Escherichia coli* | *ybc*J | 7499.00 | 9.40 | 40 | RNA binding | [[7](#_ENREF_7)] |
| 70 | IMDH_ECO57 | Inosine-5'-monophosphate dehydrogenase | *Escherichia coli* | *gua*B | 52275.00 | 6.00 | 245 | Cell growth | [[4](#_ENREF_4)] |
| 70 | KPYK2_ECOLI | Pyruvate kinase II | *Escherichia coli* | *pyk*A | 51553 | 6.24 | 116 | Glycolysis | [[7](#_ENREF_7)] |
| 70 | YAFM_ECOLI | Uncharacterized protein YafM | *Escherichia coli* | *yaf*M | 20143 | 10.05 | 30 | DNA recombination | [[7](#_ENREF_7)] |
| 73 | DLDH_ECO57 | Dihydrolipoyl dehydrogenase | *Escherichia coli* | *lpd*A | 50942.00 | 5.80 | 275 | Cell redox homeostasis | [[4](#_ENREF_4)] |
| 73 | KKA8_ECOLX | Aminoglycoside 3'-phosphotransferase | *Escherichia coli* | *aphA* | 30371 | 5.06 | 55 | Antibiotic response (kanamycin and structurally-related aminoglycosides, including amikacin) | [[12](#_ENREF_12)] |
| 75 | ATPA_ECO24 | ATP synthase subunit alpha | *Escherichia coli* | *atp*A | 55416.00 | 5.70 | 363 | ATP hydrolysis | [[1](#_ENREF_1)] |
| 76 | FUMB_ECOLI | Fumarate hydratase class I, anaerobic | *Escherichia coli* | *fum*B | 60581.00 | 5.90 | 249 | Cellular response to DNA damage stimulus | [[5](#_ENREF_5)] |
| 76 | FUMA_ECO57 | Fumarate hydratase class I, aerobic | *Escherichia coli* | *fum*A | 60773 | 6.11 | 107 | Tricarboxylic acid cycle | [[4](#_ENREF_4)] |
| 78 | KPYK1_ECO57 | Pyruvate kinase I | *Escherichia coli* | *pyk*F | 51039.00 | 5.70 | 249 | Glycolytic process | [[4](#_ENREF_4)] |
| 79 | DCEA_ECOL6 | Glutamate decarboxylase alpha | *Escherichia coli* | *gad*A | 53221.00 | 5.10 | 232 | Glutamate metabolic process | [[3](#_ENREF_3)] |
| 80 | AHPF_ECOLI | Alkyl hydroperoxide reductase subunit F | *Escherichia coli* | *ahp*F | 56484.00 | 5.40 | 266 | Response to DNA damage by alkyl hydroperoxides | [[5](#_ENREF_5)] |
| 80 | KTHY_ECO81 | Thymidylate kinase | *Escherichia coli* | *tmk* | 23687 | 5.22 | 36 | dTDP biosynthetic process | [[9](#_ENREF_9)] |
| 83 | DCEB_ECO57 | Glutamate decarboxylase beta | *Escherichia coli* | *gad*B | 53204.00 | 5.20 | 221 | Glutamate metabolic process | [[4](#_ENREF_4)] |
| 84 | ENO_ECO24 | Enolase | *Escherichia coli* | *eno* | 45683.00 | 5.20 | 280 | Glycolysis | [[4](#_ENREF_4)] |
| 115 | GLYA_ECO24 | Serine hydroxymethyltransferase | *Escherichia coli* | *gly*A | 45459.00 | 6.00 | 279 | Glycine biosynthesis | [[1](#_ENREF_1)] |
| 117 | G3P3_ECO57 | Glyceraldehyde-3-phosphate dehydrogenase C | *Escherichia coli* | *gap*C | 35912.00 | 6.00 | 195 | Glycolysis | [[4](#_ENREF_4)] |
| 117 | G3P3_ECOLI | Putative glyceraldehyde-3-phosphate dehydrogenase C | *Escherichia coli* | *gap*C | 35799 | 5.65 | 195 | Glycolysis | [[7](#_ENREF_7)] |
| 118 | ISCS_ECO24 | Cysteine desulfurase | *Escherichia coli* | *isc*S | 45232.00 | 5.90 | 322 | tRNA modification | [[1](#_ENREF_1)] |
| 119 | GLMU_ECO24 | Bifunctional protein GlmU | *Escherichia coli* | *glm*U | 49388.00 | 6.10 | 154 | Cell wall organization | [[1](#_ENREF_1)] |
| 121 | TOLB_ECO24 | Protein TolB | *Escherichia coli* | *tol*B | 45927.00 | 7.70 | 50 | Proteolysis | [[1](#_ENREF_1)] |
| 121 | CARA_ECOL6 | Carbamoyl-phosphate synthase small chain | *Escherichia coli* | *car*A | 41617.00 | 6.00 | 127 | de novo' UMP biosynthesis | [[3](#_ENREF_3)] |
| 123 | G3P1_ECO57 | Glyceraldehyde-3-phosphate dehydrogenase A | *Escherichia coli* | *gap*A | 35681.00 | 6.70 | 282 | Glycolysis | [[4](#_ENREF_4)] |
| 124 | ACKA_ECO57 | Acetate kinase | *Escherichia coli* | *ack*A | 43605.00 | 5.80 | 192 | Acetyl-CoA biosynthesis | [[4](#_ENREF_4)] |
| 125 | FABF_ECO57 | 3-oxoacyl-[acyl-carrier-protein] synthase 2 | *Escherichia coli* | *fab*F | 43247.00 | 5.70 | 225 | Fatty acid biosynthesis | [[4](#_ENREF_4)] |
| 136 | ALF_ECO57 | Fructose-bisphosphate aldolase class 2 | *Escherichia coli* | *fba*A | 39351.00 | 5.50 | 147 | Glycolysis | [[4](#_ENREF_4)] |
| 137 | PEPB_ECO24 | Peptidase B | *Escherichia coli* | *pep*B | 46464.00 | 5.40 | 163 | Peptide degradation | [[1](#_ENREF_1)] |
| 140 | PGK_ECO24 | Phosphoglycerate kinase | *Escherichia coli* | *pgk* | 41264.00 | 4.90 | 276 | Glycolysis | [[1](#_ENREF_1)] |
| 141 | RPOA_ECO24 | DNA-directed RNA polymerase subunit alpha | *Escherichia coli* | *rpo*A | 36717.00 | 4.80 | 228 | Transcription | [[1](#_ENREF_1)] |
| 142 | PGK_ECO24 | Phosphoglycerate kinase | *Escherichia coli* | *pgk* | 41264.00 | 4.90 | 246 | Glycolysis | [[1](#_ENREF_1)] |
| 145 | FABB_ECOL6 | 3-oxoacyl-[acyl-carrier-protein] synthase 1 | *Escherichia coli* | *fab*B | 42928.00 | 5.20 | 144 | Fatty acid biosynthesis | [[3](#_ENREF_3)] |
| 145 | ENO_ECO24 | Enolase | *Escherichia coli* | *eno* | 45683.00 | 5.20 | 145 | Glycolysis | [[4](#_ENREF_4)] |
| 147 | EFTU2_ECO24 | Elongation factor Tu 2 | *Escherichia coli* | *tuf*2 | 43456.00 | 5.30 | 192 | Translation | [[1](#_ENREF_1)] |
| 147 | EFTU1_ECO24 | Elongation factor Tu 1 | *Escherichia coli* | *tuf*1 | 43427 | 5.18 | 176 | Translation | [[1](#_ENREF_1)] |
| 149 | EFTU1_ECO24 | Elongation factor Tu 1 | *Escherichia coli* | *tuf*1 | 43427.00 | 5.20 | 250 | Translation | [[1](#_ENREF_1)] |
| 155 | YCHF_ECO57 | Ribosome-binding ATPase YchF | *Escherichia coli* | *ych*F | 39984.00 | 4.70 | 245 | Ribosome process | [[4](#_ENREF_4)] |
| 160 | DCEA_ECOL6 | Glutamate decarboxylase alpha | *Escherichia coli* | *gad*A | 53221.00 | 5.10 | 205 | Glutamate metabolic process | [[3](#_ENREF_3)] |
| 160 | DCEB_ECO57 | Glutamate decarboxylase beta | *Escherichia coli* | *gad*A | 53204 | 5.17 | 189 | Glutamate metabolic process | [[4](#_ENREF_4)] |
| 167 | OMPA_ECO57 | Outer membrane protein A | *Escherichia coli* | *omp*A | 37292.00 | 6.00 | 114 | Conjugation | [[4](#_ENREF_4)] |
| 170 | OMPC_ECO57 | Outer membrane protein C | *Escherichia coli* | *omp*C | 40483.00 | 4.40 | 148 | Ion transport | [[4](#_ENREF_4)] |
| 171 | OMPC_ECO57 | Outer membrane protein C | *Escherichia coli* | *omp*C | 40483.00 | 4.40 | 126 | Ion transport | [[4](#_ENREF_4)] |
| 173 | OMPC_ECO57 | Outer membrane protein C | *Escherichia coli* | *omp*C | 40483.00 | 4.40 | 131 | Ion transport | [[4](#_ENREF_4)] |
| 174 | OMPC_ECO57 | Outer membrane protein C | *Escherichia coli* | *omp*C | 40483.00 | 4.40 | 126 | Ion transport | [[4](#_ENREF_4)] |
| 175 | OMPC_ECO57 | Outer membrane protein C | *Escherichia coli* | *omp*C | 40483.00 | 4.40 | 145 | Ion transport | [[4](#_ENREF_4)] |
| 178 | EFTU1_ECO24 | Elongation factor Tu 1 | *Escherichia coli* | *tuf*1 | 43427.00 | 5.20 | 200 | Translation | [[1](#_ENREF_1)] |
| 190 | ASPA_ECO57 | Aspartate ammonia-lyase | *Escherichia coli* | *asp*A | 52950.00 | 5.10 | 180 | Tricarboxylic acid cycle | [[4](#_ENREF_4)] |
| 190 | DCEA_ECOL6 | Glutamate decarboxylase alpha | *Escherichia coli* | *gad*A | 53221 | 5.10 | 101 | Glutamate metabolic process | [[3](#_ENREF_3)] |
| 215 | PFKA_ECO57 | ATP-dependent 6-phosphofructokinase isozyme 1 | *Escherichia coli* | *pfk*A | 35162.00 | 5.40 | 210 | Glycolysis | [[4](#_ENREF_4)] |
| 215 | PFKA_ECO24 | ATP-dependent 6-phosphofructokinase isozyme 1 | *Escherichia coli* | *pfk*A | 35162 | 5.38 | 210 | Glycolysis | [[1](#_ENREF_1)] |
| 216 | OMPA_ECO57 | Outer membrane protein A | *Escherichia coli* | *omp*A | 37292.00 | 6.00 | 135 | Conjugation | [[4](#_ENREF_4)] |
| 218 | ASPG2_ECOLI | L-asparaginase 2 | *Escherichia coli* | *ans*B | 36942.00 | 5.90 | 108 | Asparagine metabolic process | [[7](#_ENREF_7)] |
| 218 | PFKA_ECOL6 | ATP-dependent 6-phosphofructokinase isozyme 1 | *Escherichia coli* | *pfk*A | 35176 | 5.39 | 41 | Glycolysis | [[3](#_ENREF_3)] |
| 219 | MDH_ECO24 | Malate dehydrogenase | *Escherichia coli* | *mdh* | 32488.00 | 5.50 | 257 | Tricarboxylic acid cycle | [[1](#_ENREF_1)] |
| 222 | EFTS_ECO24 | Elongation factor Ts | *Escherichia coli* | *tsf* | 30518.00 | 5.10 | 237 | Translation | [[1](#_ENREF_1)] |
| 226 | TALB_ECOL6 | Transaldolase B | *Escherichia coli* | *tal*B | 35354.00 | 5.00 | 332 | Pentose-phosphate shunt | [[3](#_ENREF_3)] |
| 226 | FABD_ECOL6 | Malonyl CoA-acyl carrier protein transacylase | *Escherichia coli* | *fab*D | 32682.00 | 4.80 | 101 | Fatty acid biosynthesis | [[3](#_ENREF_3)] |
| 226 | TALB_ECOL6 | Transaldolase B | *Escherichia coli* | *tal*B | 35354.00 | 5.00 | 222 | Pentose-phosphate shunt | [[3](#_ENREF_3)] |
| 228 | OMPA_ECO57 | Outer membrane protein A | *Escherichia coli* | *omp*A | 37292.00 | 6.00 | 160 | Conjugation | [[4](#_ENREF_4)] |
| 230 | OMPA_ECO57 | Outer membrane protein A | *Escherichia coli* | *omp*A | 37292.00 | 6.00 | 156 | Conjugation | [[4](#_ENREF_4)] |
| 231 | TPIS_ECO24 | Triosephosphate isomerase | *Escherichia coli* | *tpi*A | 27126.00 | 5.60 | 151 | Glycolysis | [[1](#_ENREF_1)] |
| 233 | KAD_ECO24 | Adenylate kinase | *Escherichia coli* | *adk* | 23628.00 | 5.40 | 146 | Adenine nucleotide metabolism | [[1](#_ENREF_1)] |
| 234 | TSX_ECO57 | Nucleoside-specific channel-forming protein tsx | *Escherichia coli* | *tsx* | 33568.00 | 4.90 | 115 | Ion transport | [[4](#_ENREF_4)] |
| 237 | GRPE_ECO24 | Protein GrpE | *Escherichia coli* | *grp*E | 21727.00 | 4.50 | 140 | Stress response | [[1](#_ENREF_1)] |
| 244 | AHPC_ECO57 | Alkyl hydroperoxide reductase subunit C | *Escherichia coli* | *ahp*C | 20862.00 | 4.90 | 170 | Oxidative stress response | [[4](#_ENREF_4)] |
| 245 | IPYR_ECO57 | Inorganic pyrophosphatase | *Escherichia coli* | *ppa* | 19805.00 | 4.90 | 143 | Phosphate-containing compound metabolic process | [[4](#_ENREF_4)] |
| 253 | DEOD_ECO24 | Purine nucleoside phosphorylase DeoD-type | *Escherichia coli* | *deo*D | 26161.00 | 5.30 | 192 | Purine nucleoside metabolic process | [[1](#_ENREF_1)] |
| 254 | SODF_ECO57 | Superoxide dismutase [Fe] | *Escherichia coli* | *sod*B | 21310.00 | 5.50 | 95 | Superoxide dismutation | [[4](#_ENREF_4)] |
| 254 | GLNH_ECO57 | Glutamine-binding periplasmic protein | *Escherichia coli* | *gln*H | 27173 | 9.01 | 61 | Amino acid transport | [[4](#_ENREF_4)] |
| 255 | ALKH_ECO57 | KHG/KDPG aldolase | *Escherichia coli* | *eda* | 22441.00 | 5.50 | 124 | Pyruvate biosynthesis | [[4](#_ENREF_4)] |
| 275 | UDP_ECOLI | Uridine phosphorylase | *Escherichia coli* | *udp* | 27313.00 | 5.80 | 239 |  | [[7](#_ENREF_7)] |
| 275 | YBAB_ECO24 | Nucleoid-associated protein YbaB | *Escherichia coli* | *yba*B | 12076 | 4.86 | 38 | DNA conformation | [[1](#_ENREF_1)] |
| 275 | ARNA_ECO81 | Bifunctional polymyxin resistance protein ArnA | *Escherichia coli* | *arn*A | 75031 | 6.63 | 38 | Antibiotic response | [[9](#_ENREF_9)] |
| 276 | DKGA_ECOLI | 2,5-diketo-D-gluconic acid reductase A | *Escherichia coli* | *dkg*A | 31147.00 | 6.00 | 191 | L-ascorbic acid biosynthetic process | [[5](#_ENREF_5)] |
| 277 | GPMA_ECO27 | 2,3-bisphosphoglycerate-dependent phosphoglycerate mutase | *Escherichia coli* | *gpm*A | 28539.00 | 5.80 | 206 | Glycolysis | [[8](#_ENREF_8)] |
| 287 | CYSK_ECO57 | Cysteine synthase A | *Escherichia coli* | *cys*K | 34525.00 | 5.80 | 346 | Cysteine biosynthesis | [[4](#_ENREF_4)] |
| 293 | DAPA_ECO24 | 4-hydroxy-tetrahydrodipicolinate synthase | *Escherichia coli* | *dap*A | 31549.00 | 6.00 | 146 | Lysine biosynthesis | [[1](#_ENREF_1)] |
| 304 | RS2_ECO27 | 30S ribosomal protein S2 | *Escherichia coli* | *rps*B | 26784.00 | 6.70 | 146 | Translation | [[8](#_ENREF_8)] |
| 305 | CBPA_ECO24 | Curved DNA-binding protein | *Escherichia coli* | *cbp*A | 34404.00 | 6.40 | 187 | Protein folding | [[1](#_ENREF_1)] |
| 306 | KDSA_ECO24 | 2-dehydro-3-deoxyphosphooctonate aldolase | *Escherichia coli* | *kds*A | 31041.00 | 6.40 | 284 | Keto-3-deoxy-D-manno-octulosonic acid biosynthesis | [[1](#_ENREF_1)] |
| 310 | G3P1_ECO57 | Glyceraldehyde-3-phosphate dehydrogenase A | *Escherichia coli* | *gap*A | 35681.00 | 6.70 | 300 | Glycolysis | [[4](#_ENREF_4)] |
| 311 | G3P1_ECO57 | Glyceraldehyde-3-phosphate dehydrogenase A | *Escherichia coli* | *gap*A | 35681.00 | 6.70 | 258 | Glycolysis | [[4](#_ENREF_4)] |
| 330 | RRF_ECO24 | Ribosome-recycling factor | *Escherichia coli* | *frr* | 20683.00 | 7.30 | 191 | Translation | [[1](#_ENREF_1)] |
| 343 | PFLB_ECOLI | Formate acetyltransferase 1 | *Escherichia coli* | *pfl*B | 85588.00 | 5.60 | 376 | Anaerobic respiration | [[5](#_ENREF_5)] |
| 343 | RPOH_ECO57 | RNA polymerase sigma factor RpoH | *Escherichia coli* | *rpo*H | 32448 | 5.57 | 39 | Heat response | [[4](#_ENREF_4)] |
| 345 | FRDA_ECOLI | Fumarate reductase flavoprotein subunit | *Escherichia coli* | *frd*A | 66500.00 | 5.80 | 194 | Cellular response to DNA damage stimulus | [[7](#_ENREF_7)] |
| 345 | LDCI_ECO57 | Lysine decarboxylase, inducible | *Escherichia coli* | *cad*A | 81607 | 5.90 | 145 | Amino acid metabolic process | [[4](#_ENREF_4)] |
| 351 | ASPA_ECO57 | Aspartate ammonia-lyase | *Escherichia coli* | *asp*A | 52950.00 | 5.10 | 185 | Tricarboxylic acid cycle | [[4](#_ENREF_4)] |
| 351 | TOLC_ECOLI | Outer membrane protein TolC | *Escherichia coli* | *tol*C | 53708 | 5.34 | 103 | Antibiotic response | [[7](#_ENREF_7)] |

[1] Rasko DA, Rosovitz MJ, Myers GS, Mongodin EF, Fricke WF, Gajer P, et al. The pangenome structure of *Escherichia coli*: comparative genomic analysis of *E. coli* commensal and pathogenic isolates. Journal of bacteriology. 2008;190:6881-93.

[2] Johnson TJ, Kariyawasam S, Wannemuehler Y, Mangiamele P, Johnson SJ, Doetkott C, et al. The genome sequence of avian pathogenic *Escherichia coli* strain O1:K1:H7 shares strong similarities with human extraintestinal pathogenic *E. coli* genomes. Journal of bacteriology. 2007;189:3228-36.

[3] Welch RA, Burland V, Plunkett G, 3rd, Redford P, Roesch P, Rasko D, et al. Extensive mosaic structure revealed by the complete genome sequence of uropathogenic *Escherichia coli*. Proceedings of the National Academy of Sciences of the United States of America. 2002;99:17020-4.

[4] Perna NT, Plunkett G, 3rd, Burland V, Mau B, Glasner JD, Rose DJ, et al. Genome sequence of enterohaemorrhagic *Escherichia coli* O157:H7. Nature. 2001;409:529-33.

[5] Blattner FR, Plunkett G, 3rd, Bloch CA, Perna NT, Burland V, Riley M, et al. The complete genome sequence of *Escherichia coli* K-12. Science. 1997;277:1453-62.

[6] Hochhut B, Wilde C, Balling G, Middendorf B, Dobrindt U, Brzuszkiewicz E, et al. Role of pathogenicity island-associated integrases in the genome plasticity of uropathogenic *Escherichia coli* strain 536. Molecular microbiology. 2006;61:584-95.

[7] Hayashi K, Morooka N, Yamamoto Y, Fujita K, Isono K, Choi S, et al. Highly accurate genome sequences of *Escherichia coli* K-12 strains MG1655 and W3110. Molecular systems biology. 2006;2:2006 0007.

[8] Iguchi A, Thomson NR, Ogura Y, Saunders D, Ooka T, Henderson IR, et al. Complete genome sequence and comparative genome analysis of enteropathogenic *Escherichia coli* O127:H6 strain E2348/69. Journal of bacteriology. 2009;191:347-54.

[9] Touchon M, Hoede C, Tenaillon O, Barbe V, Baeriswyl S, Bidet P, et al. Organised genome dynamics in the *Escherichia coli* species results in highly diverse adaptive paths. PLoS genetics. 2009;5:e1000344.

[10] Domenighini M, Pizza M, Jobling MG, Holmes RK, Rappuoli R. Identification of errors among database sequence entries and comparison of correct amino acid sequences for the heat-labile enterotoxins of *Escherichia coli* and *Vibrio cholerae*. Molecular microbiology. 1995;15:1165-7.

[11] Cram DS, Loh SM, Cheah KC, Skurray RA. Sequence and conservation of genes at the distal end of the transfer region on plasmids F and R6-5. Gene. 1991;104:85-90.

[12] Pansegrau W, Miele L, Lurz R, Lanka E. Nucleotide sequence of the kanamycin resistance determinant of plasmid RP4: homology to other aminoglycoside 3'-phosphotransferases. Plasmid. 1987;18:193-204.
