# Supplementary material for: Multiomics Assessment of Gene Expression in a Clinical Strain of CTX-M-15-Producing ST131 Escherichia coli
Source: Front Microbiol. 2019 May 3;10:831. doi: 10.3389/fmicb.2019.00831 (PMC6509150; doi:10.3389/fmicb.2019.00831)
Supplement: TABLE S9 — Identification of protein spots from 2DE gels of periplasm extracts of ESBL-producing E. coli isolate C999 based on MALDI-TOF/MS sequencing results. [file Table_9.DOCX]

**Supplementary Table S9.** Identification of protein spots from periplasm extraction of ESBL-producing *E. coli* isolate C999 using 2-DE gels and MALDI-TOF sequencing results.

| **Spot** | **Accession** | **Protein** | **Serotype** | **Gene name** | **Protein MW** | **Protein pI** | **Mascot Score** | **Biological Process** | **Reference** |
| --- | --- | --- | --- | --- | --- | --- | --- | --- | --- |
| 1 | OMPA_ECO57 | Outer membrane protein A | *Escherichia coli* | *omp*A | 37292.00 | 6.00 | 160 | Conjugation | [[1](#_ENREF_1)] |
| 1 | PAL_ECO57 | Peptidoglycan-associated lipoprotein | *Escherichia coli* | *Pal* | 18869 | 6.37 | 41 | Bacterial envelope integrity | [[1](#_ENREF_1)] |
| 1 | THIC_ECO55 | Phosphomethylpyrimidine synthase | *Escherichia coli* | *thi*C | 71338 | 5.67 | 39 | Thiamine biosynthesis | [[2](#_ENREF_2)] |
| 2 | FLIC_ECOLI | Flagellin | *Escherichia coli* | *fli*C | 51265.00 | 4.30 | 90 | Bacterial-type flagellum-dependent cell mobility | [[3](#_ENREF_3)] |
| 2 | SUCC_ECO8A | Succinyl-CoA ligase [ADP-forming] subunit beta | *Escherichia coli* | *suc*C | 41638 | 5.25 | 31 | Tricarboxylic acid cycle | [[2](#_ENREF_2)] |
| 2 | YBHG_ECO24 | UPF0194 membrane protein YbhG | *Escherichia coli* | *ybh*G | 36263 | 5.29 | 30 | Transmembrane transport | [[4](#_ENREF_4)] |
| 3 | PGK_ECO24 | Phosphoglycerate kinase | *Escherichia coli* | *Pgk* | 41264.00 | 4.90 | 132 | Glycolysis | [[4](#_ENREF_4)] |
| 4 | EFTU1_ECO24 | Elongation factor Tu 1 | *Escherichia coli* | *tuf*1 | 43427.00 | 5.20 | 252 | Translation | [[4](#_ENREF_4)] |
| 5 | POTD_ECOLI | Spermidine/putrescine-binding periplasmic protein | *Escherichia coli* | *pot*D | 38842.00 | 5.10 | 113 | Polyamine transmembrane transport | [[3](#_ENREF_3)] |
| 5 | YJHR_ECOLI | Putative uncharacterized protein YjhR | *Escherichia coli* | *yjh*R | 38088 | 6.73 | 37 | Hydrolase activity | [[3](#_ENREF_3)] |
| 5 | PURK_ECOLI | N5-carboxyaminoimidazole ribonucleotide synthase | *Escherichia coli* | *pur*K | 39664 | 5.55 | 32 | de novo' IMP biosynthesis | [[3](#_ENREF_3)] |
| 6 | CH601_ECOK1 | 60 kDa chaperonin 1 | *Escherichia coli* | *gro*L1 | 57464.00 | 4.70 | 311 | Protein refolding | [[5](#_ENREF_5)] |
| 7 | DNAK_ECO24 | Chaperone protein DnaK | *Escherichia coli* | *dna*K | 69130.00 | 4.70 | 326 | Stress response | [[4](#_ENREF_4)] |
| 8 | RS1_ECO57 | 30S ribosomal protein S1 | *Escherichia coli* | *rps*A | 61235.00 | 4.70 | 97 | Translation | [[1](#_ENREF_1)] |
| 8 | HIS6_ECO24 | Imidazole glycerol phosphate synthase subunit HisF | *Escherichia coli* | *his*F | 28708 | 4.89 | 30 | Histidine biosynthesis | [[4](#_ENREF_4)] |
| 9 | DCEA_ECOL6 | Glutamate decarboxylase alpha | *Escherichia coli* | *gad*A | 53221.00 | 5.10 | 101 | Glutamate metabolism | [[6](#_ENREF_6)] |
| 9 | DCEB_ECO57 | Glutamate decarboxylase beta | *Escherichia coli* | *gad*B | 53204 | 5.17 | 101 | Glutamate metabolism | [[1](#_ENREF_1)] |
| 10 | DCEA_ECOL6 | Glutamate decarboxylase alpha | *Escherichia coli* | *gad*A | 53221.00 | 5.10 | 93 | Glutamate metabolism | [[6](#_ENREF_6)] |
| 10 | DCEB_ECO57 | Glutamate decarboxylase beta | *Escherichia coli* | *gad*B | 53204 | 5.17 | 93 | Glutamate metabolism | [[1](#_ENREF_1)] |
| 11 | YGGS_ECO57 | UPF0001 protein YggS | *Escherichia coli* | *ygg*S | 25885.00 | 6.10 | 52 |  | [[1](#_ENREF_1)] |
| 11 | LIGB_ECO45 | DNA ligase B | *Escherichia coli* | *lig*B | 63474.00 | 10.00 | 33 | DNA replication | [[2](#_ENREF_2)] |
| 11 | HTPG_ECO24 | Chaperone protein HtpG | *Escherichia coli* | *htp*G | 71404.00 | 4.90 | 44 | Stress response | [[4](#_ENREF_4)] |
| 12 | PNP_ECO24 | Polyribonucleotide nucleotidyltransferase | *Escherichia coli* | *Pnp* | 77111.00 | 4.90 | 80 | mRNA degradation | [[4](#_ENREF_4)] |
| 13 | PTA_ECOLI | Phosphate acetyltransferase | *Escherichia coli* | *Pta* | 77466.00 | 5.20 | 117 | Acetate biosynthesis | [[7](#_ENREF_7)] |
| 13 | TRAL5_ECOLX | Protein TraL | *Escherichia coli* | *tra*L | 26439 | 5.48 | 36 | Conjugation | [[8](#_ENREF_8)] |
| 13 | SAFA_ECOLC | Two-component-system connector protein SafA | *Escherichia coli* | *saf*A | 7267 | 9.44 | 32 | Stress response | [[9](#_ENREF_9)] |
| 14 | CLPB_ECO57 | Chaperone protein ClpB | *Escherichia coli* | *clp*B | 95697.00 | 5.30 | 241 | Heat response | [[1](#_ENREF_1)] |
| 14 | DACC_ECOLI | D-alanyl-D-alanine carboxypeptidase DacC | *Escherichia coli* | *dac*C | 43638 | 8.75 | 37 | Cell wall organization | [[3](#_ENREF_3)] |
| 14 | ALSK_ECOLI | D-allose kinase | *Escherichia coli* | *als*K | 34312 | 6.63 | 38 | Carbohydrate phosphorylation | [[3](#_ENREF_3)] |
| 15 | ODP1_ECO57 | Pyruvate dehydrogenase E1 component | *Escherichia coli* | *ace*E | 99948.00 | 5.40 | 102 | Glycolysis | [[1](#_ENREF_1)] |
| 15 | RFAJ_ECOLI | Lipopolysaccharide 1,2-glucosyltransferase | *Escherichia coli* | *rfa*J | 39358 | 9.50 | 38 | Lipopolysaccharide core region biosynthesis | [[3](#_ENREF_3)] |
| 15 | FLIY_ECOL6 | Cystine-binding periplasmic protein | *Escherichia coli* | *fli*Y | 29021 | 6.24 | 32 | Transport | [[6](#_ENREF_6)] |
| 16 | EFG_ECO24 | Elongation factor G | *Escherichia coli* | *fus*A | 77704.00 | 5.10 | 122 | Translation | [[4](#_ENREF_4)] |
| 17 | IPYR_ECO57 | Inorganic pyrophosphatase | *Escherichia coli* | *Ppa* | 19805.00 | 4.90 | 31 | Phosphate-containing compound metabolism | [[1](#_ENREF_1)] |
| 17 | MAMA_ECO57 | Methylaspartate mutase S chain | *Escherichia coli* |  | 16647.00 | 4.70 | 32 |  | [[1](#_ENREF_1)] |
| 17 | ARTI_ECOLI | Putative ABC transporter arginine-binding protein 2 | *Escherichia coli* | *art*I | 27027.00 | 5.70 | 38 | Amino acid transport | [[3](#_ENREF_3)] |
| 17 | HOFB_ECOLI | Protein transport protein HofB homolog | *Escherichia coli* | *hof*B | 51154.00 | 6.20 | 38 | Transport | [[3](#_ENREF_3)] |
| 18 | RFAJ_ECOLI | Lipopolysaccharide 1,2-glucosyltransferase | *Escherichia coli* | *rfa*J | 39358.00 | 9.50 | 30 | Lipopolysaccharide core region biosynthesis | [[3](#_ENREF_3)] |
| 18 | ARTI_ECOLI | Putative ABC transporter arginine-binding protein 2 | *Escherichia coli* | *art*I | 27027.00 | 5.70 | 60 | Amino acid transport | [[3](#_ENREF_3)] |
| 18 | YJHR_ECOLI | Putative uncharacterized protein YjhR | *Escherichia coli* | *yjh*R | 38088 | 6.73 | 38 | Hydrolase activity | [[3](#_ENREF_3)] |
| 19 | FLIY_ECOL6 | Cystine-binding periplasmic protein | *Escherichia coli* | *fli*Y | 29021.00 | 6.20 | 123 | Transport | [[6](#_ENREF_6)] |
| 19 | MATA_ECO81 | HTH-type transcriptional regulator MatA | *Escherichia coli* | *ecp*R | 23428 | 9.84 | 31 | Transcription | [[2](#_ENREF_2)] |
| 19 | MEPS_ECO57 | Murein DD-endopeptidase MepS/Murein LD-carboxypeptidase | *Escherichia coli* | *mep*S | 21141 | 10.54 | 25 | Capsule polysaccharide biosynthesis | [[1](#_ENREF_1)] |
| 19 | NORR_ECO24 | Anaerobic nitric oxide reductase transcription regulator NorR | *Escherichia coli* | *nor*R | 55515 | 5.56 | 29 | Transcription | [[4](#_ENREF_4)] |
| 20 | BLAT_ECOLX | Beta-lactamase TEM | *Escherichia coli* | *Bla* | 31666.00 | 5.60 | 170 | Antibiotic response | [[10](#_ENREF_10)] |
| 20 | VHTJ_ECOL6 | Lambda prophage-derived head-to-tail joining protein W | *Escherichia coli* | *c3172* | 7608 | 10.37 | 42 | Viral life cycle | [[6](#_ENREF_6)] |
| 21 | ULAC_ECO57 | Ascorbate-specific phosphotransferase enzyme IIA component | *Escherichia coli* | *ula*C | 17284.00 | 4.40 | 30 | Transport | [[1](#_ENREF_1)] |
| 21 | YGAV_ECOLI | Probable HTH-type transcriptional regulator YgaV | *Escherichia coli* | *yga*V | 10704.00 | 9.80 | 31 | Transcription | [[1](#_ENREF_1)] |
| 22 | PURK_ECOLI | N5-carboxyaminoimidazole ribonucleotide synthase | *Escherichia coli* | *pur*K | 39664.00 | 5.50 | 29 | de novo' IMP biosynthesis | [[3](#_ENREF_3)] |
| 22 | YGFZ_ECOL6 | tRNA-modifying protein YgfZ | *Escherichia coli* | *ygf*Z | 36350.00 | 5.10 | 29 | tRNA processing | [[6](#_ENREF_6)] |
| 23 | FOLM_ECO24 | Dihydrofolate reductase FolM | *Escherichia coli* | *fol*M | 26489.00 | 8.00 | 25 | One-carbon metabolism | [[4](#_ENREF_4)] |
| 23 | HOFB_ECOLI | Protein transport protein HofB homolog | *Escherichia coli* | *hof*B | 51154.00 | 6.20 | 33 | Transport | [[3](#_ENREF_3)] |
| 23 | MARC_ECO24 | UPF0056 inner membrane protein MarC | *Escherichia coli* | *mar*C | 23659.00 | 9.10 | 25 |  | [[4](#_ENREF_4)] |
| 23 | RT86_ECOLX | RNA-directed DNA polymerase from retron EC86 | *Escherichia coli* |  | 36686.00 | 10.60 | 32 | RNA-directed DNA polymerase activity | [[11](#_ENREF_11)] |
| 23 | RL2_ECO24 | 50S ribosomal protein L2 | *Escherichia coli* | *rpl*B | 29956.00 | 11.60 | 32 | Translation | [[4](#_ENREF_4)] |
| 23 | YAFT_ECOLI | Uncharacterized lipoprotein YafT | *Escherichia coli* | *yaf*T | 29872.00 | 6.00 | 35 |  | [[3](#_ENREF_3)] |
| 24 | MINE_ECO24 | Cell division topological specificity factor | *Escherichia coli* | *min*E | 10286.00 | 5.00 | 27 | Cell division | [[4](#_ENREF_4)] |
| 24 | UBIB_ECO27 | Probable ubiquinone biosynthesis protein UbiB | *Escherichia coli* | *ubi*B | 63317.00 | 9.50 | 40 | Ubiquinone biosynthesis | [[12](#_ENREF_12)] |
| 25 | ADHE_ECO57 | Aldehyde-alcohol dehydrogenase | *Escherichia coli* | *adh*E | 96580.00 | 6.30 | 73 | Acetaldehyde dehydrogenase activity | [[1](#_ENREF_1)] |
| 25 | CEIB_ECOLX | Colicin-Ib | *Escherichia coli* | *Cib* | 69881 | 9.44 | 43 | Defense response to Gram-negative bacterium | [[13](#_ENREF_13)] |
| 25 | CYSH_ECO24 | Phosphoadenosine phosphosulfate reductase | *Escherichia coli* | *cys*H | 28012.00 | 5.40 | 38 | Cysteine biosynthesis | [[4](#_ENREF_4)] |
| 25 | ILVD_ECO45 | Dihydroxy-acid dehydratase | *Escherichia coli* | *ilv*D | 66199.00 | 5.40 | 40 | Isoleucine biosynthesis | [[2](#_ENREF_2)] |
| 25 | YEAO_ECOLI | Uncharacterized protein YeaO | *Escherichia coli* | *yea*O | 13435.00 | 6.40 | 51 |  | [[3](#_ENREF_3)] |
| 26 | YGGE_ECO57 | Uncharacterized protein YggE | *Escherichia coli* | *ygg*E | 26619.00 | 6.10 | 61 |  | [[1](#_ENREF_1)] |
| 26 | ILVD_ECOLU | Dihydroxy-acid dehydratase | *Escherichia coli* | *ilv*D | 66202 | 5.52 | 50 | Isoleucine biosynthesis | [[2](#_ENREF_2)] |
| 26 | MSCM_ECOLI | Miniconductance mechanosensitive channel MscM | *Escherichia coli* | *msc*M | 124232.00 | 6.60 | 48 | Cellular response to osmotic stress | [[3](#_ENREF_3)] |
| 26 | RS19_ECO24 | 30S ribosomal protein S19 | *Escherichia coli* | *rps*S | 10424.00 | 11.00 | 51 | Translation | [[4](#_ENREF_4)] |
| 27 | CEIB_ECOLX | Colicin-Ib | *Escherichia coli* | *Cib* | 69881.00 | 9.40 | 37 | Defense response to Gram-negative bacterium | [[13](#_ENREF_13)] |
| 27 | YEAO_ECOLI | Uncharacterized protein YeaO | *Escherichia coli* | *yea*O | 13435.00 | 6.40 | 52 |  | [[3](#_ENREF_3)] |
| 28 | ENO_ECO24 | Enolase | *Escherichia coli* | *Eno* | 45683.00 | 5.20 | 50 | Glycolysis | [[4](#_ENREF_4)] |
| 28 | FLIY_ECOL6 | Cystine-binding periplasmic protein | *Escherichia coli* | *fli*Y | 29021.00 | 6.20 | 41 | Transport | [[6](#_ENREF_6)] |
| 28 | YGFZ_ECOL6 | tRNA-modifying protein YgfZ | *Escherichia coli* | *ygf*Z | 36350.00 | 5.10 | 48 | RNA modification | [[6](#_ENREF_6)] |
| 29 | YCGV_ECO57 | Putative uncharacterized protein YcgV | *Escherichia coli* | *ycg*V | 55491.00 | 4.60 | 38 |  | [[1](#_ENREF_1)] |
| 29 | YEDE_ECOLI | UPF0394 inner membrane protein YedE | *Escherichia coli* | *yed*E | 44627.00 | 10.70 | 41 |  | [[3](#_ENREF_3)] |
| 29 | HOFB_ECOLI | Protein transport protein HofB homolog | *Escherichia coli* | *hof*B | 51154.00 | 6.20 | 38 | Transport | [[3](#_ENREF_3)] |
| 30 | ILVD_ECO27 | Dihydroxy-acid dehydratase | *Escherichia coli* | *ilv*D | 66143.00 | 5.50 | 26 | Isoleucine biosynthesis | [[12](#_ENREF_12)] |
| 30 | PURK_ECOLI | N5-carboxyaminoimidazole ribonucleotide synthase | *Escherichia coli* | *pur*K | 39664.00 | 5.50 | 29 | de novo' IMP biosynthesis | [[3](#_ENREF_3)] |
| 30 | CSE1_ECOLI | CRISPR system Cascade subunit CasA | *Escherichia coli* | *cas*A | 56493.00 | 9.50 | 33 | Defense response to virus | [[7](#_ENREF_7)] |
| 30 | HIS4_ECOK1 | 1-(5-phosphoribosyl)-5-[(5-phosphoribosylamino)methylideneamino] imidazole-4-carboxamide isomerase | *Escherichia coli* | *his*A | 26170.00 | 4.90 | 33 | Histidine biosynthesis | [[5](#_ENREF_5)] |
| 30 | RFBK9_ECOLX | Phosphomannomutase | *Escherichia coli* | *man*B | 50677.00 | 5.20 | 36 | GDP-mannose biosynthesis | [[14](#_ENREF_14)] |
| 31 | YCIN_ECO57 | Protein YciN | *Escherichia coli* | *yci*N | 9380.00 | 5.40 | 20 |  | [[1](#_ENREF_1)] |
| 31 | PURK_ECOLI | N5-carboxyaminoimidazole ribonucleotide synthase | *Escherichia coli* | *pur*K | 39664.00 | 5.50 | 33 | de novo' IMP biosynthesis | [[3](#_ENREF_3)] |
| 31 | YGFZ_ECOL6 | tRNA-modifying protein YgfZ | *Escherichia coli* | *ygf*Z | 36350.00 | 5.10 | 33 | RNA modification | [[6](#_ENREF_6)] |
| 32 | PURK_ECOLI | N5-carboxyaminoimidazole ribonucleotide synthase | *Escherichia coli* | *pur*K | 39664.00 | 5.50 | 34 | de novo' IMP biosynthesis | [[3](#_ENREF_3)] |
| 32 | YGFZ_ECOL6 | tRNA-modifying protein YgfZ | *Escherichia coli* | *ygf*Z | 36350.00 | 5.10 | 34 | RNA modification | [[6](#_ENREF_6)] |
| 32 | YOBB_ECOLI | Uncharacterized protein YobB | *Escherichia coli* | *yob*B | 24722.00 | 10.00 | 35 |  | [[7](#_ENREF_7)] |
| 35 | SYK2_ECO57 | Lysine--tRNA ligase, heat inducible | *Escherichia coli* | *lys*U | 57847.00 | 5.00 | 60 | Protein biosynthesis | [[1](#_ENREF_1)] |
| 36 | PURK_ECOLI | N5-carboxyaminoimidazole ribonucleotide synthase | *Escherichia coli* | *pur*K | 39664.00 | 5.50 | 34 | de novo' IMP biosynthesis | [[3](#_ENREF_3)] |
| 36 | YGFZ_ECOL6 | tRNA-modifying protein YgfZ | *Escherichia coli* | *ygf*Z | 36350.00 | 5.10 | 34 | RNA modification | [[6](#_ENREF_6)] |
| 37 | OMPC_ECO57 | Outer membrane protein C | *Escherichia coli* | *omp*C | 40483.00 | 4.40 | 47 | Ion transport | [[1](#_ENREF_1)] |
| 37 | MCBB_ECOLX | Microcin B17-processing protein McbB | *Escherichia coli* | *mcb*B | 34363.00 | 8.70 | 33 | Antibiotic biosynthesis | [[15](#_ENREF_15)] |
| 38 | DIAA_ECO24 | DnaA initiator-associating protein DiaA | *Escherichia coli* | *dia*A | 21362.00 | 5.40 | 39 | DNA replication | [[4](#_ENREF_4)] |
| 38 | FLIY_ECOL6 | Cystine-binding periplasmic protein | *Escherichia coli* | *fli*Y | 29021.00 | 6.20 | 44 | Transport | [[6](#_ENREF_6)] |
| 39 | HSLJ_ECOLI | Heat shock protein HslJ | *Escherichia coli* | *hsl*J | 15327.00 | 7.70 | 30 | Heat response | [[3](#_ENREF_3)] |
| 39 | SURA_ECO57 | Chaperone SurA | *Escherichia coli* | *sur*A | 47254.00 | 6.50 | 34 | Outer membrane protein folding | [[1](#_ENREF_1)] |
| 40 | TOLB_ECO24 | Protein TolB | *Escherichia coli* | *tol*B | 45927.00 | 7.70 | 100 | Proteolysis | [[4](#_ENREF_4)] |
| 41 | ATPB_ECO24 | ATP synthase subunit beta | *Escherichia coli* | *atp*D | 50351.00 | 4.80 | 52 | ATP hydrolysis | [[4](#_ENREF_4)] |
| 41 | BICB_ECOLI | Putative uncharacterized protein BicB | *Escherichia coli* | *bic*B | 19369.00 | 11.20 | 26 |  | [[16](#_ENREF_16)] |
| 41 | YGGN_ECO57 | Uncharacterized protein YggN | *Escherichia coli* | *ygg*N | 26527.00 | 9.60 | 35 |  | [[1](#_ENREF_1)] |
| 43 | YMFI_ECOLI | Uncharacterized protein YmfI | *Escherichia coli* | *ymf*I | 13159.00 | 4.70 | 18 |  | [[7](#_ENREF_7)] |
| 43 | RLMC_ECO24 | 23S rRNA (uracil(747)-C(5))-methyltransferase RlmC | *Escherichia coli* | *rml*C | 42561.00 | 6.70 | 29 | rRNA (uridine-C5-)-methyltransferase activity | [[4](#_ENREF_4)] |
| 43 | YGGN_ECO57 | Uncharacterized protein YggN | *Escherichia coli* | *ygg*N | 26527.00 | 9.60 | 37 |  | [[1](#_ENREF_1)] |
| 43 | GSIC_ECO57 | Glutathione transport system permease protein GsiC | *Escherichia coli* | *gsi*C | 34113.00 | 9.00 | 30 | Transport | [[1](#_ENREF_1)] |
| 44 | RS21_ECO24 | 30S ribosomal protein S21 | *Escherichia coli* | *rps*U | 8552.00 | 11.80 | 24 | Translation | [[4](#_ENREF_4)] |
| 44 | YOBF_ECO57 | Uncharacterized protein YobF | *Escherichia coli* | *yob*F | 5380.00 | 9.80 | 25 |  | [[1](#_ENREF_1)] |
| 45 | YCGV_ECO57 | Putative uncharacterized protein YcgV | *Escherichia coli* | *ycg*V | 55491.00 | 4.60 | 24 |  | [[1](#_ENREF_1)] |
| 45 | FLIY_ECOL6 | Cystine-binding periplasmic protein | *Escherichia coli* | *fli*Y | 29021.00 | 6.20 | 27 | Transport | [[6](#_ENREF_6)] |
| 45 | MALK_ECOL5 | Maltose/maltodextrin import ATP-binding protein MalK | *Escherichia coli* | *mal*K | 41108.00 | 6.10 | 26 | Transport | [[17](#_ENREF_17)] |
| 46 | 6PGD_ECOLI | 6-phosphogluconate dehydrogenase, decarboxylating | *Escherichia coli* | *gnd* | 51563.00 | 4.90 | 46 | Pentose-phosphate shunt | [[7](#_ENREF_7)] |
| 48 | GSIC_ECO57 | Glutathione transport system permease protein GsiC | *Escherichia coli* | *gsi*C | 34113.00 | 9.00 | 25 | Transport | [[1](#_ENREF_1)] |
| 48 | MATA_ECO57 | HTH-type transcriptional regulator MatA | *Escherichia coli* | *ecp*R | 23399.00 | 9.70 | 25 | Transcription | [[1](#_ENREF_1)] |
| 49 | ASPA_ECO57 | Aspartate ammonia-lyase | *Escherichia coli* | *asp*A | 52950.00 | 5.10 | 75 | Tricarboxylic acid cycle | [[1](#_ENREF_1)] |
| 49 | MOBB_ECOLI | Molybdopterin-guanine dinucleotide biosynthesis adapter protein | *Escherichia coli* | *mob*B | 19408 | 5.24 | 28 | Mo-molybdopterin cofactor biosynthesis | [[3](#_ENREF_3)] |
| 49 | MAMA_ECO57 | Methylaspartate mutase S chain | *Escherichia coli* |  | 16647.00 | 4.70 | 29 |  | [[1](#_ENREF_1)] |
| 51 | GUTM_ECOLI | Glucitol operon activator protein | *Escherichia coli* | *gut*M | 13059.00 | 11.70 | 28 | Transcription | [[3](#_ENREF_3)] |
| 51 | KLEE1_ECOLX | Protein KleE | *Escherichia coli* | *kle*E | 11957.00 | 10.50 | 27 |  | [[18](#_ENREF_18)] |
| 51 | CPXP_ECOL6 | Periplasmic protein CpxP | *Escherichia coli* | *cpx*P | 18953.00 | 6.40 | 40 |  | [[6](#_ENREF_6)] |
| 51 | KPTA_ECOL5 | Probable RNA 2'-phosphotransferase | *Escherichia coli* | *kpt*A | 20602.00 | 9.90 | 38 | tRNA splicing | [[17](#_ENREF_17)] |
| 51 | LLDD_ECO24 | L-lactate dehydrogenase [cytochrome] | *Escherichia coli* | *lld*D | 42902.00 | 6.40 | 44 | Lactate oxidation | [[4](#_ENREF_4)] |
| 52 | FADJ_ECOLC | Fatty acid oxidation complex subunit alpha | *Escherichia coli* | *fad*J | 77634.00 | 9.60 | 34 | Fatty-acid beta-oxidation | [[9](#_ENREF_9)] |
| 52 | FENR_ECOLI | Ferredoxin--NADP reductase | *Escherichia coli* | *fpr* | 27847.00 | 6.20 | 23 | Drug response | [[3](#_ENREF_3)] |
| 52 | LPHI_ECOL6 | His operon leader peptide | *Escherichia coli* | *his*L | 2080.00 | 9.90 | 45 | Histidine biosynthesis | [[6](#_ENREF_6)] |
| 52 | PCNB_ECO57 | Poly(A) polymerase I | *Escherichia coli* | *pcn*B | 54065.00 | 10.20 | 28 | Transcription | [[1](#_ENREF_1)] |
| 52 | LLDD_ECO24 | L-lactate dehydrogenase [cytochrome] | *Escherichia coli* | *lld*D | 42902.00 | 6.40 | 33 | Lactate oxidation | [[4](#_ENREF_4)] |
| 53 | FEOA_ECO57 | Ferrous iron transport protein A | *Escherichia coli* | *feo*A | 8423.00 | 10.10 | 24 | Ion transport | [[1](#_ENREF_1)] |
| 53 | RS21_ECO24 | 30S ribosomal protein S21 | *Escherichia coli* | *rps*U | 8552.00 | 11.80 | 23 | Translation | [[4](#_ENREF_4)] |
| 53 | KBAZ_ECO7I | D-tagatose-1,6-bisphosphate aldolase subunit KbaZ | *Escherichia coli* | *kba*Z | 47591.00 | 5.20 | 45 | Galactitol metabolism | [[2](#_ENREF_2)] |
| 54 | FENR_ECOLI | Ferredoxin--NADP reductase | *Escherichia coli* | *fpr* | 27847.00 | 6.20 | 22 | Drug response | [[3](#_ENREF_3)] |
| 54 | YGIN_ECO57 | Probable quinol monooxygenase YgiN | *Escherichia coli* | *ygi*N | 11639.00 | 5.80 | 29 | Oxidoreductase activity | [[1](#_ENREF_1)] |
| 54 | URED_ECO57 | Putative urease accessory protein UreD homolog | *Escherichia coli* | *ure*D1 | 27859.00 | 6.00 | 37 | Nitrogen compound metabolism | [[1](#_ENREF_1)] |
| 55 | YCIN_ECO57 | Protein YciN | *Escherichia coli* | *yci*N | 9380.00 | 5.40 | 20 |  | [[1](#_ENREF_1)] |
| 55 | PURK_ECOLI | N5-carboxyaminoimidazole ribonucleotide synthase | *Escherichia coli* | *pur*K | 39664.00 | 5.50 | 33 | de novo' IMP biosynthesis | [[3](#_ENREF_3)] |
| 55 | YDFV_ECOLI | Uncharacterized protein YdfV | *Escherichia coli* | *ydf*V | 11472.00 | 11.00 | 47 |  | [[7](#_ENREF_7)] |
| 55 | YGFZ_ECOL6 | tRNA-modifying protein YgfZ | *Escherichia coli* | *ygf*Z | 36350.00 | 5.10 | 33 | RNA modification | [[6](#_ENREF_6)] |
| 56 | DACC_ECOLI | D-alanyl-D-alanine carboxypeptidase DacC | *Escherichia coli* | *dac*C | 43638.00 | 8.70 | 29 | Drug response | [[3](#_ENREF_3)] |
| 56 | SGRT_ECOLI | Putative inhibitor of glucose uptake transporter SgrT | *Escherichia coli* | *sgr*T | 5392.00 | 9.40 | 50 | Enzyme inhibitor activity | [[7](#_ENREF_7)] |
| 57 | YCIN_ECO57 | Protein YciN | *Escherichia coli* | *yci*N | 9380.00 | 5.40 | 20 |  | [[1](#_ENREF_1)] |
| 57 | RUVC_ECO27 | Crossover junction endodeoxyribonuclease RuvC | *Escherichia coli* | *ruv*C | 18849.00 | 10.20 | 27 | DNA recombination | [[12](#_ENREF_12)] |
| 57 | PURK_ECOLI | N5-carboxyaminoimidazole ribonucleotide synthase | *Escherichia coli* | *pur*K | 39664.00 | 5.50 | 33 | de novo' IMP biosynthesis | [[3](#_ENREF_3)] |
| 57 | OMPC_ECO57 | Outer membrane protein C | *Escherichia coli* | *omp*C | 40483.00 | 4.40 | 34 | Ion transport | [[1](#_ENREF_1)] |
| 57 | YGFZ_ECOL6 | tRNA-modifying protein YgfZ | *Escherichia coli* | *ygf*Z | 36350.00 | 5.10 | 33 | RNA modification | [[6](#_ENREF_6)] |
| 57 | KBAZ_ECO7I | D-tagatose-1,6-bisphosphate aldolase subunit KbaZ | *Escherichia coli* | *kba*Z | 47591.00 | 5.20 | 64 | Galactitol metabolism | [[2](#_ENREF_2)] |
| 58 | IMPC_ECOLX | Protein ImpC | *Escherichia coli* | *imp*C | 9496.00 | 5.20 | 21 | DNA repair | [[19](#_ENREF_19)] |
| 58 | RUVC_ECO27 | Crossover junction endodeoxyribonuclease RuvC | *Escherichia coli* | *ruv*C | 18849.00 | 10.20 | 27 | DNA recombination | [[12](#_ENREF_12)] |
| 58 | MOLR_ECOLI | Putative molybdate metabolism regulator | *Escherichia coli* | *mol*R | 141591.00 | 6.00 | 21 | Molybdate metabolism | [[7](#_ENREF_7)] |
| 58 | ARSR2_ECOLX | Arsenical resistance operon repressor | *Escherichia coli* | *ars*R | 13276.00 | 9.30 | 31 | Response to arsenic-containing substance | [[20](#_ENREF_20)] |
| 58 | YOBB_ECOLI | Uncharacterized protein YobB | *Escherichia coli* | *yob*B | 24722.00 | 10.00 | 34 |  | [[7](#_ENREF_7)] |
| 58 | URED_ECO57 | Putative urease accessory protein UreD homolog | *Escherichia coli* | *ure*D1 | 27859.00 | 6.00 | 39 | Nitrogen compound metabolism | [[1](#_ENREF_1)] |
| 59 | FEOA_ECO57 | Ferrous iron transport protein A | *Escherichia coli* | *feo*A | 8423.00 | 10.10 | 24 | Ion transport | [[1](#_ENREF_1)] |
| 59 | FENR_ECOLI | Ferredoxin--NADP reductase | *Escherichia coli* | *fpr* | 27847.00 | 6.20 | 23 | Drug response | [[3](#_ENREF_3)] |
| 59 | RS21_ECO24 | 30S ribosomal protein S21 | *Escherichia coli* | *rps*U | 8552.00 | 11.80 | 24 | Translation | [[4](#_ENREF_4)] |
| 59 | SYT_ECO24 | Threonine--tRNA ligase | *Escherichia coli* | *thr*S | 74680.00 | 5.80 | 52 | Threonyl-tRNA aminoacylation | [[4](#_ENREF_4)] |
| 60 | RUVC_ECO27 | Crossover junction endodeoxyribonuclease RuvC | *Escherichia coli* | *ruv*C | 18849.00 | 10.20 | 27 | DNA recombination | [[12](#_ENREF_12)] |
| 60 | RL2_ECO24 | 50S ribosomal protein L2 | *Escherichia coli* | *rpl*B | 29956.00 | 11.60 | 31 | Translation | [[4](#_ENREF_4)] |
| 60 | KBAZ_ECO7I | D-tagatose-1,6-bisphosphate aldolase subunit KbaZ | *Escherichia coli* | *kba*Z | 47591.00 | 5.20 | 47 | Galactitol metabolism | [[2](#_ENREF_2)] |
| 61 | YDFV_ECOLI | Uncharacterized protein YdfV | *Escherichia coli* | *ydf*V | 11472.00 | 11.00 | 30 |  | [[7](#_ENREF_7)] |
| 61 | KBAZ_ECO7I | D-tagatose-1,6-bisphosphate aldolase subunit KbaZ | *Escherichia coli* | *kba*Z | 47591.00 | 5.20 | 61 | Galactitol metabolism | [[2](#_ENREF_2)] |
| 62 | RUVC_ECO27 | Crossover junction endodeoxyribonuclease RuvC | *Escherichia coli* | *ruv*C | 18849.00 | 10.20 | 28 | DNA recombination | [[12](#_ENREF_12)] |
| 62 | OMPC_ECO57 | Outer membrane protein C | *Escherichia coli* | *omp*C | 40483.00 | 4.40 | 36 | Ion transport | [[1](#_ENREF_1)] |
| 62 | SYT_ECO24 | Threonine--tRNA ligase | *Escherichia coli* | *thr*S | 74680.00 | 5.80 | 38 | Threonyl-tRNA aminoacylation | [[4](#_ENREF_4)] |
| 63 | YCIN_ECO57 | Protein YciN | *Escherichia coli* | *yci*N | 9380.00 | 5.40 | 20 |  | [[1](#_ENREF_1)] |
| 63 | DNAC_ECO57 | DNA replication protein DnaC | *Escherichia coli* | *dna*C | 28088.00 | 9.80 | 32 | DNA replication | [[1](#_ENREF_1)] |
| 63 | PURK_ECOLI | N5-carboxyaminoimidazole ribonucleotide synthase | *Escherichia coli* | *pur*K | 39664.00 | 5.50 | 33 | de novo' IMP biosynthesis | [[3](#_ENREF_3)] |
| 63 | YGFZ_ECOL6 | tRNA-modifying protein YgfZ | *Escherichia coli* | *ygf*Z | 36350.00 | 5.10 | 33 | RNA modification | [[6](#_ENREF_6)] |
| 63 | KBAZ_ECO7I | D-tagatose-1,6-bisphosphate aldolase subunit KbaZ | *Escherichia coli* | *kba*Z | 47591.00 | 5.20 | 42 | Galactitol metabolism | [[2](#_ENREF_2)] |
| 64 | YDFV_ECOLI | Uncharacterized protein YdfV | *Escherichia coli* | *ydf*V | 11472.00 | 11.00 | 29 |  | [[7](#_ENREF_7)] |
| 64 | CYNT_ECO57 | Carbonic anhydrase 1 | *Escherichia coli* | *cyn*T | 24091.00 | 7.90 | 38 | Carbonate dehydratase activity | [[1](#_ENREF_1)] |
| 65 | KCY_ECO24 | Cytidylate kinase | *Escherichia coli* | *cmk* | 24788.00 | 5.50 | 24 | Pyrimidine nucleotide metabolism | [[4](#_ENREF_4)] |
| 65 | YGCU_ECO57 | Uncharacterized FAD-linked oxidoreductase YgcU | *Escherichia coli* | *ygc*U | 54130.00 | 6.00 | 28 | Lipid biosynthesis | [[1](#_ENREF_1)] |
| 65 | SYT_ECO24 | Threonine--tRNA ligase | *Escherichia coli* | *thr*S | 74680.00 | 5.80 | 31 | Threonyl-tRNA aminoacylation | [[4](#_ENREF_4)] |
| 66 | CHS_ECOLX | Chondroitin synthase | *Escherichia coli* | *kfo*C | 80234.00 | 6.40 | 21 | Chondroitin polymerization | [[21](#_ENREF_21)] |
| 66 | PURK_ECOLI | N5-carboxyaminoimidazole ribonucleotide synthase | *Escherichia coli* | *pur*K | 39664.00 | 5.50 | 21 | de novo' IMP biosynthesis | [[3](#_ENREF_3)] |
| 66 | RL2_ECO24 | 50S ribosomal protein L2 | *Escherichia coli* | *rpl*B | 29956.00 | 11.60 | 28 | Translation | [[4](#_ENREF_4)] |
| 66 | RUVC_ECO27 | Crossover junction endodeoxyribonuclease RuvC | *Escherichia coli* | *ruv*C | 18849.00 | 10.20 | 27 | DNA recombination | [[12](#_ENREF_12)] |
| 66 | ILVD_ECO45 | Dihydroxy-acid dehydratase | *Escherichia coli* | *ilv*D | 66199.00 | 5.40 | 33 | Isoleucine biosynthesis | [[2](#_ENREF_2)] |
| 66 | URED_ECO57 | Putative urease accessory protein UreD homolog | *Escherichia coli* | *ure*D1 | 27859.00 | 6.00 | 41 | Nitrogen compound metabolism | [[1](#_ENREF_1)] |
| 67 | LSRK_ECOHS | Autoinducer 2 kinase LsrK | *Escherichia coli* | *lsr*K | 58078.00 | 5.20 | 25 |  | [[4](#_ENREF_4)] |
| 67 | CHS_ECOLX | Chondroitin synthase | *Escherichia coli* | *kfo*C | 80234.00 | 6.40 | 33 | Chondroitin polymerization | [[21](#_ENREF_21)] |
| 67 | RL2_ECO24 | 50S ribosomal protein L2 | *Escherichia coli* | *rpl*B | 29956.00 | 11.60 | 28 | Translation | [[4](#_ENREF_4)] |
| 67 | RS15_ECO57 | 30S ribosomal protein S15 | *Escherichia coli* | *rps*O | 10291.00 | 11.10 | 32 | Translation | [[1](#_ENREF_1)] |
| 67 | URED_ECO57 | Putative urease accessory protein UreD homolog | *Escherichia coli* | *ure*D1 | 27859.00 | 6.00 | 37 | Nitrogen compound metabolism | [[1](#_ENREF_1)] |
| 68 | BOLA_ECOL6 | Protein BolA | *Escherichia coli* | *bol*A | 12043.00 | 6.20 | 26 |  | [[6](#_ENREF_6)] |
| 68 | RUVC_ECO27 | Crossover junction endodeoxyribonuclease RuvC | *Escherichia coli* | *ruv*C | 18849.00 | 10.20 | 28 | DNA recombination | [[12](#_ENREF_12)] |
| 68 | LLDD_ECO24 | L-lactate dehydrogenase [cytochrome] | *Escherichia coli* | *lld*D | 42902.00 | 6.40 | 36 | Lactate oxidation | [[4](#_ENREF_4)] |
| 68 | YCBK_ECO57 | Uncharacterized protein YcbK | *Escherichia coli* | *ycb*K | 20342.00 | 10.60 | 33 |  | [[1](#_ENREF_1)] |
| 68 | URED_ECO57 | Putative urease accessory protein UreD homolog | *Escherichia coli* | *ure*D1 | 27859.00 | 6.00 | 41 | Nitrogen compound metabolism | [[1](#_ENREF_1)] |
| 69 | LSRK_ECOHS | Autoinducer 2 kinase LsrK | *Escherichia coli* | *lsr*K | 58078.00 | 5.20 | 23 | Carbohydrate metabolism | [[4](#_ENREF_4)] |
| 69 | YDFV_ECOLI | Uncharacterized protein YdfV | *Escherichia coli* | *ydf*V | 11472.00 | 11.00 | 29 |  | [[7](#_ENREF_7)] |
| 69 | RS15_ECO57 | 30S ribosomal protein S15 | *Escherichia coli* | *rps*O | 10291.00 | 11.10 | 32 | Translation | [[1](#_ENREF_1)] |
| 69 | SYT_ECO24 | Threonine--tRNA ligase | *Escherichia coli* | *thr*S | 74680.00 | 5.80 | 46 | Threonyl-tRNA aminoacylation | [[4](#_ENREF_4)] |
| 70 | CHS_ECOLX | Chondroitin synthase | *Escherichia coli* | *kfo*C | 80234.00 | 6.40 | 30 | Chondroitin polymerization | [[21](#_ENREF_21)] |
| 70 | Y57M_ECOLX | Uncharacterized 7.3 kDa protein in Eco57IM 5'region | *Escherichia coli* |  | 7334.00 | 5.00 | 29 |  | [[22](#_ENREF_22)] |
| 70 | YIBJ_ECOLI | Putative uncharacterized protein YibJ | *Escherichia coli* | *yib*J | 26658.00 | 4.90 | 30 | Proteolysis | [[3](#_ENREF_3)] |
| 70 | RL2_ECO24 | 50S ribosomal protein L2 | *Escherichia coli* | *rpl*B | 29956.00 | 11.60 | 36 | Translation | [[4](#_ENREF_4)] |
| 71 | REPL2_ECOLX | Positive regulator of RepFIC repA1 expression | *Escherichia coli* | *rep*L | 2811.00 | 7.00 | 18 | DNA replication | [[19](#_ENREF_19)] |
| 71 | YDFV_ECOLI | Uncharacterized protein YdfV | *Escherichia coli* | *ydf*V | 11472.00 | 11.00 | 30 |  | [[7](#_ENREF_7)] |
| 71 | MARC_ECO24 | UPF0056 inner membrane protein MarC | *Escherichia coli* | *mar*C | 23659.00 | 9.10 | 36 |  | [[4](#_ENREF_4)] |
| 71 | SYT_ECO24 | Threonine--tRNA ligase | *Escherichia coli* | *thr*S | 74680.00 | 5.80 | 33 | Threonyl-tRNA aminoacylation | [[4](#_ENREF_4)] |
| 72 | RLMC_ECO24 | 23S rRNA (uracil(747)-C(5))-methyltransferase RlmC | *Escherichia coli* | *rml*C | 42561.00 | 6.70 | 24 | rRNA (uridine-C5-)-methyltransferase activity | [[4](#_ENREF_4)] |
| 72 | SYT_ECO24 | Threonine--tRNA ligase | *Escherichia coli* | *thr*S | 74680.00 | 5.80 | 38 | Threonyl-tRNA aminoacylation | [[4](#_ENREF_4)] |
| 72 | YDIO_ECO57 | Probable acyl-CoA dehydrogenase YdiO | *Escherichia coli* | *ydi*O | 43373.00 | 5.20 | 33 | Acyl-coA dehydrogenase activity | [[1](#_ENREF_1)] |
| 72 | URED_ECO57 | Putative urease accessory protein UreD homolog | *Escherichia coli* | *ure*D1 | 27859.00 | 6.00 | 37 | Nitrogen compound metabolism | [[1](#_ENREF_1)] |
| 73 | RIR2_ECO57 | Ribonucleoside-diphosphate reductase 1 subunit beta | *Escherichia coli* | *nrd*B | 43775.00 | 4.50 | 43 | DNA replication | [[1](#_ENREF_1)] |
| 73 | YBCV_ECOLI | Uncharacterized protein YbcV | *Escherichia coli* | *ybc*V | 16461.00 | 9.60 | 42 |  | [[3](#_ENREF_3)] |
| 74 | FENR_ECOLI | Ferredoxin--NADP reductase | *Escherichia coli* | *fpr* | 27847.00 | 6.20 | 25 | Drug response | [[3](#_ENREF_3)] |
| 74 | CYNT_ECO57 | Carbonic anhydrase 1 | *Escherichia coli* | *cyn*T | 24091.00 | 7.90 | 25 | Carbonate dehydratase activity | [[1](#_ENREF_1)] |
| 74 | SYT_ECO24 | Threonine--tRNA ligase | *Escherichia coli* | *thr*S | 74680.00 | 5.80 | 27 | Threonyl-tRNA aminoacylation | [[4](#_ENREF_4)] |
| 74 | YMFI_ECOLI | Uncharacterized protein YmfI | *Escherichia coli* | *ymf*I | 13159.00 | 4.70 | 30 |  | [[7](#_ENREF_7)] |
| 74 | RL2_ECO24 | 50S ribosomal protein L2 | *Escherichia coli* | *rpl*B | 29956.00 | 11.60 | 32 | Translation | [[4](#_ENREF_4)] |
| 74 | URED_ECO57 | Putative urease accessory protein UreD homolog | *Escherichia coli* | *ure*D1 | 27859.00 | 6.00 | 39 | Nitrogen compound metabolism | [[1](#_ENREF_1)] |
| 75 | CHS_ECOLX | Chondroitin synthase | *Escherichia coli* | *kfo*C | 80234.00 | 6.40 | 28 | Chondroitin polymerization | [[21](#_ENREF_21)] |
| 75 | YMFI_ECOLI | Uncharacterized protein YmfI | *Escherichia coli* | *ymf*I | 13159.00 | 4.70 | 30 |  | [[7](#_ENREF_7)] |
| 75 | RL2_ECO24 | 50S ribosomal protein L2 | *Escherichia coli* | *rpl*B | 29956.00 | 11.60 | 35 | Translation | [[4](#_ENREF_4)] |
| 75 | LLDD_ECO24 | L-lactate dehydrogenase [cytochrome] | *Escherichia coli* | *lld*D | 42902.00 | 6.40 | 34 | Lactate oxidation | [[4](#_ENREF_4)] |
| 75 | URED_ECO57 | Putative urease accessory protein UreD homolog | *Escherichia coli* | *ure*D1 | 27859.00 | 6.00 | 39 | Nitrogen compound metabolism | [[1](#_ENREF_1)] |
| 76 | FOLM_ECO24 | Dihydrofolate reductase FolM | *Escherichia coli* | *fol*M | 26489.00 | 8.00 | 42 | One-carbon metabolism | [[4](#_ENREF_4)] |
| 76 | YPB4_ECOLX | Uncharacterized 9.2 kDa protein | *Escherichia coli* |  | 9322.00 | 12.60 | 31 |  | [[23](#_ENREF_23)] |
| 76 | PCNB_ECO57 | Poly(A) polymerase I | *Escherichia coli* | *pcn*B | 54065.00 | 10.20 | 40 | Transcription | [[1](#_ENREF_1)] |
| 77 | CHS_ECOLX | Chondroitin synthase | *Escherichia coli* | *kfo*C | 80234.00 | 6.40 | 27 | Chondroitin polymerization | [[21](#_ENREF_21)] |
| 77 | YAFT_ECOLI | Uncharacterized lipoprotein YafT | *Escherichia coli* | *yaf*T | 29872.00 | 6.00 | 25 |  | [[3](#_ENREF_3)] |
| 77 | SYT_ECO24 | Threonine--tRNA ligase | *Escherichia coli* | *thr*S | 74680.00 | 5.80 | 29 | Threonyl-tRNA aminoacylation | [[4](#_ENREF_4)] |
| 77 | TRAJ5_ECOLX | Protein TraJ | *Escherichia coli* | *tra*J | 14197.00 | 9.20 | 29 | Transcription | [[24](#_ENREF_24)] |
| 77 | RL2_ECO24 | 50S ribosomal protein L2 | *Escherichia coli* | *rpl*B | 29956.00 | 11.60 | 34 | Translation | [[4](#_ENREF_4)] |
| 77 | OMPC_ECO57 | Outer membrane protein C | *Escherichia coli* | *omp*C | 40483.00 | 4.40 | 41 | Ion transport | [[1](#_ENREF_1)] |
| 78 | CHS_ECOLX | Chondroitin synthase | *Escherichia coli* | *kfo*C | 80234.00 | 6.40 | 28 | Chondroitin polymerization | [[21](#_ENREF_21)] |
| 78 | DGTP_ECO57 | Deoxyguanosinetriphosphate triphosphohydrolase | *Escherichia coli* | *dgt* | 59628.00 | 7.00 | 31 | GTP metabolism | [[1](#_ENREF_1)] |
| 78 | RL2_ECO24 | 50S ribosomal protein L2 | *Escherichia coli* | *rpl*B | 29956.00 | 11.60 | 35 | Translation | [[4](#_ENREF_4)] |
| 78 | CEIA_ECOLX | Colicin-Ia | *Escherichia coli* |  | 69387.00 | 9.60 | 34 | Defense response to Gram-negative bacterium | [[13](#_ENREF_13)] |
| 78 | KBAZ_ECO7I | D-tagatose-1,6-bisphosphate aldolase subunit KbaZ | *Escherichia coli* | *kba*Z | 47591.00 | 5.20 | 53 | Galactitol metabolism | [[2](#_ENREF_2)] |
| 79 | HPAB_ECOLX | 4-hydroxyphenylacetate 3-monooxygenase oxygenase component | *Escherichia coli* | *hpa*B | 59266.00 | 5.80 | 20 | Acyl-CoA dehydrogenase activity | [[25](#_ENREF_25)] |
| 79 | FENR_ECOLI | Ferredoxin--NADP reductase | *Escherichia coli* | *fpr* | 27847.00 | 6.20 | 22 | Drug response | [[3](#_ENREF_3)] |
| 79 | SYT_ECO24 | Threonine--tRNA ligase | *Escherichia coli* | *thr*S | 74680.00 | 5.80 | 29 | Threonyl-tRNA aminoacylation | [[4](#_ENREF_4)] |
| 79 | YGIN_ECO57 | Probable quinol monooxygenase YgiN | *Escherichia coli* | *ygi*N | 11639.00 | 5.80 | 29 | Oxidoreductase activity | [[1](#_ENREF_1)] |
| 79 | RL2_ECO24 | 50S ribosomal protein L2 | *Escherichia coli* | *rpl*B | 29956.00 | 11.60 | 34 | Translation | [[4](#_ENREF_4)] |
| 79 | URED_ECO57 | Putative urease accessory protein UreD homolog | *Escherichia coli* | *ure*D1 | 27859.00 | 6.00 | 37 | Nitrogen compound metabolism | [[1](#_ENREF_1)] |
| 80 | FENR_ECOLI | Ferredoxin--NADP reductase | *Escherichia coli* | *fpr* | 27847.00 | 6.20 | 26 | Drug response | [[3](#_ENREF_3)] |
| 80 | IMMD_ECOLX | Colicin-D immunity protein | *Escherichia coli* | *cdi* | 10165.00 | 5.00 | 23 | Bacteriocin immunity | [[26](#_ENREF_26)] |
| 80 | SYT_ECO24 | Threonine--tRNA ligase | *Escherichia coli* | *thr*S | 74680.00 | 5.80 | 29 | Threonyl-tRNA aminoacylation | [[4](#_ENREF_4)] |
| 80 | URED_ECO57 | Putative urease accessory protein UreD homolog | *Escherichia coli* | *ure*D1 | 27859.00 | 6.00 | 37 | Nitrogen compound metabolism | [[1](#_ENREF_1)] |
| 80 | RL2_ECO24 | 50S ribosomal protein L2 | *Escherichia coli* | *rpl*B | 29956.00 | 11.60 | 34 | Translation | [[4](#_ENREF_4)] |
| 80 | RMUC_ECO57 | DNA recombination protein RmuC | *Escherichia coli* | *rmu*C | 54788.00 | 5.10 | 38 | DNA recombination | [[1](#_ENREF_1)] |
| 81 | REPL2_ECOLX | Positive regulator of RepFIC repA1 expression | *Escherichia coli* | *rep*L | 2811.00 | 7.00 | 21 | DNA replication | [[19](#_ENREF_19)] |
| 81 | Y57M_ECOLX | Uncharacterized 7.3 kDa protein in Eco57IM 5'region | *Escherichia coli* |  | 7334.00 | 5.00 | 31 |  | [[22](#_ENREF_22)] |
| 81 | CHS_ECOLX | Chondroitin synthase | *Escherichia coli* | *kfo*C | 80234.00 | 6.40 | 30 | Chondroitin polymerization | [[21](#_ENREF_21)] |
| 81 | YDFV_ECOLI | Uncharacterized protein YdfV | *Escherichia coli* | *ydf*V | 11472.00 | 11.00 | 32 |  | [[7](#_ENREF_7)] |
| 81 | RL2_ECO24 | 50S ribosomal protein L2 | *Escherichia coli* | *rpl*B | 29956.00 | 11.60 | 37 | Translation | [[4](#_ENREF_4)] |
| 81 | YDIU_ECOLU | UPF0061 protein YdiU | *Escherichia coli* | *ydi*U | 54698.00 | 5.50 | 32 |  | [[2](#_ENREF_2)] |
| 82 | FENR_ECOLI | Ferredoxin--NADP reductase | *Escherichia coli* | *fpr* | 27847.00 | 6.20 | 26 | Drug response | [[3](#_ENREF_3)] |
| 82 | CHS_ECOLX | Chondroitin synthase | *Escherichia coli* | *kfo*C | 80234.00 | 6.40 | 30 | Chondroitin polymerization | [[21](#_ENREF_21)] |
| 82 | SYT_ECO24 | Threonine--tRNA ligase | *Escherichia coli* | *thr*S | 74680.00 | 5.80 | 29 | Threonyl-tRNA aminoacylation | [[4](#_ENREF_4)] |
| 82 | RL2_ECO24 | 50S ribosomal protein L2 | *Escherichia coli* | *rpl*B | 29956.00 | 11.60 | 38 | Translation | [[4](#_ENREF_4)] |
| 82 | KBAZ_ECO45 | D-tagatose-1,6-bisphosphate aldolase subunit KbaZ | *Escherichia coli* | *kba*Z | 47588.00 | 5.20 | 39 | Galactitol metabolism | [[2](#_ENREF_2)] |
| 83 | CHS_ECOLX | Chondroitin synthase | *Escherichia coli* | *kfo*C | 80234.00 | 6.40 | 42 | Chondroitin polymerization | [[21](#_ENREF_21)] |
| 84 | CHS_ECOLX | Chondroitin synthase | *Escherichia coli* | *kfo*C | 80234.00 | 6.40 | 39 | Chondroitin polymerization | [[21](#_ENREF_21)] |
| 84 | SYT_ECO24 | Threonine--tRNA ligase | *Escherichia coli* | *thr*S | 74680.00 | 5.80 | 39 | Threonyl-tRNA aminoacylation | [[4](#_ENREF_4)] |
| 85 | CHS_ECOLX | Chondroitin synthase | *Escherichia coli* | *kfo*C | 80234.00 | 6.40 | 42 | Chondroitin polymerization | [[21](#_ENREF_21)] |
| 85 | MINE_ECO24 | Cell division topological specificity factor | *Escherichia coli* | *min*E | 10286.00 | 5.00 | 25 | Cell division | [[4](#_ENREF_4)] |
| 85 | RT86_ECOLX | RNA-directed DNA polymerase from retron EC86 | *Escherichia coli* |  | 36686.00 | 10.60 | 32 | RNA-directed DNA polymerase activity | [[11](#_ENREF_11)] |
| 85 | ACCD_ECO24 | Acetyl-coenzyme A carboxylase carboxyl transferase subunit beta | *Escherichia coli* | *acc*D | 33642.00 | 8.90 | 43 | Fatty acid biosynthesis | [[4](#_ENREF_4)] |
| 86 | YBIS_ECO57 | Probable L,D-transpeptidase YbiS | *Escherichia coli* | *ybi*S | 33418.00 | 6.00 | 41 | Cell wall organization | [[1](#_ENREF_1)] |
| 86 | YGIN_ECO57 | Probable quinol monooxygenase YgiN | *Escherichia coli* | *ygi*N | 11639.00 | 5.80 | 50 | Oxidoreductase activity | [[1](#_ENREF_1)] |
| 87 | YIHM_ECOLI | Uncharacterized protein YihM | *Escherichia coli* | *yih*M | 37033.00 | 5.00 | 26 |  | [[27](#_ENREF_27)] |
| 87 | YGIN_ECO57 | Probable quinol monooxygenase YgiN | *Escherichia coli* | *ygi*N | 11639.00 | 5.80 | 35 | Oxidoreductase activity | [[1](#_ENREF_1)] |
| 87 | ILVD_ECO45 | Dihydroxy-acid dehydratase | *Escherichia coli* | *ilv*D | 66199.00 | 5.40 | 34 | Isoleucine biosynthesis | [[2](#_ENREF_2)] |
| 88 | CHS_ECOLX | Chondroitin synthase | *Escherichia coli* | *kfo*C | 80234.00 | 6.40 | 34 | Chondroitin polymerization | [[21](#_ENREF_21)] |
| 88 | YPB4_ECOLX | Uncharacterized 9.2 kDa protein | *Escherichia coli* |  | 9322.00 | 12.60 | 33 |  | [[23](#_ENREF_23)] |
| 88 | RL2_ECO24 | 50S ribosomal protein L2 | *Escherichia coli* | *rpl*B | 29956.00 | 11.60 | 42 | Translation | [[4](#_ENREF_4)] |

[1] Perna NT, Plunkett G, 3rd, Burland V, Mau B, Glasner JD, Rose DJ, et al. Genome sequence of enterohaemorrhagic *Escherichia coli* O157:H7. Nature. 2001;409:529-33.

[2] Touchon M, Hoede C, Tenaillon O, Barbe V, Baeriswyl S, Bidet P, et al. Organised genome dynamics in the *Escherichia coli* species results in highly diverse adaptive paths. PLoS genetics. 2009;5:e1000344.

[3] Hayashi K, Morooka N, Yamamoto Y, Fujita K, Isono K, Choi S, et al. Highly accurate genome sequences of *Escherichia coli* K-12 strains MG1655 and W3110. Molecular systems biology. 2006;2:2006 0007.

[4] Rasko DA, Rosovitz MJ, Myers GS, Mongodin EF, Fricke WF, Gajer P, et al. The pangenome structure of *Escherichia coli*: comparative genomic analysis of *E. coli* commensal and pathogenic isolates. Journal of bacteriology. 2008;190:6881-93.

[5] Johnson TJ, Kariyawasam S, Wannemuehler Y, Mangiamele P, Johnson SJ, Doetkott C, et al. The genome sequence of avian pathogenic *Escherichia coli* strain O1:K1:H7 shares strong similarities with human extraintestinal pathogenic *E. coli* genomes. Journal of bacteriology. 2007;189:3228-36.

[6] Welch RA, Burland V, Plunkett G, 3rd, Redford P, Roesch P, Rasko D, et al. Extensive mosaic structure revealed by the complete genome sequence of uropathogenic *Escherichia coli*. Proceedings of the National Academy of Sciences of the United States of America. 2002;99:17020-4.

[7] Blattner FR, Plunkett G, 3rd, Bloch CA, Perna NT, Burland V, Riley M, et al. The complete genome sequence of *Escherichia coli* K-12. Science. 1997;277:1453-62.

[8] Ziegelin G, Pansegrau W, Strack B, Balzer D, Kroger M, Kruft V, et al. Nucleotide sequence and organization of genes flanking the transfer origin of promiscuous plasmid RP4. DNA sequence : the journal of DNA sequencing and mapping. 1991;1:303-27.

[9] Copeland A, Lucas S, Lapidus A, Glavina del Rio T, Dalin E, Tice H, et al. Complete sequence of *Escherichia coli* C str. ATCC 8739. Submitted (FEB-2008) to the EMBL/GenBank/DDBJ databases Cited for: NUCLEOTIDE SEQUENCE [LARGE SCALE GENOMIC DNA] Strain: ATCC 8739 / DSM 1576 / Crooks.

[10] Brun T, Peduzzi J, Canica MM, Paul G, Nevot P, Barthelemy M, et al. Characterization and amino acid sequence of IRT-4, a novel TEM-type enzyme with a decreased susceptibility to beta-lactamase inhibitors. FEMS microbiology letters. 1994;120:111-7.

[11] Lim D. Structure of two retrons of *Escherichia coli* and their common chromosomal insertion site. Molecular microbiology. 1991;5:1863-72.

[12] Iguchi A, Thomson NR, Ogura Y, Saunders D, Ooka T, Henderson IR, et al. Complete genome sequence and comparative genome analysis of enteropathogenic *Escherichia coli* O127:H6 strain E2348/69. Journal of bacteriology. 2009;191:347-54.

[13] Mankovich JA, Hsu CH, Konisky J. DNA and amino acid sequence analysis of structural and immunity genes of colicins Ia and Ib. Journal of bacteriology. 1986;168:228-36.

[14] Reeves PR, Hobbs M, Valvano MA, Skurnik M, Whitfield C, Coplin D, et al. Bacterial polysaccharide synthesis and gene nomenclature. Trends in microbiology. 1996;4:495-503.

[15] Genilloud O, Moreno F, Kolter R. DNA sequence, products, and transcriptional pattern of the genes involved in production of the DNA replication inhibitor microcin B17. Journal of bacteriology. 1989;171:1126-35.

[16] Bentley J, Hyatt LS, Ainley K, Parish JH, Herbert RB, White GR. Cloning and sequence analysis of an *Escherichia coli* gene conferring bicyclomycin resistance. Gene. 1993;127:117-20.

[17] Hochhut B, Wilde C, Balling G, Middendorf B, Dobrindt U, Brzuszkiewicz E, et al. Role of pathogenicity island-associated integrases in the genome plasticity of uropathogenic *Escherichia coli* strain 536. Molecular microbiology. 2006;61:584-95.

[18] Thomas CM, Smith CA, Ibbotson JP, Johnston L, Wang N. Evolution of the korA-oriV segment of promiscuous IncP plasmids. Microbiology. 1995;141 ( Pt 5):1201-10.

[19] Sampei G, Mizobuchi K. Organization and diversification of plasmid genomes: complete nucleotide sequence of the ColIb-P9 genome. Submitted (DEC-1998) to the EMBL/GenBank/DDBJ databases Cited for: NUCLEOTIDE SEQUENCE [GENOMIC DNA].

[20] Bruhn DF, Li J, Silver S, Roberto F, Rosen BP. The arsenical resistance operon of IncN plasmid R46. FEMS microbiology letters. 1996;139:149-53.

[21] Ninomiya T, Sugiura N, Tawada A, Sugimoto K, Watanabe H, Kimata K. Molecular cloning and characterization of chondroitin polymerase from *Escherichia coli* strain K4. The Journal of biological chemistry. 2002;277:21567-75.

[22] Janulaitis A, Vaisvila R, Timinskas A, Klimasauskas S, Butkus V. Cloning and sequence analysis of the genes coding for Eco57I type IV restriction-modification enzymes. Nucleic acids research. 1992;20:6051-6.

[23] Livneh Z. Directed mutagenesis method for analysis of mutagen specificity: application to ultraviolet-induced mutagenesis. Proc Natl Acad Sci U S A. 1984;81:237-41.

[24] Thorsted PA, Macartney DP, Akhtar P, Haines AS, Ali N, Davidson P, et al. Complete sequence of the IncP beta plasmid R751: Implications for evolution and organisation of the IncP backbone. J Mol Biol. 1998;282:969-90.

[25] Xun L, Sandvik ER. Characterization of 4-hydroxyphenylacetate 3-hydroxylase (HpaB) of *Escherichia coli* as a reduced flavin adenine dinucleotide-utilizing monooxygenase. Applied and environmental microbiology. 2000;66:481-6.

[26] Roos U, Harkness RE, Braun V. Assembly of Colicin Genes from a Few DNA Fragments - Nucleotide-Sequence of Colicin-D. Molecular microbiology. 1989;3:891-902.

[27] Plunkett GI, Neeno-Eckwall EC, Glasner JD, Perna NT. ATOL: Assembling a taxonomically balanced genome-scale reconstruction of the evolutionary history of the *Enterobacteriaceae*. Submitted (MAY-2014) to the EMBL/GenBank/DDBJ databases Cited for: NUCLEOTIDE SEQUENCE Strain: ATCC 11775.
